# Bis(Benzofuran–1,3-*N*,*N*-heterocycle)s as Symmetric and Synthetic Drug Leads against Yellow Fever Virus

**DOI:** 10.3390/ijms232012675

**Published:** 2022-10-21

**Authors:** Nitesh K. Gupta, Srinivasan Jayakumar, Wen-Chieh Huang, Pieter Leyssen, Johan Neyts, Sergey O. Bachurin, Jih Ru Hwu, Shwu-Chen Tsay

**Affiliations:** 1 Department of Chemistry, Frontier Research Center on Fundamental and Applied Sciences of Matters, National Tsing Hua University, Hsinchu 300044, Taiwan; 2 Rega Institute for Medical Research, Katholieke Universiteit Leuven, Minderbroedersstraat 10, B-3000 Leuven, Belgium; 3 The Institute of Physiologically Active Compounds, Russian Academy of Sciences, Chernogolovka 142432, Russia; 4 Department of Chemistry, National Central University, Jhongli City 320317, Taiwan

**Keywords:** yellow fever virus, antiviral, benzofuran, imidazolidinone, benzimidazole, bis-conjugated compound

## Abstract

The yellow fever virus (YFV) is an emerging RNA virus and has caused large outbreaks in Africa and Central and South America. The virus is often transmitted through infected mosquitoes and spreads from area to area because of international travel. Being an acute viral hemorrhagic disease, yellow fever can be prevented by an effective, safe, and reliable vaccine, but not be eliminated. Currently, there is no antiviral drug available for its cure. Thus, two series of novel bis(benzofuran–1,3-imidazolidin-4-one)s and bis(benzofuran–1,3-benzimidazole)s were designed and synthesized for the development of anti-YFV lead candidates. Among 23 new bis-conjugated compounds, 4 of them inhibited YFV strain 17D (Stamaril) on Huh-7 cells in the cytopathic effect reduction assays. These conjugates exhibited the most compelling efficacy and selectivity with an EC_50_ of <3.54 μM and SI of >15.3. The results are valuable for the development of novel antiviral drug leads against emerging diseases.

## 1. Introduction

For individuals who are severely affected by yellow fever virus (YFV) but without treatment, up to 50% of them die according to the report of the World Health Organization (WHO) [1]. Each year there are 84,000–170,000 cases, which lead to 29,000–60,000 deaths. Primarily, YFV is transmitted by a bite from an infected *Aedes aegypti* mosquito and that gives rise to an acute viral hemorrhagic disease [2]. Yellow fever was first identified in 1647 in Barbados, an island in the Caribbean region [3]. Since then, infected people have carried the virus to other regions by global travelling. This disease is endemic in tropical areas of Africa and Central and South America. During the 17–19th centuries, yellow fever was transported to Europe and North America. Travelers who visited yellow fever endemic regions could bring the disease to non-infected areas. Thus, this disease is difficult to eradicate because of human activities and inevitable migration. Furthermore, YFV causes large outbreaks and incurs economic disruption, retrogression, and even decimated populations.

Yellow fever can be prevented by a vaccine, which provides efficacious immunity within 30 days [1]. Nevertheless, the mass vaccination still cannot ward off the potential outbreaks due to the resurgence of infected mosquitoes in heavily populated areas. Other factors such as a decreased immunity to the infection of individuals, ongoing deforestation, increasing people′s movements/urbanization, and climate change also lead this infectious disease to sustain continuously [4]. As a consequence, YFV is spreading much faster than before because of the frequent travelers from endemic regions to other parts of the world [5]. 

Currently, valid treatment for yellow fever does not exist [6]. Modern treatment is symptomatic, therefore its effectiveness is merely limited at reduction in the symptoms, such as dehydration, high fever, organ failure, etc. The patients often need to be hospitalized for comfort and preventing the risk of internal bleeding. Thus, aspirin and other nonsteroidal anti-inflammatory drugs are avoided but antibiotics can be applied for associated bacterial infections. As there is no antiviral drug available to cure yellow fever, development of a drug with potent activity against YFV becomes a global focus and concern.

Small molecules of several different classes were discovered to exhibit anti-YFV activity. Very recently, Ekins et al. [7] used machine learning models to identify new anti-YFV agents from a compound bank. They found a new pyrazolesulfonamide derivative with an EC_50_ of 3.2 μM and CC_50_ of 24 μM. In 2019, Schinazi et al. [8] revealed several nucleoside analogs with robust anti-YFV activity with EC_50_ values of 0.25–1.0 μM and SI > 100. In 2017, Nogueria et al. [9] reported that thiosemicarbazones and phthalylthiazoles can inhibit YFV replication. In 2016, van Aerschot et al. [10] found that pyrazol–pyrazine dicarboxamides inhibit YFV with EC_50_ values of 0.4–3.3 μM and SI > 34. They also synthesized imidazole-4,5- and pyrazine-2,3-dicarboxamides, which possess anti-YFV activity with an EC_50_ of 0.17 μM and SI of 67 [11]. Chang et al. [12] also found a benzodiazepine that inhibited YFV with an EC_50_ of 3.2 μM and CC_50_ > 100 μM. In 2011, Cushman et al. [13] reported the synthesis of 1,3-thiazoles for targeting flavivirus envelope proteins using a cell-based assay. Their methylthio ester and dihydroxypropylamide analogs showed antiviral activities and performed improved therapeutic indices as well as metabolic stabilities. In 2010, Julander and co-workers [14] reported that 2-*C*-methylcytidine inhibits the 17D vaccine strain of YFV in cell culture with an EC_90_ of 0.32 μg/mL and SI of 141. In 2008, Sparatore et al. [15] found some benzimidazole–benzotriazole conjugates with moderate activity against YFV. As early as 2005, Neyts et al. [16] provided persuasive evidence on the predominant mechanism of ribavirin against flaviviruses in vitro. Ribavirin is a standard antiviral drug used for the treatment of hepatitis C. Nevertheless, it was then discounted as a possible therapy of yellow fever due to the lack of improved survival in a virulent rhesus study [17]. Another antiviral drug sofosbuvir was recently suggested to be an option for the treatment of yellow fever [18]. Moreover, rifapentine, an antibiotic for the treatment of tuberculosis, is recommended to apply as an anti-YFV agent [19]. Even so, up to now no specific drug is yet available. 

Heterocyclic moieties are parts of the basic skeletons in many drugs [20]. Among them, the benzofuran moiety consisting of fused benzene and furan rings exists in many edible plants. They possess various kinds of biological activities [21,22], including analgesic [23], anti-Alzheimer′s [24], anti-arrhythmic [25], anti-inflammatory [26], anti-microbial [27], anti-oxidant [20], anti-tubercular [28], anti-tumor [29], and antiviral [30,31,32] properties. Imidazolidinone is also an important heterocycle with nitrogen-containing five-membered rings. For example, the 4-imidazolidinone derivatives exhibit a wide range of pharmacological activities, such as anti-malarial, anti-inflammatory, and anti-tumor potencies [33,34,35,36]. Al-Madi and co-workers [37] found their remarkable activity against an AIDS-related lymphoma (ARL) tumor cell line. Pinza and co-workers [38] reported potent anti-amnestic activity based on a fused 2-pyrrolidinones/4-imidazolidinone moiety. Existing drugs having an imidazolidinone ring including hetacillin [39], NNC 63-0532 [40], spiperone [41], spiroxatrine [42], etc. These drugs are used as antibiotics, nociceptoids, anti-psychotics, and alpha 1-adrenoceptors. As another class of nitrogen-containing moiety, benzimidazoles contain fused rings of benzene and imidazole. They exist in many natural and synthetic compounds, which are valuable towards breast cancer [43], leukemia [44], tumor cells [45], and various viruses (cytomegalovirus, RSV, BVDV, etc.) [46,47,48,49]. In 2009, benzimidazole–coumarin conjugates were reported with anti-HCV activity [50]. In 2006, Ishida and co-workers [51] disclosed the benzimidazole derivatives as inhibitors for HCV NS5B polymerase. In 2008, Sparatore et al. [15] published benzimidazole–benzotriazole conjugates that showed moderate activity against YFV.

**Figure 1:**
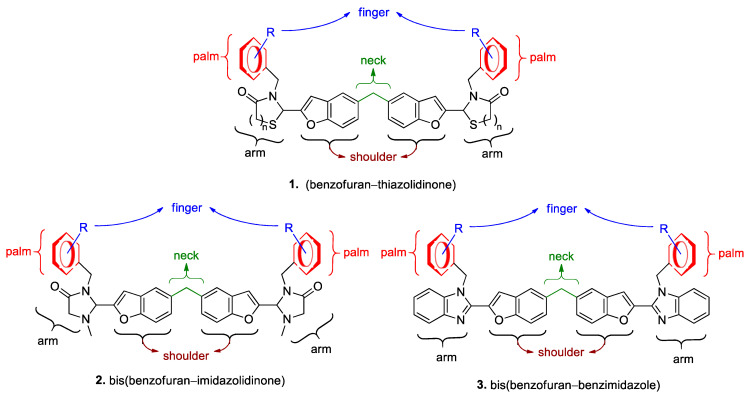
Design of targeted bis(benzofuran–imidazolidinone)s and bis(benzofuran–benzimidazole)s.

Some synthetic compounds with a dimeric and symmetric structure have been used as clinical drugs or drug candidates. These include brilacidin (an antibiotic and a COVID-19 drug candidate) [52], cromolyn (anti-asthma and anti-inflammation) [53], daclatasvir (an FDA-approved anti-HCV drug) [54], ombitasvir (anti-HCV) [55], pyrrolobenzodiazepine dimer (SJG-136, a phase 1 trial drug candidate for anti-tumor use) [56], and suramin (an essential medicine on the WHO’s list to treat African sleeping sickness and river blindness). Similar to the structural characteristics of suramin, the design and synthesis of bis(benzofuran–thiazolidinone)s and bis(benzofuran–thiazinanone)s were published in 2017 [30]. These bis-heterocycles exhibit potency against chikungunya virus with a minimum EC_50_ of 1.9 μM and SI~75 or higher. Herein, we report two new series of dimeric compounds with the skeletons of bis(benzofuran–imidazolidinone) and bis(benzofuran–benzimidazole) as anti-YFV agents. Their design was on the basis of the scaffold of bis(benzofuran–1,3-thiazolidin-4-one) **1** as shown in Figure 1 [30]. Accordingly, these nitrogen-containing bis-conjugates **2** and **3** were synthesized, and their antiviral activities were explored.

## 2. Results and Discussion

### 2.1. Syntheses of Bis(Benzofuran–Imidazolidinone) Conjugates 2 (Scheme 1) and Their Structural Identification

For construction of the imidazolidinone moiety in bis-conjugates **2a**–**k**, *N*-methylglycine (**4**) was condensed with various substituted anilines **5a**–**k**. Use of 1-[bis(dimethylamino)methylene]-1*H*-1,2,3-triazolo[4,5-b]pyridinium 3-oxide hexafluoro-phosphate (HATU) and *N*,*N*-diisopropylethylamine (DIPEA) as coupling reagents in DMF led to the best results [57]. The corresponding *N*-methyl amides **6a**–**k** were obtained as solids in 76–85% yields. Then, condensation of **6a**–**k** with bis-benzofuranal **7** [30] was performed by use of molecular sieves in Et_3_N and toluene at 110 °C [58]. Finally, the desired conjugates **2a**–**k** were produced as solids in good yields (75–87%) through dehydrative cyclization.

The structures of all new bis-conjugates **2a**–**k** were identified according to their spectroscopic data. For instance, the exact mass of compound **2e** (C_41_H_40_N_4_O_4_) was measured as 652.3052 for the species of M^+^, which is very close to its theoretical value of 652.3050. The formation of two imidazolidin-4-one rings in compound **2e** was supported by its ^1^H NMR spectrum. The two NCHN protons resonated as a singlet at 5.60 ppm; and the two sets of diastereotopic NCH_2_C=O protons resonated at *δ* 3.82 ppm as a doublet with *J*^2^ = 15.0 Hz and at *δ* 3.42 ppm as a doublet with *J*^2^ = 15.0 Hz [59]. The spectrum also showed the presence of the two methylene protons in the center of this compound, which resonated at *δ* 4.06 ppm. Two singlets at *δ* 2.44 and 2.20 ppm were associated with the methyl groups from the NCH_3_ in the imidazolidin-4-one and those attached to the 3,5-positions of *N*-phenyl group, respectively. In its ^13^C spectrum, the NC=O carbon and the NCN carbon resonated at 170.3 ppm and 78.2 ppm, respectively. A strong absorption band at 1714 cm^–1^ in its IR spectrum was corresponding to the amido C=O group. 

### 2.2. Synthesis of Bis(Benzofuran–Benzimidazole) Conjugates 3 (Scheme 2) and Their Structural Identification

**Scheme 2:**
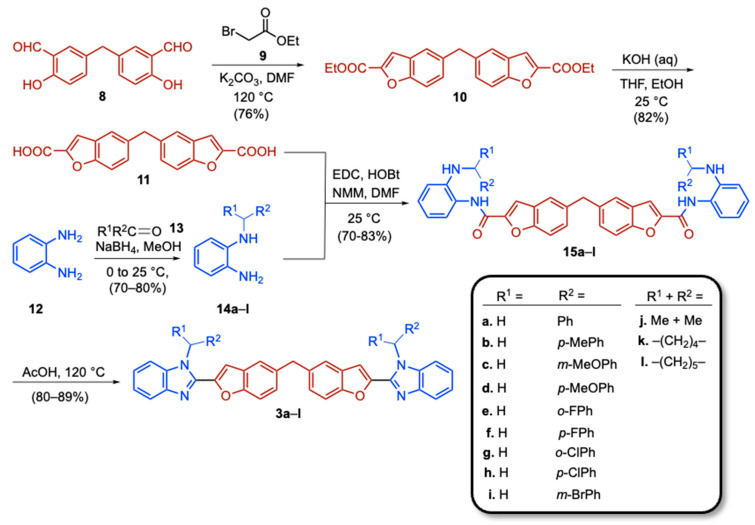
Synthesis of bis(benzofuran–benzimidazole)s **3**.

For establishment of the bis(benzofuran–benzimidazole) scaffold in conjugates **3**, an alternative synthetic approach was taken, as illustrated in Scheme 2. The bis-ester intermediate **10** was first prepared by coupling of bissalicyaldehyde **8** [60] with ethyl bromoacetate (**9**) in the presence of K_2_CO_3_ (s) and dry DMF at 120 °C [61]. This annulation reaction proceeded through a nucleophilic substitution, an intramolecular aldol condensation, and then a base-catalyzed dehydration. It gave bis(benzofuran-2-carboxylate) **10** with the desired skeleton as white crystals in 76% yield. In its ^1^H NMR spectrum, the peaks related to the CH_2_Me protons of the ester group appeared as a quartet at 4.41 ppm with *J*^3^ = 7.2 Hz and the peaks of the CCH_3_ proton appeared as a triplet at 1.39 ppm with *J*^3^ = 7.2 Hz. In its ^13^C NMR spectrum, the C=O carbon resonated at 159.4 ppm.

Saponification of bis-ester **10** was then performed by use of aqueous KOH in THF and ethanol at room temperature [62]. The desired benzofuroic acid dimer **11** was obtained in 82% yield as white solids. Then, two benzimidazole moieties were planned to build on the flanks of bis-benzofuranic acid **11**, as shown in Scheme 2. Meanwhile, the *N*-alkylated *o*-phenylenediamines **14a**–**l** were prepared from the reaction of *o*-phenylenediamine **12** with substituted aldehydes and ketones **13a**–**l**. This reductive amination was achieved in 70–80% yields by use of sodium borohydride in MeOH at 0–25 °C [63]. Subsequently, coupling of these diamines **14a**–**l** with benzofuran acid **11** with 1-ethyl-3-(3-dimethylaminopropyl)carbodiimide (EDC), hydroxybenzotriazole (HOBt), and *N*-methylmorpholine (NMM) in DMF led to the desired benzofuramide dimers **15a**–**l** in 70–83% yields. Identification of their structures is exemplified by compound **15j**. One characteristic singlet at *δ* 4.18 ppm in its ^1^H NMR spectrum showed the presence of the methylene linker (i.e., ArCH_2_Ar) in the dimer. A multiplet occurred at *δ* 3.57–3.51 ppm, which was related to the protons of the two CHMe_2_ groups. The peaks corresponding to the twelve protons from four methyl groups were observed at *δ* 1.22 ppm with *J*^2^ = 6.0 Hz. In its ^13^C NMR spectrum, the peak at *δ* 157.0 ppm was related to the two C=O carbons of amide groups; resonances that occurred at 45.4 and 22.9 ppm were due to the CMe_2_ and the CH_3_ carbons, respectively. A strong absorption band at 1660 cm^–1^ in its IR spectrum was attributed to the carbonyl stretching vibration in the amide groups.

Finally, the formation of benzimidazole moieties in the targets **3a**–**l** was achieved by use of acetic acid, which functioned as the catalyst and the solvent [51]. The diamides **15a**–**l** underwent cyclization followed by acid-catalyzed dehydration at 120 °C to give benzofuran–benzimdazoles **3a**–**l** in 80–89% yields (Scheme 2). We confirmed their structures using conjugate **3j** as an example. Its exact mass was measured as 565.2599 for (C_37_H_32_N_4_O_2_H)^+^, which is very close to its theoretical value of 565.2603. The ^1^H NMR spectrum showed a multiplet at *δ* 5.43–5.36 ppm and one singlet at *δ* 4.20 ppm, which corresponded to the two protons from two CHMe_2_ groups and two protons in the methylene linker of ArCH_2_Ar, respectively. The spectrum also exhibited a doublet at *δ* 1.71 ppm for twelve protons related to four methyl groups from two isopropyl groups. In its ^13^C NMR spectrum, resonance occurred at *δ* 143.6 ppm corresponding to the N=C−N carbon of the benzimidazole ring; resonances occurred at *δ* 49.1 and 21.5 ppm corresponding to the CMe_2_ and the CH_3_ carbons, respectively. There were 18 peaks, which are in agreement with the theoretical number.

### 2.3. Biological Activities of the New Bis-Conjugated Compounds and Their Lipophilicity

The anti-YFV activity of all new bis-conjugates and their intermediates was evaluated by use of cytopathic effect (CPE) reduction assays [10,64] on YFV strain 17D (Stamaril) in Huh-7 cells. Their biological data, including 50% effective concentration (EC_50_) and 50% cytotoxicity concentration (CC_50_), are summarized in Table 1. The EC_50_ value is the concentration of the test compound that inhibited the virus-induced cytopathic effect on the host cell by 50%. The CC_50_ value is the calculated concentration of the test compound required to reduce the metabolic activity of compound-treated cells by 50%. The selectivity index (SI = CC_50_/EC_50_) is a ratio between cytotoxicity and antiviral activity; it reflects the therapeutic window of the test compound in the assay system. Moreover, the molecular lipophilicity (quantified as log *p*) of bis-conjugates with promising potency against YFV was measured by use of the ‘‘shake–flask method’’ [65]. The lipophilicity of chemical compounds plays a vital role in the evaluation of drug candidates [66]. In general, the log *p* value between –0.4 and 5.6 is considered to be an ideal range for compounds as “drug-like” candidates [67]. 

Among these 23 new bis(benzofuran–1,3-*N*,*N*-heterocycle)s, compounds **2c**, **2e**, **2k**, and **3j** exhibited robust inhibitory activity against YFV with EC_50_ values of 3.54–8.89 μM (Table 1). In particular, two 1,3-imidazolidin-4-one conjugates (i.e., **2e** and **2k)** and one benzimidazole conjugate **3j** showed a favorable selectivity with SI values of 15.3, 8.43, and 11.3, respectively. The benzofuran intermediates **7**, **10**, and **11** without containing 1,3-*N*,*N*-heterocycle, however, did not exhibit any activity towards YFV. The log *p* values of conjugates **2c**, **2e**, **2k**, and **3j** with persuasive EC_50_ and SI values fell into the range of 3.71–5.16. Therefore, these bis-conjugates are advantageous as “drug-like” candidates on the basis of their lipophilicity.

### 2.4. Essential Moieties and Substituents for Anti-YFV Activity

Some of the new compounds, including **2c**, **2e**, **2k**, and **3j**, with five-membered 1,3-*N*,*N*-heterocycles (i.e., imidazolidinone and benzimidazole) attached to bisbenzofuran showed compelling EC_50_ values against YFV. Their log *p* values shown in Table 1 fell into an ideal range for their development as drug leads. The anti-YFV activity of 1,3-imidazolidinone **2e** had better efficacy than all other compounds in the same family and benzimidazoles **3**. 

The substituents attached to the nitrogen atoms were found of importance to determine the anti-YFV activity. Conjugates **2a**–**k** bearing the N-Ph moiety with a dimethyl or CF_3_ substituent were more potent than others in the series. When the substituent on the phenyl groups of conjugates was changed to monomethyl, isopropyl, methoxy, or a halogen substituent, the antiviral activity of conjugates **2** decreased or abolished completely. Among benzimidazoles **3a**–**l**, compound **3j** with an isopropyl substituent on the nitrogen atom was found to be the most active one in the series. When the substituents on the nitrogen atom of conjugates **3** was changed to cycloalkyl or a substituted phenyl group, their anti-YFV activity diminished (Table 1).

The three classes of conjugated compounds **1**–**3** share two common features. One is all of their infrastructures possessing two benzofuran moieties linked by a methylene unit. The other is that some compounds in all of these three classes possessed antiviral activities. Nevertheless, compounds **1** had a five-membered thiazolidinone ring, some of which exhibited activity against chikungunya virus [30]. When the sulfur atoms in the two five-membered heterocyclic rings were replaced by nitrogen atoms as shown in structures **2** and **3**, some showed cogent activity against YFV but not chikungunya virus.

## 3. Materials and Methods

### 3.1. General Information

All of the bis-conjugated compounds (i.e., **2a**–**k** and **3a**–**l**) were synthesized and their analytical data are presented below. The intermediates **6a**–**k**, **10**, **11,** **14a**–**l**, and **15a**–**l** were prepared according to the reported methods. Their synthesis and analytical data are provided in the Supplementary Materials. Commercially available solvents from Mallinckrodt Chemical Co., including dichloromethane, ethyl acetate, hexanes, and tetrahydrofuran (THF), were dried and distilled by the standard procedures. Other commercially available reagents were received and used without further purification. Compounds were purified by use of gravity column chromatography with Silicycle ultra-pure silica gel with particle sizes 40–63 μM and 230–400 mesh. Analytical thin layer chromatography (TLC) was performed on precoated plates with silica gel 60 F-254. Proton NMR spectra were recorded on 400 MHz and 700 MHz spectrometers by use of chloroform-*d* (CDCl_3_), dimethyl sulfoxide-*d*_6_ (DMSO-*d*_6_), and methanol-*d*_4_ (CD_3_OD) as the solvents. The chemical shifts were referenced to residual protonated solvents (*δ* 7.24 for chloroform, *δ* 2.49 for DMSO-*d*_6_, and *δ* 3.31 ppm for methanol). Carbon-13 NMR spectra were recorded on 100 MHz spectrometers by use of chloroform-*d* (CDCl_3_), dimethyl sulfoxide-d_6_ (DMSO-*d*_6_), and methanol-*d*_4_ (CD_3_OD) as the solvents. The chemical shifts are referenced to the center of the CDCl_3_ triplet (*δ* 77.0 ppm), DMSO-*d*_6_ septet (δ 39.5 ppm), and CD_3_OD septet (δ 49.0 ppm). Multiplicities are recorded by the following abbreviations: s, singlet; d, doublet; t, triplet; q, quartet; m, multiplet; and *J*, coupling constant (hertz). Infrared spectra (IR) were recorded on a Fourier transform infrared spectrometer (FT-IR). Absorption intensities are recorded by the following abbreviations: s, strong; m, medium; and w, weak. High-resolution mass spectra were measured on an electrospray ionization (ESI) or electron impact ionization (EI) mass spectrometer (MS).

### 3.2. Synthesis of Bis(Benzofuran–Imidazolidinone)s 2 (Standard Procedure 1)

To a solution of bisbenzofuranal **7 [30]** (1.0 equiv) in toluene (7.5–10 mL) were added *N*-arylamide **6** (2.0 equiv), Et_3_N (6.0–6.1 equiv), and powdered molecular sieves (0.100 g) at room temperature under nitrogen atmosphere. The reaction mixture was heated to reflux at 110 °C with stirring for 16–20 h. After being cooled to room temperature, the reaction mixture was concentrated under reduced pressure. The residue was diluted with EtOAc (50 mL), washed with H_2_O (2 × 20 mL) and brine (2 × 50 mL), dried over MgSO_4_ (s), filtered, and concentrated under reduced pressure. The residue was purified by use of silica gel column chromatography with a mixture of EtOAc and hexanes as the eluent to give the desired bis(benzofuran–imidazolidin-4-one)s **2**.

#### 3.2.1. (2″R,2‴R)-, (2″S,2‴S)-, and meso-(2″R,2‴S)-5,5′-Methylenebis[2-(N-methyl-3″-phenyl-4″-oxo-1″,3″-imidazolidin-2″-yl)benzofuran] (**2a**)

The standard procedure 1 was followed by use of bisbenzofuranal **7** (8.6 mg, 0.291 mmol, 1.0 equiv), *N*-arylamide **6a** (145 mg, 0.588 mmol, 2.0 equiv), Et_3_N (179 mg, 1.77 mmol, 6.1 equiv), powdered molecular sieves (0.100 g), and toluene (7.5 mL). After workup, the residue was purified by use of silica gel column chromatography (70% EtOAc in hexanes as the eluent) to give the desired bis(benzofuran–imidazolidinone) **2a** as a mixture of three isomers (135 mg, 0.226 mmol) in 78% overall yield as off-white solids: ^1^H NMR (CDCl_3_, 400 MHz) *δ* 7.35–7.33 (m, 6 H, 6 × ArH), 7.26–7.22 (m, 6 H, 6 × ArH), 7.12–7.08 (m, 4 H, 4 × ArH), 6.64 (s, 2 H, 2 × ArH), 5.60 (s, 2 H, 2 × NCHN), 4.05 (s, 2 H, ArCH_2_Ar), 3.82 (dd, *J* = 14.6, 0.8 Hz, 2 H, 2 × *H*CHN), 3.75 (d, *J* = 14.6 Hz, 2 H, 2 × HC*H*N), 2.44 (s, 6 H, 2 × NCH_3_); ^13^C NMR (CDCl_3_, 100 MHz) *δ* 170.4 (C=O), 153.9, 152.5, 136.3, 136.1, 128.8 (2 × C), 127.4, 126.0, 122.6, 121.0, 111.4, 107.4, 78.1 (NCHN), 56.9 (NCH_3_), 41.4 (ArCH_2_Ar), 39.1 (CH_2_N); IR (neat) 2798 (w), 1715 (s, C=O), 1598 (m), 1499 (m), 1384 (m), 1262 (w), 1037 (w), 757 (w) cm^–1^; ESIMS calcd for (C_37_H_32_N_4_O_4_): 596.2424; found 596.2427.

#### 3.2.2. (2″R,2‴R)-, (2″S,2‴S)-, and meso-(2″R,2‴S)-5,5′-Methylenebis(2-[N-methyl-3″-(4-methylphenyl)-4″-oxo-1″,3″-imidazolidin-2″-yl]benzofuran) (**2b**)

The standard procedure 1 was followed by use of bisbenzofuranal **7** (77.7 mg, 0.255 mmol, 1.0 equiv), *N-*arylamide **6b** (149 mg, 0.511 mmol, 2.0 equiv), Et_3_N (155 mg, 1.53 mmol, 6.0 equiv), powdered molecular sieves (0.100 g), and toluene (7.5 mL). After workup, the residue was purified by use of silica gel column chromatography (75% EtOAc in hexanes as the eluent) to give the desired bis(benzofuran–imidazolidinone) **2b** as a mixture of three isomers (120 mg, 0.192 mmol) in 76% overall yield as off-white solids: ^1^H NMR (CDCl_3_, 400 MHz) *δ* 7.34 (d, *J* = 8.8 Hz, 2 H, 2 × ArH), 7.26 (s, 2 H, 2 × ArH), 7.19 (d, *J* = 8.0 Hz, 4 H, 4 × ArH), 7.10 (d, *J* = 8.0 Hz, 2 H, 2 × ArH), 7.04 (d, *J* = 8.0 Hz, 4 H, 4 × ArH), 6.63 (s, 2 H, 2 × ArH), 5.55 (s, 2 H, 2 × NCHN), 4.05 (s, 2 H, ArCH_2_Ar), 3.82 (d, *J* = 14.8 Hz, 2 H, 2 × *H*CHN), 3.42 (d, *J* = 14.8 Hz, 2 H, 2 × HC*H*N), 2.43 (s, 6 H, 2 × NCH_3_), 2.22 (s, 6 H, 2 × ArCH_3_); ^13^C NMR (CDCl_3_, 100 MHz) *δ* 170.4 (C=O), 153.9, 152.7, 136.3, 136.0, 133.5, 129.5, 127.5, 126.0, 123.0, 121.0, 111.5, 107.5, 78.3 (NCHN), 56.9 (NCH_3_), 41.4 (ArCH_2_Ar), 39.1 (CH_2_N), 20.8 (ArCH_3_); IR (neat) 2798 (w), 1715 (s, C=O), 1598 (w), 1500 (m), 1384 (m), 1261 (w), 1037 (w), 757 (w) cm^–1^; ESIMS calcd for (C_39_H_36_N_4_O_4_): 629.2737; found 629.2740.

#### 3.2.3. (2″R,2‴R)-, (2″S,2‴S)-, and meso-(2″R,2‴S)-5,5′-Methylenebis(2-[N-methyl-3″-(2,3-dimethylphenyl)-4″-oxo-1″,3″-imidazolidin-2″-yl]benzofuran) (**2c**)

The standard procedure 1 was followed by use of bisbenzofuranal **7** (47.2 mg, 0.155 mmol, 1.0 equiv), *N-*arylamide **6c** (99.7 mg, 0.325 mmol, 2.1 equiv), Et_3_N (94.1 mg, 0.931 mmol, 6.0 equiv), powdered molecular sieves (0.100 g), and toluene (7.5 mL). After workup, the residue was purified by use of silica gel column chromatography (68% EtOAc in hexanes as the eluent) to give the desired bis(benzofuran–imidazolidinone) **2c** as a mixture of three isomers (0.100 g, 0.131 mmol) in 85% overall yield as pale yellow solids: ^1^H NMR (CDCl_3_, 400 MHz) *δ* 7.38 (d, *J* = 8.0 Hz, 2 H, 2 × ArH), 7.27 (s, 2 H, 2 × ArH), 7.14 (d, *J* = 8.0 Hz, 4 H, 4 × ArH), 7.01 (d, *J* = 7.2 Hz, 2 H, 2 × ArH), 7.02–6.94 (m, 2 H, 2 × ArH), 6.61 (s, 2 H, 2 × ArH), 5.36 (s, 2 H, 2 × NCHN), 4.07 (s, 2 H, ArCH_2_Ar), 3.65 (d, *J* = 13.6 Hz, 2 H, 2 × *H*CHN), 3.50 (d, *J* = 13.6 Hz, 2 H, 2 × HC*H*N), 2.52 (s, 6 H, 2 × NCH_3_), 2.29 (s, 6 H, 2 × ArCH_3_), 2.14 (s, 6 H, 2 × ArCH_3_); ^13^C NMR (CDCl_3_, 100 MHz) *δ* 170.4 (C=O), 154.0, 152.5, 138.2, 136.2 (2 × C), 133.7, 129.9, 127.4, 126.1, 125.9 (2 × C), 121.0, 111.5, 108.4, 79.1 (NCHN), 56.8 (NCH_3_), 41.5 (ArCH_2_Ar), 38.7 (CH_2_N), 20.3 (ArCH_3_), 14.2 (ArCH_3_); IR (neat) 2924 (m), 1715 (s, C=O), 1618 (w), 1508 (m), 1379 (m), 1261 (s), 1038 (w), 791 (w) cm^–1^; ESIMS calcd for (C_41_H_40_N_4_O_4_): 652.3050; found 652.3056.

#### 3.2.4. (2″R,2‴R)-, (2″S,2‴S)-, and meso-(2″R,2‴S)-5,5′-Methylenebis(2-[N-methyl-3″-(2,5-dimethylphenyl)-4″-oxo-1″,3″-imidazolidin-2″-yl]benzofuran) (**2d**)

The standard procedure 1 was followed by use of bisbenzofuranal **7** (45.1 mg, 0.147 mmol, 1.0 equiv), *N-*arylamide **6d** (95.0 mg, 0.310 mmol, 2.1 equiv), Et_3_N (89.2 mg, 0.882 mmol, 6.0 equiv), powdered molecular sieves (0.100 g), and toluene (7.5 mL). After workup, the residue was purified by use of silica gel column chromatography (70% EtOAc in hexanes as the eluent) to give the desired bis(benzofuran–imidazolidinone) **2d** as a mixture of three isomers (80.1mg, 0.123 mmol) in 83% overall yield as pale yellow solids: ^1^H NMR (CDCl_3_, 400 MHz) *δ* 7.38 (d, *J* = 8.0 Hz, 2 H, 2 × ArH), 7.27 (s, 2 H, 2 × ArH), 7.11 (d, *J* = 1.6 Hz, 2 H, 2 × ArH), 7.02 (d, *J* = 8.0 Hz, 2 H, 2 × ArH), 6.93 (d, *J* = 1.6 Hz, 2 H, 2 × ArH), 6.78 (s, 2 H, 2 × ArH), 6.63 (s, 2 H, 2 × ArH), 5.38 (s, 2 H, 2 × NCHN), 4.07 (s, 2 H, ArCH_2_Ar), 3.94 (dd, *J* = 14.2, 1.4 Hz, 2 H, 2 × *H*CHN), 3.46 (dd, *J* = 14.2, 1.4 Hz, 2 H, 2 × HC*H*N), 2.46 (s, 6 H, 2 × NCH_3_), 2.20 (s, 6 H, 2 × ArCH_3_), 2.14 (s, 6 H, 2 × ArCH_3_); ^13^C NMR (CDCl_3_, 100 MHz) *δ* 170.1 (C=O), 153.9, 152.3, 136.1 (2 × C), 133.5, 133.2, 130.8, 129.1, 127.4, 127.3, 126.1, 121.0, 111.3, 108.3, 79.0 (NCHN), 56.7 (NCH_3_), 41.4 (ArCH_2_Ar), 38.8 (CH_2_N), 20.6 (ArCH_3_), 17.3 (ArCH_3_); IR (neat) 2923 (m), 1715 (s, C=O), 1509 (m), 1431 (m), 1380 (m), 1261 (m), 1038 (w), 736 (w) cm^–1^; ESIMS calcd for (C_41_H_40_N_4_O_4_): 652.3050; found 652.3055.

#### 3.2.5. (2″R,2‴R)-, (2″S,2‴S)-, and meso-(2″R,2‴S)-5,5′-Methylenebis[2-(N-methyl-3″-(3,5-dimethylphenyl)-4″-oxo-1″,3″-imidazolidin-2″-yl)benzofuran] (**2e**)

The standard procedure 1 was followed by use of bisbenzofuranal **7** (42.5 mg, 0.139 mmol, 1.0 equiv), *N-*arylamide **6e** (89.7 mg, 0.293 mmol, 2.1 equiv), Et_3_N (84.3 mg, 0.834 mmol, 6.0 equiv), powdered molecular sieves (0.100 g), and toluene (7.5 mL). After workup, the residue was purified by use of silica gel column chromatography (75% EtOAc in hexanes as the eluent) to give the desired bis(benzofuran–imidazolidinone) **2e** as a mixture of three isomers (72.9 mg, 0.111 mmol) in 80% overall yield as pale yellow solids: ^1^H NMR (CDCl_3_, 400 MHz) *δ* 7.36 (d, *J* = 8.4 Hz, 2 H, 2 × ArH), 7.28 (s, 2 H, 2 × ArH), 7.11 (d, *J* = 8.4 Hz, 2 H, 2 × ArH), 6.99 (s, 4 H, 4 × ArH), 6.75 (s, 2 H, 2 × ArH), 6.65 (s, 2 H, 2 × ArH), 5.60 (s, 2 H, 2 × NCHN), 4.06 (s, 2 H, ArCH_2_Ar), 3.82 (d, *J* = 15.0 Hz, 2 H, 2 × *H*CHN), 3.42 (d, *J* = 15.0 Hz, 2 H, 2 × HC*H*N), 2.44 (s, 6 H, 2 × NCH_3_), 2.20 (s, 12 H, 4 × CH_3_); ^13^C NMR (CDCl_3_, 100 MHz) *δ* 170.3 (C=O), 153.8, 152.6, 138.4, 136.2, 135.8, 127.9, 127.5, 125.9, 121.0, 120.6, 111.3, 107.2, 78.2 (NCHN), 56.9 (NCH_3_), 41.3 (ArCH_2_Ar), 39.2 (CH_2_N), 21.2 (ArCH_3_); IR (neat) 2924 (w), 1714 (s, C=O), 1508 (m), 1444 (m), 1379 (m), 1260 (m), 1038 (w), 736 (m) cm^–1^; ESIMS calcd for (C_41_H_40_N_4_O_4_): 652.3050; found 652.3052. 

#### 3.2.6. (2″R,2‴R)-, (2″S,2‴S)-, and meso-(2″R,2‴S)-5,5′-Methylenebis(2-[N-methyl-3″-(2-ethylphenyl)-4″-oxo-1″,3″-imidazolidin-2″-yl]benzofuran) (**2f**)

The standard procedure 1 was followed by use of bisbenzofuranal **7** (33.1 mg, 0.108 mmol, 1.0 equiv), *N-*arylamide **6f** (69.2 mg, 0.228 mmol, 2.1 equiv), Et_3_N (65.6 mg, 0.648 mmol, 6.0 equiv), powdered molecular sieves (0.100 g), and toluene (8.5 mL). After workup, the residue was purified by use of silica gel column chromatography (85% EtOAc in hexanes as the eluent) to give the desired bis(benzofuran–imidazolidinone) **2f** as a mixture of three isomers (57.4 mg, 87.2 µmol) in 82% overall yield as pale yellow solids: ^1^H NMR (CDCl_3_, 400 MHz) *δ* 7.36 (d, *J* = 8.4 Hz, 2 H, 2 × ArH), 7.25–7.24 (m, 2 H, 2 × ArH), 7.22–7.16 (m, 4 H, 4 × ArH), 7.13 (d, *J* = 8.4 Hz, 2 H, 2 × ArH), 7.03 (t, *J* = 7.2 Hz, 2 H, 2 × ArH), 7.01–6.99 (m, 2 H, 2 × ArH), 6.60 (s, 2 H, 2 × ArH), 5.35 (s, 2 H, 2 × NCHN), 4.05 (s, 2 H, ArCH_2_Ar), 3.92 (d, *J* = 14.4 Hz, 2 H, 2 × *H*CHN), 3.48 (d, *J* = 14.4 Hz, 2 H, 2 × HC*H*N), 2.43 (s, 6 H, 2 × NCH_3_), 2.62 (q, *J* = 7.6 Hz, 4 H, 2 × ArC*H*_2_CH_3_), 1.16 (t, *J* = 7.6 Hz, 6 H, 2 × ArCH_2_C*H*_3_); ^13^C NMR (CDCl_3_, 100 MHz) *δ* 170.6 (C=O), 154.0, 152.3, 142.3, 136.2 (2 × C), 133.1, 128.9, 128.6, 127.3, 126.5, 126.2, 121.0, 111.4, 108.6, 79.2 (NCHN), 56.7 (NCH_3_), 41.4 (ArCH_2_Ar), 38.7 (CH_2_N), 23.6 (Ar*C*H_2_CH_3_), 14.2 (ArCH_2_*C*H_3_); IR (neat) 2966 (w), 1716 (s, C=O), 1454 (m), 1395 (m), 1290 (w), 1257 (m), 1038 (w), 734 (w) cm^–1^; ESIMS calcd for (C_41_H_40_N_4_O_4_ + H): 653.3128; found 653.3128.

#### 3.2.7. (2″R,2‴R)-, (2″S,2‴S)-, and meso-(2″R,2‴S)-5,5′-Methylenebis(2-[N-methyl-3″-(4-methoxyphenyl)-4″-oxo-1″,3″-imidazolidin-2″-yl]benzofuran) (**2g**)

The standard procedure 1 was followed by use of bisbenzofuranal **7** (85.2 mg, 0.280 mmol, 1.0 equiv), *N-*arylamide **6g** (179 mg, 0.588 mmol, 2.1 equiv), Et_3_N (170 mg, 1.68 mmol, 6.0 equiv), powdered molecular sieves (0.100 g), and toluene (6.5 mL). After workup, the residue was purified by use of silica gel column chromatography (75% EtOAc in hexanes as the eluent) to give the desired bis(benzofuran–imidazolidinone) **2g** as a mixture of three isomers (144 mg, 0.220 mmol) in 79% overall yield as pale yellow solids: ^1^H NMR (CDCl_3_, 400 MHz) *δ* 7.35 (d, *J* = 8.4 Hz, 2 H, 2 × ArH), 7.27–7.24 (m, 2 H, 2 × ArH), 7.19–7.09 (m, 6 H, 6 × ArH), 6.60 (d, *J* = 9.2 Hz, 4 H, 4 × ArH), 6.63 (s, 2 H, 2 × ArH), 5.47 (s, 2 H, 2 × NCHN), 4.06 (s, 2 H, ArCH_2_Ar), 3.84 (dd, *J* = 14.6, 1.2 Hz, 2 H, 2 × *H*CHN), 3.69 (s, 6 H, 2 × OCH_3_), 3.43 (dd, *J* = 14.6, 1.2 Hz, 2 H, 2 × HC*H*N), 2.43 (s, 6H, 2 × NCH_3_); ^13^C NMR (CDCl_3_, 100 MHz) *δ* 170.5 (C=O), 157.9, 154.0, 152.7, 136.3, 128.7, 127.5, 126.1, 125.4, 121.1, 114.3, 111.5, 107.8, 78.7 (NCHN), 56.9 (NCH_3_), 55.3 (OCH_3_), 41.5 (ArCH_2_Ar), 39.1 (CH_2_N); IR (neat) 2798 (m), 1718 (s, C=O), 1597 (w), 1491 (m), 1381 (m), 1262 (w), 1074 (w), 736 (w) cm^–1^; ESIMS calcd for (C_39_H_36_N_4_O_6_): 656.2635; found 656.2633.

#### 3.2.8. (2″R,2‴R)-, (2″S,2‴S)-, and meso-(2″R,2‴S)-5,5′-Methylenebi(2-[N-methyl-3″-(4-fluorophenyl)-4″-oxo-1″,3″-imidazolidin-2″-yl]benzofuran) (**2h**)

The standard procedure 1 was followed by use of bisbenzofuranal **7** (55.1 mg, 0.124 mmol, 1.0 equiv), *N-*arylamide **6h** (112 mg, 0.382 mmol, 2.1 equiv), Et_3_N (110 mg, 1.09 mmol, 6.0 equiv), powdered molecular sieves (0.100 g), and toluene (7.5 mL). After workup, the residue was purified by use of silica gel column chromatography (75% EtOAc in hexanes as the eluent) to give the desired bis(benzofuran–imidazolidinone) **2h** as a mixture of three isomers (91.2 mg, 0.144 mmol) in 80% overall yield as pale yellow solids: ^1^H NMR (CDCl_3_, 400 MHz) *δ* 7.36 (d, *J* = 8.4 Hz, 2 H, 2 × ArH), 7.35–7.27 (m, 6 H, 6 × ArH), 7.15 (d, *J* = 1.6 Hz, 2 H, 2 × ArH), 6.97–6.92 (m, 4 H, 4 × ArH), 6.68 (s, 2 H, 2 × ArH), 5.54 (s, 2 H, 2 × NCHN), 4.08 (s, 2 H, ArCH_2_Ar), 3.85 (dd, *J* = 14.4, 0.8 Hz, 2 H, 2 × *H*CHN), 3.45 (dd, *J* = 14.4, 0.8 Hz, 2 H, 2 × HC*H*N), 2.45 (s, 6 H, 2 × NCH_3_); ^13^C NMR (CDCl_3_, 100 MHz) *δ* 170.4 (C=O), 160.5 (d, *J_CF_* = 245 Hz), 153.9, 152.2, 136.4, 131.9, 127.4, 126.2, 125.1 (d, *J_CF_* = 8.4 Hz), 121.1, 115.8 (d, *J_CF_* = 23 Hz), 111.5, 107.9, 78.3 (NCHN), 56.8 (NCH_3_), 41.4 (ArCH_2_Ar), 38.9 (CH_2_N); IR (neat) 2798 (s), 1716 (s, C=O), 1585 (m), 1489 (m), 1376 (m), 1194 (w), 1055 (w), 736 (w) cm^–1^; ESIMS calcd for (C_37_H_30_ F_2_N_2_O_4_): 632.2235; found 632.2240.

#### 3.2.9. (2″R,2‴R)-, (2″S,2‴S)-, and meso-(2″R,2‴S)-5,5′-Methylenebis(2-[N-methyl-3″-(4-chlorophenyl)-4″-oxo-1″,3″-imidazolidin-2″-yl]benzofuran) (**2i**)

The standard procedure 1 was followed by use of bisbenzofuranal **7** (51.1 mg, 0.167 mmol, 1.0 equiv), *N-*arylamide **6i** (109 mg, 0.352 mmol, 2.1 equiv), Et_3_N (101 mg, 1.00 mmol, 6.0 equiv), powdered molecular sieves (0.100 g), and toluene (7.5 mL). After workup, the residue was purified by use of silica gel column chromatography (75% EtOAc in hexanes as the eluent) to give the desired bis(benzofuran–imidazolidinone) **2i** as a mixture of three isomers (86.8 mg, 0.130 mmol) in 78% overall yield as pale yellow solids: ^1^H NMR (CDCl_3_, 400 MHz) *δ* 7.37–7.34 (m, 4 H, 4 × ArH), 7.33–7.30 (m, 4 H, 4× ArH), 7.23–7.21 (m, 4 H, 4 × ArH), 7.14 (dd, *J* = 8.6, 1.6 Hz, 2 H, 2 × ArH), 6.68 (s, 2 H, 2 × ArH), 5.59 (s, 2 H, 2 × NCHN), 4.08 (s, 2 H, ArCH_2_Ar), 3.84 (d, *J* = 15.0 Hz, 2 H, 2 × *H*CHN), 3.45 (d, *J* = 15.0 Hz, 2 H, 2 × HC*H*N), 2.45 (s, 6 H, 2 × NCH_3_); ^13^C NMR (CDCl_3_, 100 MHz) *δ* 170.3 (C=O), 153.9, 152.1, 136.4, 134.7, 131.3, 129.0, 127.4, 126.2, 123.8, 121.1, 111.5, 107.7, 77.9 (NCHN), 56.8 (NCH_3_), 41.4 (ArCH_2_Ar), 39.0 (CH_2_N); IR (neat) 2798 (w), 1718 (s, C=O), 1597 (w), 1491 (s), 1444 (w), 1381 (s), 1120 (w), 803 (w) cm^–1^; ESIMS calcd for (C_37_H_30_ Cl_2_N_4_O_4_): 664.1644; found 664.1645.

#### 3.2.10. (2″R,2‴R)-, (2″S,2‴S)-, and meso-(2″R,2‴S)-5,5′-Methylenebis(2-[N-methyl-3″-(4-bromophenyl)-4″-oxo-1″,3″-imidazolidin-2″-yl]benzofuran) (**2j**)

The standard procedure 1 was followed by use of bisbenzofuranal **7** (47.1 mg, 0.155 mmol, 1.0 equiv), *N-*arylamide **6j** (116 mg, 0.327 mmol, 2.1 equiv), Et_3_N (94.2 mg, 0.930 mmol, 6.0 equiv), powdered molecular sieves (0.100 g), and toluene (6.8 mL). After workup, the residue was purified by use of silica gel column chromatography (70% EtOAc in hexanes as the eluent) to give the desired bis(benzofuran–imidazolidinone) **2j** as a mixture of three isomers (87.3 mg, 0.116 mmol) in 78% overall yield as pale yellow solids: ^1^H NMR (CDCl_3_, 400 MHz) *δ* 7.36–7.32 (m, 6 H, 6 × ArH), 7.27–7.24 (m, 6 H, 6 × ArH), 7.11 (dd, *J* = 8.4, 1.6 Hz, 2 H, 2 × ArH), 6.65 (s, 2 H, 2 × ArH), 5.56 (s, 2 H, 2 × NCHN), 4.06 (s, 2 H, ArCH_2_Ar), 3.81 (d, *J* = 15.0 Hz, 2 H, 2 × *H*CHN), 3.42 (d, *J* = 15.0 Hz, 2 H, 2 × HC*H*N), 2.43 (s, 6 H, 2 × NCH_3_); ^13^C NMR (CDCl_3_, 100 MHz) *δ* 170.3 (C=O), 153.9, 152.1, 136.4, 135.2, 131.9, 127.3, 126.2, 123.9, 121.1, 119.1, 111.5, 107.7, 77.8 (NCHN), 56.8 (NCH_3_), 41.4 (ArCH_2_Ar), 39.0 (CH_2_N); IR (neat) 2948 (w), 1718 (s, C=O), 1597 (m), 1491 (s), 1381 (s), 1262 (w), 1074 (w), 736 (w) cm^–1^; ESIMS calcd for (C_37_H_30_ Br_2_N_4_O_4_): 752.0634; found 752.0631.

#### 3.2.11. (2″R,2‴R)-, (2″S,2‴S)-, and meso-(2″R,2‴S)-5,5′-Methylenebis(2-[N-methyl-3″-(3-trifluoromethyl)phenyl-4″-oxo-1″,3″-imidazolidin-2″-yl]benzofuran) (**2k**)

The standard procedure 1 was followed by use of bisbenzofuranal **7** (33.0 mg, 0.108 mmol, 1.0 equiv), *N-*arylamide **6k** (78.8 mg, 0.227 mmol, 2.1 equiv), Et_3_N (61.6 mg, 0.648 mmol, 6.0 equiv), powdered molecular sieves (0.100 g), and toluene (7.0 mL). After workup, the residue was purified by use of silica gel column chromatography (82% EtOAc in hexanes as the eluent) to give the desired bis(benzofuran–imidazolidinone) **2k** as a mixture of three isomers (67.5 mg, 92.1 µmol) in 85% overall yield as pale yellow solids: ^1^H NMR (CDCl_3_, 400 MHz) *δ* 7.73 (s, 2 H, 2 × ArH), 7.55 (d, *J* = 6.4 Hz, 2 H, 2 × ArH), 7.36–7.28 (m, 6 H, 6 × ArH), 7.28 (s, 2 H, 2 × ArH), 7.11 (d, *J* = 8.4 Hz, 2 H, 2 × ArH), 6.70 (s, 2 H, 2 × ArH), 5.65 (s, 2 H, 2 × NCHN), 4.06 (s, 2 H, ArCH_2_Ar), 3.84 (dd, *J* = 15.0, 1.2 Hz, 2 H, 2 × *H*CHN), 3.45 (d, *J* = 15.0 Hz, 2 H, 2 × HC*H*N), 2.45 (s, 6 H, 2 × NCH_3_); ^13^C NMR (CDCl_3_, 100 MHz) *δ* 170.4 (C=O), 153.9, 151.9, 136.1, 136.4, 131.2 (q, *J_CF_* = 32.4 Hz), 129.4, 127.3, 125.3, 123.5 (q, *J_CF_* = 272 Hz), 122.4 (q, *J_CF_* = 3.6 Hz), 122.4, 118.9, 118.9 (d, *J_CF_* = 3.6 Hz), 111.5, 107.8, 77.7 (NCHN), 56.8 (NCH_3_), 41.4 (ArCH_2_Ar), 39.0 (CH_2_N); IR (neat) 2951 (w), 1722 (s, C=O), 1497 (m), 1458 (m), 1382 (s), 1329 (s), 1126 (s), 798 (w) cm^–1^; ESIMS calcd for (C_39_H_30_ F_6_N_4_O_4_ + H): 733.2249; found 733.2246.

### 3.3. Synthesis of Bis(Benzofuran–Benzimidazole)s 3 (Standard Procedure 2)

The solution of diamides **15** (1.0 equiv) in AcOH (2.5–5.0 mL) was heated to 120 °C for 4.0 h under N_2_ atmosphere. The cooled reaction mixture was neutralized with cold saturated aqueous NaHCO_3_ solution (10–20 mL), which was extracted with EtOAc (3 × 30 mL). The combined organic extracts were washed with water (2 × 50 mL) and then brine (2 × 50 mL), dried over MgSO_4_ (s), filtered, and concentrated under reduced pressure. The residue was purified by use of silica gel column chromatography with a mixture of EtOAc and hexanes as the eluent to give bis(benzofuran–benzimidazole)s **3**.

#### 3.3.1. 5,5′-Methylenebis[2-(1-benzylbenzimidazol-2-yl)benzofuran] (**3a**)

The standard procedure 2 was followed by use of diamide **15a** (55.1 mg, 78.8 µmol, 1.0 equiv) in AcOH (2.5 mL). After workup, the residue was purified by use of silica gel column chromatography with 30% EtOAc in hexanes as the eluent to give the desired bis(benzofuran–benzimidazole) **3a** (42.5 mg, 64.2 µmol) in 82% overall yield as pale yellow solids: mp (recrystallized from hexanes/CH_2_Cl_2_) 183.6–185.2 °C; ^1^H NMR (CDCl_3_, 400 MHz) *δ* 7.85 (d, *J* = 8.0 Hz, 2 H, 2 × ArH), 7.43 (d, *J* = 8.8 Hz, 4 H, 4 × ArH), 7.36–7.30 (m, 6 H, 6 × ArH), 7.27–7.24 (m, 8 H, 8 × ArH), 7.18 (d, *J* = 8.8 Hz, 2 H, 2 × ArH), 7.15 (d, *J* = 8.0 Hz, 4 H, 4 × ArH), 5.76 (s, 4 H, 2 × CH_2_Ph), 4.13 (s, 2 H, ArCH_2_Ar); ^13^C NMR (CDCl_3_, 100 MHz) *δ* 153.9, 146.8, 144.1, 143.2, 136.9, 136.2, 136.0, 128.9, 128.0, 127.8, 127.1 126.2, 123.8, 123.2, 121.6, 120.2, 111.6, 111.1, 108.6, 48.5 (CH_2_Ph), 41.6 (ArCH_2_Ar); IR (neat) 2924 (m), 2855 (m), 1446 (s), 1387 (m), 1332 (m), 1194 (m), 1121 (w), 739 (m) cm^–1^; ESIMS calcd for (C_45_H_32_N_4_O_2_): 660.2525; found 660.2523.

#### 3.3.2. 5,5′-Methylenebis(2-[1-(p-methylbenzyl)benzimidazol-2-yl]benzofuran) (**3b**)

The standard procedure 2 was followed by use of diamide **15b** (80.4 mg, 0.111 mmol, 1.0 equiv) in AcOH (3.5 mL). After workup, the residue was purified by use of silica gel column chromatography (30% EtOAc in hexanes as the eluent) to give the desired bis(benzofuran–benzimidazole) **3b** (64.2 mg, 93.5 µmol) in 84% overall yield as light yellow solids: mp (recrystallized from hexanes/CH_2_Cl_2_) 188.2–189.8 °C; ^1^H NMR (CDCl_3_, 400 MHz) *δ* 7.86 (d, *J* = 8.0 Hz, 2 H, 2 × ArH), 7.45 (d, *J* = 8.8 Hz, 2 H, 2 × ArH), 7.42 (s, 2 H, 2 × ArH), 7.32–7.18 (m, 8 H, 8 × ArH), 7.19 (d, *J* = 8.8 Hz, 2 H, 2 × ArH), 7.08–7.03 (m, 8 H, 8 × ArH), 5.71 (s, 4 H, 2 × CH_2_Ph), 4.13 (s, 2 H, ArCH_2_Ar), 2.27 (s, 6 H, 2 × CH_3_); ^13^C NMR (CDCl_3_, 100 MHz) *δ* 153.9, 146.8, 144.1, 143.2, 137.5, 136.8, 136.2, 136.0, 129.6, 128.1, 127.0, 126.2, 123.7, 123.1, 121.6, 120.1, 111.5, 110.1, 108.5, 48.3 (CH_2_Ph), 41.5 (ArCH_2_Ar), 21.0 (CH_3_); IR (neat) 2923 (m), 2854 (m), 1515 (w), 1445 (s), 1387 (m), 1332 (m), 1194 (m), 920 (w), 739 (m) cm^–1^; ESIMS calcd for (C_47_H_36_N_4_O_2_ + H): 689.2917; found 689.2918.

#### 3.3.3. 5,5′-Methylenebis(2-[1-(m-methoxybenzyl)benzimidazol-2-yl]benzofuran) (**3c**)

The standard procedure 2 was followed by use of diamide **15c** (65.1 mg, 86.1 µmol, 1.0 equiv) in AcOH (3.0 mL). After workup, the residue was purified by use of silica gel column chromatography (35% EtOAc in hexanes as the eluent) to give the desired bis(benzofuran–benzimidazole) **3c** (49.5 mg, 68.8 µmol) in 80% overall yield as white solids: mp (recrystallized from hexanes/CH_2_Cl_2_) 175.4–177.2 °C; ^1^H NMR (CDCl_3_, 400 MHz) *δ* 7.85 (d, *J* = 8.8 Hz, 2 H, 2 × ArH), 7.43 (d, *J* = 8.8 Hz, 2 H, 2 × ArH), 7.41 (s, 2 H, 2 × ArH), 7.31–7.25 (m, 8 H, 8 × ArH), 7.20–7.16 (m, 4 H, 4 × ArH), 6.76 (dd, *J* = 7.8, 2.8 Hz, 2 H, 2 × ArH), 6.71 (d, *J* = 7.8 Hz, 2 H, 2 × ArH), 6.68 (s, 2 H, 2 × ArH), 5.70 (s, 4 H, 2 × CH_2_Ph), 4.12 (s, 2 H, ArCH_2_Ar), 3.36 (s, 6 H, 2 × OCH_3_); ^13^C NMR (CDCl_3_, 100 MHz) *δ* 160.0, 153.9, 146.7, 144.1, 143.1, 137.8, 136.8, 136.0, 130.0, 128.1, 127.1, 123.8, 123.2, 121.6, 120.1, 118.4, 112.8, 112.2, 111.5, 110.1, 108.6, 55.1 (OCH_3_), 48.4 (CH_2_Ph), 41.5 (ArCH_2_Ar); IR (neat) 2938 (m), 2836 (w), 1601 (m), 1586 (s), 1446 (s), 1387 (m), 1263 (s), 1195 (m), 925 (w), 742 (m) cm^–1^; ESIMS calcd for (C_47_H_36_N_4_O_4_ + H): 721.2814; found 721.2812.

#### 3.3.4. 5,5′-Methylenebis(2-[1-(p-methoxybenzyl)benzimidazol-2-yl]benzofuran) (**3d**)

The standard procedure 2 was followed by use of diamide **15d** (78.4 mg, 0.103 mmol, 1.0 equiv) in AcOH (4.0 mL). After workup, the residue was purified by use of silica gel column chromatography (35% EtOAc in hexanes as the eluent) to give the desired bis(benzofuran–benzimidazole) **3d** (62.6 mg, 87.1 µmol) in 86% overall yield as white solids: mp (recrystallized from hexanes/CH_2_Cl_2_) 184.8–186.2 °C; ^1^H NMR (CDCl_3_, 400 MHz) *δ* 7.85 (d, *J* = 7.6 Hz, 2 H, 2 × ArH), 7.45 (d, *J* = 8.6 Hz, 2 H, 2 × ArH), 7.42 (s, 2 H, 2 × ArH), 7.32–7.23 (m, 8 H, 8 × ArH), 7.19 (d, *J* = 8.6 Hz, 2 H, 2 × ArH), 7.07 (d, *J* = 7.8 Hz, 4 H, 4 × ArH), 6.78 (d, *J* = 7.8 Hz, 4 H, 4 × ArH), 5.66 (s, 4 H, 2 × CH_2_Ph), 4.12 (s, 2 H, ArCH_2_Ar), 3.70 (s, 6 H, 2 × OCH_3_); ^13^C NMR (CDCl_3_, 100 MHz) *δ* 159.0, 153.9, 146.8, 143.9, 143.1, 136.8, 135.9, 128.1, 128.0, 127.5, 127.0, 123.6, 123.1, 121.5, 120.1, 114.2, 111.5, 110.1, 108.5, 55.1 (OCH_3_), 47.9 (CH_2_Ph), 41.5 (ArCH_2_Ar); IR (neat) 3052 (m), 2938 (m), 2836 (w), 1586 (s), 1455 (s), 1332 (m), 1287 (s), 1195 (m), 925 (w), 742 (m) cm^–1^; ESIMS calcd for (C_47_H_36_N_4_O_4_ + H): 721.2814; found 721.2811.

#### 3.3.5. 5,5′-Methylenebis(2-[1-(o-fluorobenzyl)benzimidazol-2-yl]benzofuran) (**3e**)

The standard procedure 2 was followed by use of diamide **15e** (82.4 mg, 0.108 mmol, 1.0 equiv) in AcOH (5.0 mL). After workup, the residue was purified by use of silica gel column chromatography (30% EtOAc in hexanes as the eluent) to give the desired bis(benzofuran–benzimidazole) **3e** (60.5 mg, 86.9 µmol) in 80% overall yield as pale yellow solids: mp (recrystallized from hexanes/CH_2_Cl_2_) 188.6–189.4 °C; ^1^H NMR (CDCl_3_, 400 MHz) *δ* 7.86 (d, *J* = 7.2 Hz, 2 H, 2 × ArH), 7.42 (d, *J* = 8.6 Hz, 4 H, 4 × ArH), 7.35–7.24 (m, 8 H, 8 × ArH), 7.20–7.10 (m, 6 H, 6 × ArH), 6.91 (t, *J* = 8.6 Hz, 4 H, 4 × ArH), 5.83 (s, 4 H, 2 × CH_2_Ph), 4.13 (s, 2 H, ArCH_2_Ar); ^13^C NMR (CDCl_3_, 100 MHz) *δ* 159.9 (d, *J_CF_* = 245 Hz), 158.6, 153.9, 146.7, 144.1, 143.1, 136.8, 135.9, 129.5 (d, *J_CF_* = 7.5 Hz), 128.0, 127.6 (d, *J_CF_* = 3.1 Hz), 127.1, 124.6 (d, *J_CF_* = 3.0 Hz), 123.8, 123.3, 121.6, 120.2, 115.4 (d, *J_CF_* = 21.3 Hz), 111.6, 109.8, 108.6, 42.2 (d, *J_CF_* = 5.3 Hz, PhCH_2_), 41.5 (ArCH_2_Ar); IR (neat) 3063 (w), 2951 (w), 1603 (m), 1509 (s), 1445 (m), 1387 (s), 1224 (s), 1158 (m), 736 (m) cm^–1^; ESIMS calcd for (C_45_H_30_F_2_N_4_O_2_ + H): 697.2415; found 697.2413.

#### 3.3.6. 5,5′-Methylenebis(2-[1-(p-fluorobenzyl)benzimidazol-2-yl]benzofuran) (**3f**)

The standard procedure 2 was followed by use of diamide **15f** (68.4 mg, 90.2 µmol, 1.0 equiv) in AcOH (4.0 mL). After workup, the residue was purified by use of silica gel column chromatography (30% EtOAc in hexanes as the eluent) to give the desired bis(benzofuran–benzimidazole) **3f** (51.5 mg, 73.9 µmol) in 82% overall yield as yellow solids: mp (recrystallized from hexanes/CH_2_Cl_2_) 176.2–177.8 °C; ^1^H NMR (CDCl_3_, 400 MHz) *δ* 7.85 (d, *J* = 7.6 Hz, 2 H, 2 × ArH), 7.42 (d, *J* = 8.4 Hz, 4 H, 4 × ArH), 7.33–7.25 (m, 8 H, 8 × ArH), 7.20–7.10 (m, 6 H, 6 × ArH), 6.91 (d, *J* = 7.2 Hz, 2 H, 2 × ArH), 6.76 (s, 2 H, 2 × ArH), 5.70 (s, 4 H, 2 × CH_2_Ph), 4.13 (s, 2 H, ArCH_2_Ar); ^13^C NMR (CDCl_3_, 100 MHz) *δ* 162.1 (d, *J_CF_* = 245 Hz), 153.9, 146.8, 143.9, 143.2, 136.9, 135.8, 132.0, 131.9, 127.9 (d, *J_CF_* = 8.3 Hz), 127.1, 123.8, 123.2, 121.6, 120.2, 115.8 (d, *J_CF_* = 22.0 Hz), 111.4, 109.9, 108.6, 47.8 (PhCH_2_), 41.5 (ArCH_2_Ar); IR (neat) 2951 (w), 2846 (w), 1603 (m), 1469 (m), 1336 (s), 1224 (s), 1195 (m), 737 (m) cm^–1^; ESIMS calcd for (C_45_H_30_F_2_N_4_O_2_ + H): 697.2415; found 697.2417.

#### 3.3.7. 5,5′-Methylenebis(2-[1-(o-chlorobenzyl)benzimidazol-2-yl]benzofuran) (**3g**) 

The standard procedure 2 was followed by use of diamide **15g** (85.6 mg, 0.112 mmol, 1.0 equiv) in AcOH (5.0 mL). After workup, the residue was purified by use of silica gel column chromatography (35% EtOAc in hexanes as the eluent) to give the desired bis(benzofuran–benzimidazole) **3g** (65.8 mg, 90.3 µmol) in 81% overall yield as off-white solids: mp (recrystallized from hexanes/CH_2_Cl_2_) 170.8–172.2 °C; ^1^H NMR (CDCl_3_, 400 MHz) *δ* 7.88 (d, *J* = 8.0 Hz, 2 H, 2 × ArH), 7.44 (d, *J* = 8.0 Hz, 2 H, 2 × ArH), 7.40–7.30 (m, 6 H, 6 × ArH), 7.28–7.23 (m, 6 H, 6 × ArH), 7.18–7.14 (m, 4 H, 4 × ArH), 7.08 (t, *J* = 7.4 Hz, 2 H, 2 × ArH), 6.56 (t, *J* = 7.4 Hz, 2 H, 2 × ArH), 5.84 (s, 4 H, 2 × CH_2_Ph), 4.10 (s, 2 H, ArCH_2_Ar); ^13^C NMR (CDCl_3_, 100 MHz) *δ* 153.9, 146.5, 144.2, 143.1, 136.8, 136.0, 133.8, 131.9, 129.6, 128.9, 128.0, 127.4, 127.1, 126.7, 123.9, 123.4, 121.6, 120.3, 111.6, 109.8, 108.5, 46.2 (PhCH_2_), 41.5 (ArCH_2_Ar); IR (neat) 2923 (m), 2854 (w), 1515 (w), 1445 (m), 1386 (s), 1332 (m), 1266 (s), 1193 (m), 735 (m) cm^–1^; ESIMS calcd for (C_45_H_30_Cl_2_N_4_O_2_ + H): 729.1824; found 729.1822.

#### 3.3.8. 5,5′-Methylenebis(2-[1-(p-chlorobenzyl)benzimidazol-2-yl]benzofuran) (**3h**) 

The standard procedure 2 was followed by use of diamide **15h** (72.1 mg, 94.3 µmol, 1.0 equiv) in AcOH (4.0 mL). After workup, the residue was purified by use of silica gel column chromatography (35% EtOAc in hexanes as the eluent) to give the desired bis(benzofuran–benzimidazole) **3h** (58.4 mg, 80.1 µmol) in 85% overall yield as white solids: mp (recrystallized from hexanes/CH_2_Cl_2_) 176.0–177.8 °C; ^1^H NMR (CDCl_3_, 400 MHz) *δ* 7.85 (d, *J* = 7.2 Hz, 2 H, 2 × ArH), 7.44 (s, 2 H, 2 × ArH), 7.42 (d, *J* = 7.2 Hz, 2 H, 2 × ArH), 7.33–7.28 (m, 8 H, 8 × ArH), 7.25–7.19 (m, 6 H, 6 × ArH), 7.08 (d, *J* = 7.6 Hz, 4 H, 4 × ArH), 5.74 (s, 4 H, 2 × CH_2_Ph), 4.14 (s, 2 H, ArCH_2_Ar); ^13^C NMR (CDCl_3_, 100 MHz) *δ* 153.9, 146.8, 144.0, 143.2, 136.9, 135.9, 134.8, 133.7, 129.2, 128.0, 127.6, 127.2, 123.9, 123.3, 121.7, 120.3, 111.5, 109.9, 108.8, 47.9 (PhCH_2_), 41.6 (ArCH_2_Ar); IR (neat) 3050 (w), 2923 (m), 2855 (w), 1445 (m), 1387 (s), 1333 (m), 1288 (m), 1194 (m), 736 (m) cm^–1^; ESIMS calcd for (C_45_H_30_Cl_2_N_4_O_2_ + H): 729.1824; found 729.1819.

#### 3.3.9. 5,5′-Methylenebis(2-[1-(m-bromobenzyl)benzimidazol-2-yl]benzofuran) (**3i**)

The standard procedure 2 was followed by use of diamide **15i** (85.9 mg, 101 µmol, 1.0 equiv) in AcOH (5.0 mL). After workup, the residue was purified by use of silica gel column chromatography (35% EtOAc in hexanes as the eluent) to give the desired bis(benzofuran–benzimidazole) **3i** (68.1 mg, 83.4 µmol) in 83% overall yield as white solids: mp (recrystallized from hexanes/CH_2_Cl_2_) 191.0–193.2 °C; ^1^H NMR (CDCl_3_, 400 MHz) *δ* 7.85 (d, *J* = 7.6 Hz, 2 H, 2 × ArH), 7.44 (s, 2 H, 2 × ArH), 7.42 (d, *J* = 7.6 Hz, 2 H, 2 × ArH), 7.37–7.32 (m, 8 H, 8 × ArH), 7.31–7.28 (m, 4 H, 4 × ArH), 7.20 (dd, *J* = 8.4, 1.6 Hz, 2 H, 2 × ArH), 7.12 (t, *J* = 8.4 Hz, 2 H, 2 × ArH), 7.02 (d, *J* = 7.6 Hz, 2 H, 2 × ArH), 5.72 (s, 4 H, 2 × CH_2_Ph), 4.14 (s, 2 H, ArCH_2_Ar); ^13^C NMR (CDCl_3_, 100 MHz) *δ* 153.9, 146.7, 144.0, 143.2, 138.6, 136.9, 135.8, 131.0, 130.5, 129.4, 128.0, 127.2, 124.8, 123.9, 123.4, 123.0, 121.7, 120.2, 111.5, 109.9, 108.8, 47.9 (PhCH_2_), 41.5 (ArCH_2_Ar); IR (neat) 3050 (w), 2918 (w), 1603 (m), 1509 (s), 1444 (m), 1387 (s), 1334 (m), 1224 (s), 1195 (m) cm^–1^; ESIMS calcd for (C_45_H_30_Br_2_N_4_O_2_ + H): 817.0813; found 817.0810.

#### 3.3.10. 5,5′-Methylenebis[2-(1-isopropylbenzimidazol-2-yl)benzofuran] (**3j**) 

The standard procedure 2 was followed by use of diamide **15j** (61.3 mg, 102 µmol, 1.0 equiv) in AcOH (4.0 mL). After workup, the residue was purified by use of silica gel column chromatography (25% EtOAc in hexanes as the eluent) to give the desired bis(benzofuran–benzimidazole) **3j** (50.7 mg, 89.8 µmol) in 88% overall yield as white solids: mp (recrystallized from hexanes/CH_2_Cl_2_) 164.4–166.2 °C; ^1^H NMR (CDCl_3_, 400 MHz) *δ* 7.85–7.80 (m, 2 H, 2 × ArH), 7.62–7.59 (m, 2 H, 2 × ArH), 7.57–7.50 (m, 4 H, 4 × ArH), 7.35 (s, 2 H, 2 × ArH), 7.30–7.25 (m, 6 H, 6 × ArH), 5.43–5.36 (m, 2 H, 2 × HCMe_2_), 4.20 (s, 2 H, ArCH_2_Ar), 1.71 (d, *J* = 6.8 Hz, 12 H, 4 × CH_3_); ^13^C NMR (CDCl_3_, 100 MHz) *δ* 153.9, 147.2, 143.9, 143.6, 136.8, 133.8, 128.1, 126.9, 122.9, 122.5, 121.5, 120.5, 112.4, 111.6, 109.1, 49.1 (CHMe_2_), 41.6 (ArCH_2_Ar), 21.5 (C(CH_3_)_2_); IR (neat) 3054 (w), 2977 (m), 1450 (s), 1367 (s), 1267 (s), 1196 (m), 1132 (m), 740 (s) cm^–1^; ESIMS calcd for (C_37_H_32_N_4_O_2_ + H): 565.2603; found 565.2599.

#### 3.3.11. 5,5′-Methylenebis[2-(1-cyclopentylbenzimidazol-2-yl)benzofuran] (**3k**)

The standard procedure 2 was followed by use of diamide **15k** (70.8 mg, 108 µmol, 1.0 equiv) in AcOH (4.5 mL). After workup, the residue was purified by use of silica gel column chromatography (25% EtOAc in hexanes as the eluent) to give the desired bis(benzofuran–benzimidazole) **3k** (59.2 mg, 96.3 µmol) in 89% overall yield as white solids: mp (recrystallized from hexanes/CH_2_Cl_2_) 172.8–174.6 °C; ^1^H NMR (CDCl_3_, 400 MHz) *δ* 7.85–7.81 (m, 2 H, 2 × ArH), 7.52–7.46 (m, 6 H, 6 × ArH), 7.32 (s, 2 H, 2 × ArH), 7.30–7.25 (m, 4 H, 4 × ArH), 7.23–7.22 (m, 2 H, 2 × ArH), 5.52–5.43 (m, 2 H, 2 × *H*C(CH_2_)_2_), 4.19 (s, 2 H, ArCH_2_Ar), 2.37–2.27 (m, 4 H, 2 × CH_2_), 2.22–2.14 (m, 4 H, 2 × CH_2_), 2.09–2.00 (m, 4 H, 2 × CH_2_), 1.84–1.74 (m, 4 H, 2 × CH_2_); ^13^C NMR (CDCl_3_, 100 MHz) *δ* 153.9, 147.2, 143.5, 143.9, 136.8, 133.5, 128.1, 126.9, 122.9, 122.5, 121.5, 120.6, 112.0, 111.5, 109.0, 57.6 (*C*H(CH_2_)_2_), 41.5 (ArCH_2_Ar), 30.4 (CH_2_), 25.3 (CH_2_); IR (neat) 3053 (w), 2958 (m), 2874 (w), 1450 (s), 1368 (s), 1266 (s), 1196 (m), 740 (s) cm^–1^; ESIMS calcd for (C_41_H_36_N_4_O_2_ + H): 617.2916; found 617.2912.

#### 3.3.12. 5,5′-Methylenebis[2-(1-cyclohexylbenzimidazol-2-yl)benzofuran] (**3l**)

The standard procedure 2 was followed by use of diamide **15l** (76.5 mg, 112 µmol, 1.0 equiv) in AcOH (5.0 mL). After workup, the residue was purified by use of silica gel column chromatography (25% EtOAc in hexanes as the eluent) to give the desired bis(benzofuran–benzimidazole) **3l** (61.6 mg, 95.5 µmol) in 85% overall yield as off-white solids: mp (recrystallized from hexanes/CH_2_Cl_2_) 171.6–173.2 °C; ^1^H NMR (CDCl_3_, 400 MHz) *δ* 7.83–7.81 (m, 2 H, 2 × ArH), 7.667.64 (m, 2 H, 2 × ArH), 7.53 (s, 2 H, 2 × ArH), 7.51 (s, 2 H, 2 × ArH), 7.297.25 (m, 8 H, 8 × ArH), 4.934.85 (m, 2 H, 2 × HCMe_2_), 4.22 (s, 2 H, ArCH_2_Ar), 2.38–2.27 (m, 4 H, 2 × CH_2_), 2.07–1.95 (m, 8 H, 4 × CH_2_), 1.81–1.71 (m, 2 H, CH_2_), 1.50–1.31 (m, 6 H, 3 × CH_2_); ^13^C NMR (CDCl_3_, 100 MHz) *δ* 153.9, 147.2, 143.9 (2 × C), 136.9, 128.2, 126.9, 122.9, 122.5, 121.6, 120.6, 112.8, 111.6, 108.9, 57.4 (CHMe_2_), 41.6 (ArCH_2_Ar), 31.5 (CH_2_), 26.1 (CH_2_), 25.3 (CH_2_); IR (neat) 2958 (m), 2873 (w), 1450 (s), 1339 (m), 1267 (s), 1196 (m), 1164 (m), 741 (s) cm^–1^; ESIMS calcd for (C_43_H_40_N_4_O_2_ + H): 645.3229; found 645.3228.

### 3.4. Biological Evaluation

The antiviral activity of the test compounds against YFV strain 17D (Stamaril) on Huh-7 cells was determined by use of a CPE-based assay. Their potential cytotoxic effects were evaluated in the uninfected host cells. For details to obtain the 50% effective concentration (EC_50_) and 50% cytotoxic concentration (CC_50_), see the descriptions in the published papers of ours [10,64].

### 3.5. Study Plans of QSAR

The quantitative structure–activity relationship (QSAR) provides useful information for the design of new drugs. It involves a machine-learning process that derives the association between independent variables (molecular descriptors and structural features of compounds) and dependent variables. The 2D-QSARs of benzofuran derivatives as vasodilators were reported by Srour et al. [68]. They utilized the CODDESSA PRO program to achieve a robust model, which revealed biological activity of benzofurans. This program has been established by A. R. Katritzky, M. Karelson, and R. Petrukhin of the University of Florida, 2001–2005 [69]. The results supported the applicability of their synthesized benzofuran-based hybrids as potential vasorelaxant active agents. Similar QSAR studies to be performed on our currently synthesized bis-benzofuran derivatives could derive even more potent anti-YFV agents. We shall apply a QSAR mode first to create a relationship between chemical structures and their anti-YFV activity in a data-set of finite drug leads **2** and **3**. Then QSAR models will be used to design new bis(benzofuran–1,3-imidazolidin-4-one)s and bis(benzofuran–1,3-benzimidazole)s with various *N*-substituents and predict their activities. The methods for QSAR analyses are essentially statistical methods. Nevertheless, the CODDESSA PRO program requires a number of descriptors in different families: 8 constitutional, 12 topological, 8 geometrical, 14 electrostatic, 40 CPSA, 7 MO related, 18 quantum chemical, and 7 thermodynamic groups. Its approach differs from the fragment (or group contribution) approach. The descriptors are computed for the system as a whole rather than from the properties of individual fragments. The anti-YFV activity of new bis-conjugates will then be expressed quantitatively as the concentration of a substance required to inhibit the virus. 

On the other hand, the prediction of the partition coefficient log *p* is of special significance. It is an important measure used during the identification of “druglikeness” according to Lipinski′s Rule of Five [70]. The log *p* values can be estimated by chemical fragment methods, which are related to the *N*-substituents to be placed in the bis-conjugates **2** and **3**. The fragmentary values to be determined will be based on the log *p* values shown in the last column of Table 1 for the reporting compounds. An advanced approach on fragment-based QSAR can strengthen the concept of pharmacophore similarity and will be developed by use of the data associated with the reporting bis-conjugates. This pharmacophore-similarity-based QSAR method involves the use of topological pharmacophoric descriptors. Property prediction will assist the contribution of pharmacophore features encoded by the benzofuran, imidazolidinone, and benzimidazole moieties toward biological activity improvement [71]. These complex and useful QSAR analyses will be performed in due course.

## 4. Conclusions

We designed and synthesized two series of bis(benzofuran–1,3-*N*,*N*-heterocycle) conjugated compounds. Among 23 new conjugates, 4 (i.e., **2c**, **2e**, **2k**, and **3j**) inhibited YFV strain 17D (Stamaril) on Huh-7 cells with EC_50_ values of 3.54–8.89 μM. The most appealing SI values associated with these conjugates were in the range of 6.07–15.3. Moreover, their structure–activity relationship was illustrated. Attachment of imidazolidinone or benzimidazole moieties to the bisbenzofuran nuclei greatly increased the YFV inhibition. The substituents on the *N*-atoms in the imidazolidinone and benzimidazole moieties influenced their anti-YFV activities. These findings provide clues for such types of dimeric compounds to be developed as antiviral agents. 

## Figures and Tables

**Scheme 1:**
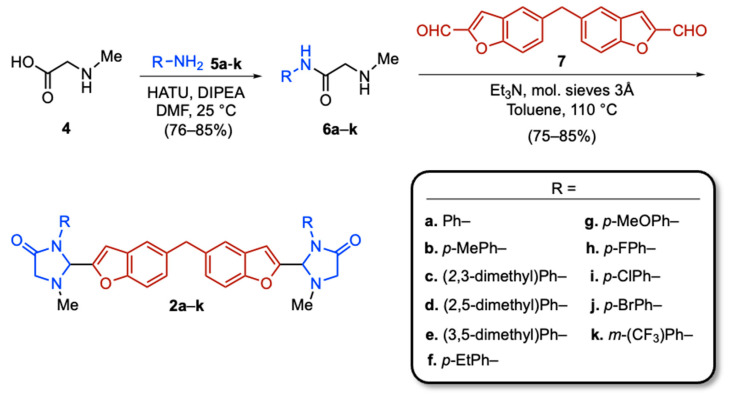
Synthesis of bis(benzofuran–imidazolidin-4-one)s **2**.

**Table 1:** Biological activity of conjugated compounds and their lipophilicity.

Bis-Conjugate	CC_50_ (μM)	EC_50_ (μM)	SI	log *p*
**2a**	–	>133	–	4.75
**2b**	−	>160	−	−
**2c**	41.0	6.75	6.07	3.77
**2d**	18.5	7.7	2.40	4.38
**2e**	54.1	3.54	15.3	4.15
**2f**	28.5	8.51	3.35	4.68
**2g**	–	>152	–	4.25
**2h**	38.5	23.5	1.63	4.60
**2i**	12.3	14.4	0.85	**–**
**2j**	–	>133	−	**–**
**2k**	75.0	8.89	8.43	5.16
**3a**	–	>114	–	**–**
**3b**	–	>145	–	**–**
**3c**	–	>139	–	**–**
**3d**	–	27.7	–	**–**
**3e**	–	>21.5	–	4.65
**3f**	–	28.7	–	**–**
**3g**	–	>137	–	**–**
**3h**	–	>103	–	**–**
**3i**	–	91.6	–	**–**
**3j**	69.3	6.14	11.3	3.71
**3k**	–	>162	–	**–**
**3l**	–	>28.7	–	**–**
**7**	–	>329	–	3.75
**10**	–	>255	–	**–**
**11**	–	>297	–	**–**

## Data Availability

The datasets generated and analyzed during the current study are available from the corresponding authors on reasonable request.

## Supplementary Materials

The following supporting information can be downloaded at: https://www.mdpi.com/article/10.3390/ijms232012675/s1. References [60,72,73,74,75,76,77,78,79,80,81,82] are cited in the Supplementary Materials.
