# Supplementary material for: Bis(Benzofuran–1,3-N,N-heterocycle)s as Symmetric and Synthetic Drug Leads against Yellow Fever Virus
Source: Int J Mol Sci. 2022 Oct 21;23(20):12675. doi: 10.3390/ijms232012675 (PMC9604066; doi:10.3390/ijms232012675)
Supplement: Supplementary file 1 [file ijms-23-12675-s001.zip › ijms-1904213-supplementary.pdf]

Supplementary Materials

Bis(benzofuran–1,3-*N*,*N*-heterocycle)s as Symmetric and Synthetic Drug Leads against Yellow Fever Virus

Nitesh K. Gupta,[1] Srinivasan Jayakumar,[1] Wen-Chieh Huang,[1] Pieter Leyssen,[2] Johan Neyts,[2] Sergey O. Bachurin,[3] Jih Ru Hwu,[1,4,*] and Shwu-Chen Tsay[1,4,*]

1 Department of Chemistry & Frontier Research Center on Fundamental and Applied Sciences of Matters, National Tsing Hua University, Hsinchu 30013, Taiwan
2 Rega Institute for Medical Research, Katholieke Universiteit Leuven, Minderbroedersstraat 10, B-3000 Leuven, Belgium
3 The Institute of Physiologically Active Compounds, Russian Academy of Sciences, Chernogolovka 142432, Russia
4 Department of Chemistry, National Central University, Jhongli City 32001, Taiwan

* Correspondence: sctsay@mx.nthu.edu.tw (S.-C. T.), jrhwu@mx.nthu.edu.tw (J.R.H.)

Table of Contents

Preparation of *N*-Arylamides **6** (Standard Procedure 1) 2

Preparation of Diethyl-5,5'-methylenebis(benzofuran-2-carboxylate) (**10**) 5

Preparation of 5,5'-Methylenebis(benzofuran-2-carboxylic acid) (**11**) 5

Preparation of Mono-*N*-alkylated-2-phenylenediamine (**14**) (Standard Procedure 2) 5

Preparation of Diamide (**15**) (Standard Procedure 3) 9

References 14

^{1}H NMR and ^{13}C NMR Spectra of Compounds **2**, **10**, **11**, and **3** 15

S1. Preparation of N-Arylamides ***6*** *(Standard Procedure 1)*

To a solution of *N*-methylglycine (**4**, 1.0 equiv) in DMF (5.0–7.5 mL) were added anilines **5** (1.5 equiv), *N*,*N*-diisopropylethylamine (DIPEA, 2.1 equiv), and 1-[bis(dimethylamino)methylene]-1H-1,2,3-triazolo[4,5-b]pyridinium 3-oxide hexafluorophosphate (HATU, 1.1 equiv) at 0 °C. The reaction mixture was stirred at room temperature for 12–15 h and then concentrated under vacuum. The residue was diluted with EtOAc (100 mL), washed with 1.0 N aqueous HCl (10 × 2 mL), saturated aqueous $NaHCO_3$ (15 × 3 mL) and brine, and then dried over anhydrous $MgSO_4$. The organic layer was evaporated under vacuum. The resultant residue was purified by use of silica gel column chromatography (a mixture of EtOAc to hexanes) to give the desired *N*-arylamides **6**.

S1.1. *N*-Phenyl-2-(methylamino)acetamide (**6a**)

The standard procedure 1 was followed by use of *N*-methylglycine (4, 95.1 mg, 1.06 mmol, 1.0 equiv), aniline (5a, 148 mg, 1.60 mmol, 1.5 equiv), DIPEA (287 mg, 2.22 mmol, 2.1 equiv), HATU (443 mg, 1.16 mmol, 1.1 equiv), and DMF (5.0 mL). After workup, the residue was purified by use of silica gel column chromatography (70% EtOAc in hexanes as the eluent) to give the desired amide 6a (148 mg, 0.906 mmol) in 85% overall yield as white solids: ^{1}H NMR ($CDCl_3$, 400 MHz) δ 7.58 (d, J = 8.4 Hz, 2 H, 2 × ArH), 7.31 (t, J = 7.4 Hz, 2 H, 2 × ArH), 7.08 (t, J = 7.4 Hz, 1 H, 1 × ArH), 3.34 (s, 2 H, NCH_2CO), 2.48 (s, 3 H, NCH_3). The spectroscopic data was in accordance with those reported in the literature [72].

S1.2. *N*-(4-Methylphenyl)-2-(methylamino)acetamide (**6b**)

The standard procedure 1 was followed by use of *N*-methylglycine (**4**, 96.1 mg, 1.07 mmol, 1.0 equiv), *p*-methylaniline (**5b**, 171 mg, 1.61 mmol, 1.5 equiv), DIPEA (288 mg, 2.21 mmol, 2.1 equiv), HATU (440 mg, 1.17 mmol, 1.1 equiv), and DMF (5.5 mL). After workup, the residue was purified by use of silica gel column chromatography (70% EtOAc in hexanes as the eluent) to give the desired amide **6b** (153 mg, 0.862 mmol) in 80% overall yield as white solids: ^{1}H NMR (CD_3OD, 400 MHz) δ 7.43 (d, J = 8.0 Hz, 2 H, 2 × ArH), 7.08 (d, J = 8.0 Hz, 2 H, 2 × ArH), 3.27 (s, 2 H, NCH_2CO), 2.42 (s, 3 H, NCH_3), 2.27 (s, 3 H, $ArCH_3$); ^{13}C NMR (CD_3OD, 100 MHz) δ 169.51 (C=O), 135.04, 133.48, 129.29, 119.36, 54.89 (CH_2N), 36.63 (NCH_3), 20.70 ($ArCH_3$). The spectroscopic data was in accordance with those reported in the literature [73].

S1.3. *N*-(2,3-Dimethylphenyl)-2-(methylamino)acetamide (**6c**)

The standard procedure 1 was followed by use of *N*-methylglycine (**4**, 101 mg, 1.13 mmol, 1.0 equiv), 2,3-dimethylaniline (**5c**, 206 mg, 1.70 mmol, 1.5 equiv), DIPEA (307 mg, 2.37 mmol, 2.1 equiv), HATU (472 mg, 1.24 mmol, 1.1 equiv), and DMF (6.5 mL). After workup, the residue was purified by use of silica gel column chromatography (65% EtOAc in hexanes as the eluent) to give the desired amide **6c** (185 mg, 0.963 mmol) in 85% overall yield as off-white solids: ^{1}H NMR (CD_3OD, 400 MHz) δ 7.20–7.19 (m, 1 H, ArH), 7.18–7.10 (m, 2 H, 2 × ArH), 4.05 (s, 2 H, NCH_2CO), 2.80 (s, 3 H, NCH_3), 2.33 (s, 3 H, $ArCH_3$), 2.18 (s, 3 H, $ArCH_3$). The spectroscopic data was in accordance with those reported in the literature [74].

S1.4. *N*-(2,5-Dimethylphenyl)-2-(methylamino)acetamide (**6d**)

The standard procedure 1 was followed by use of *N*-methylglycine (**4**, 105 mg, 1.17 mmol, 1.0 equiv), 2,5-dimethylaniline (**5d**, 214 mg, 1.76 mmol, 1.5 equiv), DIPEA (317 mg, 2.46 mmol, 2.1 equiv), and HATU (490 mg, 1.29 mmol, 1.1 equiv), and DMF (6.0 mL). After workup, the residue was purified by use of silica gel column chromatography (75% EtOAc in hexanes as the eluent) to give the desired amide **6d** (184 mg, 0.959 mmol) in 82% overall yield as white solids: ^{1}H NMR (CD_3OD, 400 MHz) δ 7.24 (s, 1 H, ArH), 7.12 (d, J = 7.4 Hz, 1 H, ArH), 6.98 (d, J = 7.4 Hz, 1 H, ArH), 4.00 (s, 2 H, NCH_2CO), 2.78 (s, 3 H, NCH_3), 2.29 (s, 3 H, $ArCH_3$), 2.21 (s, 3 H, $ArCH_3$); ^{13}C NMR (CD_3OD, 100 MHz) δ 165.3 (C=O), 137.2, 135.6, 131.5, 130.5, 128.4, 126.9, 50.8 (CH_2N), 33.5 (NCH_3), 20.9 ($ArCH_3$), 17.5 ($ArCH_3$); EIMS calcd for ($C_{11}H_{16}N_2O$): 192.1263; found 192.1265.

S1.5. *N*-(3,5-Dimethylphenyl)-2-(methylamino)acetamide (**6e**)

The standard procedure 1 was followed by use of *N*-methylglycine (**4**, 108 mg, 1.21 mmol, 1.0 equiv), 3,5-dimethylaniline (**5e**, 220 mg, 1.81 mmol, 1.5 equiv), DIPEA (328 mg, 2.54 mmol, 2.1 equiv), HATU (506 mg, 1.33 mmol, 1.1 equiv), and DMF (7.0 mL). After workup, the residue was purified by use of silica gel column chromatography (75% EtOAc in hexanes as the eluent) to give the desired amide **6e** (180 mg, 0.936 mmol) in 80% overall yield as white solids: ^{1}H NMR (CD_3OD, 400 MHz) δ 7.19 (s, 2 H, 2 × ArH), 6.80 (s, 1 H, 1 × ArH), 3.91 (s, 2 H, NCH_2CO), 2.76 (s, 3 H, NCH_3), 2.27 (s, 6 H, 2 × $ArCH_3$); 13C NMR (CD_3OD, 100 MHz) δ 164.4 (C=O), 138.8, 138.7, 127.2, 118.7, 51.1 (CH_2N), 33.5 (NCH_3), 21.4 ($ArCH_3$); EIMS calcd for ($C_{11}H_{16}N_2O$): 192.1263; found 192.1261.

S1.6. *N*-(2-Ethylphenyl)-2-(methylamino)acetamide (**6f**)

The standard procedure 1 was followed by use of *N*-methylglycine (**4**, 101 mg, 1.13 mmol, 1.0 equiv), *o*-ethylaniline (**5f**, 205 mg, 1.70 mmol, 1.5 equiv), DIPEA (307 mg, 2.37 mmol, 2.1 equiv), HATU (472 mg, 1.24 mmol, 1.1 equiv), and DMF (6.5 mL). After workup, the residue was purified by use of silica gel column chromatography (80% EtOAc in hexanes as the eluent) to give the desired amide **6f** (185 mg, 0.971 mmol) in 85% overall yield as pale yellow solids: ^{1}H NMR (CD_3OD, 700 MHz) δ 7.38 (d, J = 7.7 Hz, 1 H, ArH), 7.29 (d, J = 7.7 Hz, 1 H, ArH), 7.22–7.20 (m, 2 H, 2 × ArH), 4.04 (s, 2 H, NCH_2CO), 2.79 (s, 3 H, NCH_3), 2.65 (q, J = 7.5 Hz, 2 H, $ArCH_2CH_3$), 1.20 (t, J = 7.5 Hz, 3 H, $ArCH_2CH_3$); ^{13}C NMR (CD_3OD, 175 MHz) δ 165.8 (C=O), 140.1, 135.1, 130.0, 128.2, 127.4, 127.3, 50.8 (CH_2N), 33.5 (NCH_3), 25.1 ($ArCH_2CH_3$); 14.8 ($ArCH_2CH_3$); EIMS calcd for ($C_{11}H_{16}N_2O$): 192.1263; found 192.1262.

S1.7. *N*-(4-Methoxyphenyl)-2-(methylamino)acetamide (**6g**)

The standard procedure 1 was followed by use of *N*-methylglycine (**4**, 125 mg, 1.40 mmol, 1.0 equiv), *p*-methoxylaniline (**5g**, 258 mg, 2.10 mmol, 1.5 equiv), DIPEA (380 mg, 2.94 mmol, 2.1 equiv), HATU (586 mg, 1.54 mmol, 1.1 equiv), and DMF (7.5 mL). After workup, the residue was purified by use of silica gel column chromatography (75% EtOAc in hexanes as the eluent) to give the desired amide **6g** (226 mg, 1.16 mmol) in 83% overall yield as white solids: ^{1}H NMR (CD_3OD, 400 MHz) δ 7.43 (d, J = 9.4 Hz, 2 H, 2 × ArH), 6.81 (d, J = 9.4 Hz, 2 H, 2 × ArH), 3.75

(s, 3H, OCH_3), 3.32 (s, 2 H, NCH_2CO), 2.44 (s, 3 H, NCH_3). The spectroscopic data was in accordance with those reported in the literature [75].

S1.8. *N*-(4-Fluorophenyl)-2-(methylamino)acetamide (**6h**)

The standard procedure 1 was followed by use of *N*-methylglycine (**4**, 125 mg, 1.40 mmol, 1.0 equiv), *p*-fluoroaniline (**5h**, 233 mg, 2.10 mmol, 1.5 equiv), DIPEA (380 mg, 2.95 mmol, 2.1 equiv), HATU (586 mg, 1.54 mmol, 1.1 equiv), and DMF (7.5 mL). After workup, the residue was purified by use of silica gel column chromatography (80% EtOAc in hexanes as the eluent) to give the desired amide **6h** (199 mg, 1.09 mmol) in 78% overall yield as white solids: ^{1}H NMR (CD_3OD, 400 MHz) δ 7.69–7.56 (m, 2 H, 2 × ArH), 7.09–7.05 (m, 2 H, 2 × ArH), 3.94 (s, 2 H, NCH_2CO), 2.77 (s, 3 H, NCH_3); ^{13}C NMR (CD_3OD, 100 MHz) δ 164.5 (C=O), 160.8 (d, J_{CF} = 241 Hz), 135.2, 122.8 (d, J_{CF} = 7.6 Hz), 116.4 (d, J_{CF} = 22 Hz), 51.0 (CH_2N), 33.5 (NCH_3); ESIMS calcd for ($C_9H_{11}FN_2O$ + H): 183.0934; found 183.0929.

S1.9. *N*-(4-Chlorophenyl)-2-(methylamino)acetamide (**6i**)

The standard procedure 1 was followed by use of *N*-methylglycine (**4**, 115 mg, 1.29 mmol, 1.0 equiv), *p*-chloroaniline (**5i**, 245 mg, 1.93 mmol, 1.5 equiv), DIPEA (350 mg, 2.70 mmol, 2.1 equiv), HATU (539 mg, 1.42 mmol, 1.1 equiv), and DMF (6.0 mL). After workup, the residue was purified by use of silica gel column chromatography (75% EtOAc in hexanes as the eluent) to give the desired amide **6i** (212 mg, 1.07 mmol) in 83% overall yield as white solids: ^{1}H NMR (CD_3OD, 400 MHz) δ 7.53 (d, *J* = 7.2 Hz, 2 H, 2 × ArH), 7.27 (d, *J* = 7.2 Hz, 2 H, 2 × ArH), 3.90 (s, 2 H, NCH_2CO), 2.72 (s, 3 H, NCH_3); ^{13}C NMR (CD_3OD, 100 MHz) δ 164.71 (C=O), 137.88, 130.53, 129.98, 122.29, 51.13 (CH_2N), 33.56 (NCH_3); ESIMS calcd for ($C_9H_{11}ClN_2O$ + H): 199.0638; found 199.0642.

S1.10. *N*-(4-Bromophenyl)-2-(methylamino)acetamide (**6j**)

The standard procedure 1 was followed by use of *N*-methylglycine (**4**, 110 mg, 1.23 mmol, 1.0 equiv), *p*-bromoaniline (**5j**, 316 mg, 1.85 mmol, 1.5 equiv), DIPEA (333 mg, 2.58 mmol, 2.1 equiv), HATU (514 mg, 1.35 mmol, 1.1 equiv), and DMF (5.5 mL). After workup, the residue was purified by use of silica gel column chromatography (80% EtOAc in hexanes as the eluent) to give the desired amide **6j** (227 mg, 0.93 mmol) in 76% overall yield as white solids: ^{1}H NMR (CD_3OD, 400 MHz) δ 7.48 (d, *J* = 8.0 Hz, 2 H, 2 × ArH), 7.41 (d, *J* = 8.0 Hz, 2 H, 2 × ArH), 3.32 (s, 2 H, NCH_2CO), 2.47 (s, 3 H, NCH_3). The spectroscopic data was in accordance with those reported in the literature [72].

S1.11. *N*-(3-Trifluoromethylphenyl)-2-(methylamino)acetamide (**6k**)

The standard procedure 1 was followed by use of *N*-methylglycine (**4**, 105 mg, 1.17 mmol, 1.0 equiv), *m*-trifluoromethylaniline (**5k**, 285 mg, 1.76 mmol, 1.5 equiv), DIPEA (317 mg, 2.46 mmol, 2.1 equiv), HATU (489 mg, 1.29 mmol, 1.1 equiv), and DMF (5.5 mL). After workup, the residue was purified by use of silica gel column chromatography (75% EtOAc in hexanes as the eluent) to give the desired amide **6k** (208 mg, 0.896 mmol) in 76% overall yield as white solids: ^{1}H NMR (CD_3OD, 400 MHz) δ 8.05 (s, 1 H, ArH), 7.74 (d, *J* = 8.0 Hz, 1 H, ArH), 7.53–7.51 (m,

2 H, 2 × ArH), 3.99 (s, 2 H, NCH_2CO), 2.79 (s, 3 H, NCH_3). The spectroscopic data was in accordance with those reported in the literature [76].

*S2. Preparation of Diethyl-5,5'-methylenebis(benzofuran-2-carboxylate) (**10**)*

To a solution of bissalicylaldehyde [60] **8** (2.02 g, 7.88 mmol, 1.0 equiv) in dry DMF (25 mL) was added K_2CO_3 (s, 6.52 g, 47.2 mmol, 6.0 equiv), and ethyl bromoacetate (**9**, 2.88 g, 17.3 mmol, 2.2 equiv) under N_2 atmosphere. The reaction mixture was heated to 120 °C with stirring for 16 h. After being cooled to room temperature, it was diluted and extracted with diethyl ether (50 mL). The organic layer was then washed with brine (3 × 30 mL), H_2O (3 × 30 mL), dried over $MgSO_4$ (s), filtered, and concentrated under reduced pressure. The residue was purified by use of silica gel column chromatography (5% EtOAc in hexanes as the eluent) to give the desired bis(benzofuran-2-carboxylate) **10** (2.35 g, 5.99 mmol) in 76% yield as white solids: mp (recrystallized from dichloromethane/hexanes) 162.8–164.2 °C; ^{1}H NMR ($CDCl_3$, 400 MHz) δ 7.49 (d, J = 8.6 Hz, 2 H, 2 × ArH), 7.45 (s, 2 H, 2 × ArH), 7.43 (s, 2 H, 2 × HC=CO), 7.26 (dd, J = 8.6, 1.6 Hz, 2 H, 2 × ArH), 4.41 (q, J = 7.2 Hz, 4 H, 2 × OCH_2Me), 4.15 (s, 2 H, $ArCH_2Ar$), 1.39 (t, J = 7.2 Hz, 6 H, 2 × CH_3); ^{13}C NMR ($CDCl_3$, 100 MHz) δ 159.4 (C=O), 154.4, 145.9, 136.7, 128.7, 127.1, 122.3, 113.5, 112.2, 61.3 (OCH_2), 41.3 ($ArCH_2Ar$), 14.2 (CH_3); IR (neat) 2984 (m), 2932 (m), 1722 (s, C=O), 1573 (s), 1290 (s), 1224 (s), 1088 (m), 950 (w) cm^{-1}; ESIMS calcd for ($C_{23}H_{20}O_6$): 392.1260; found 392.1259.

*S3. Preparation of 5,5'-Methylenebis(benzofuran-2-carboxylic acid) (**11**)*

To a solution of bis(benzofuran-2-carboxylate) **10** (0.201 g, 0.512 mmol, 1.0 equiv) in THF (2.0 mL) was added 2 N KOH (4.0 mL, 1:1) and EtOH. After the reaction mixture was stirred at 25 °C for 4.0 h, the organic layer was removed under reduced pressure. Then the residue was dissolved in water (20 mL) and acidified with conc. HCl (5 mL). The aqueous layer was extracted with EtOAc (3 × 50 mL). Subsequently, the combined organic layers were dried over $MgSO_4$ (s), filtered, and concentrated under reduced pressure. The solid residue was crystallized by addition of pentane to give the desired bis(benzofuran-2-carboxylic acid) **11** (141 g, 0.419 mmol) in 82% yield as white solids: mp (recrystallized from EtOH) 185.2–187.6 °C; ^{1}H NMR (DMSO-d_6, 400 MHz) δ 7.61–7.59 (m, 6 H, 6 × ArH), 7.39 (d, J = 8.4 Hz, 2 H, 2 × ArH), 4.16 (s, 2 H, $ArCH_2Ar$); ^{13}C NMR (DMSO-d_6, 100 MHz) δ 160.1 (C=O), 153.7, 146.5, 137.2, 128.7, 127.1, 122.5, 113.5, 112.0, 40.5 ($ArCH_2Ar$); IR (neat) 2924 (m), 1689 (s, C=O), 1576 (s), 1421 (w), 1298 (s), 1198 (m), 943 (w), 761 (w) cm^{-1}; ESIMS calcd for ($C_{19}H_{12}O_6$): 336.0634; found 336.0634.

*S4. Preparation of Mono-N-alkylated-2-phenylenediamine (**14**) (Standard Procedure 2)*

The solution of *o*-phenylenediamine (**12**, 1.0 equiv) and aldehyde or ketone **13** (0.25 equiv) in dry MeOH (5.0–7.5 mL) was stirred at 0 °C for 2.0 h under nitrogen atmosphere. Then $NaBH_4$ (0.25 equiv) was added to the reaction mixture portion wise. After the reaction mixture was heated to 25 °C with stirring for 6.0 h, the mixture was concentrated under reduce pressure. The residue was diluted with EtOAc (50 mL), washed with H_2O (2 × 20 mL) and then brine (2 × 20 mL), dried over $MgSO_4$ (s), filtered, and concentrated under reduced pressure. The residue was purified by use of silica gel column chromatography (a mixture of EtOAc and hexanes) to give *N*-alkylated-2-phenylenediamine **14**.

S4.1. *N*-Benzylbenzene-1,2-diamine (**14a**)

The standard procedure 2 was followed by use of *o*-phenylenediamine (**12**, 451 mg, 4.17 mmol, 1.0 equiv), benzaldehyde (**13a**, 114 mg, 1.04 mmol, 0.25 equiv), $NaBH_4$ (39.4 mg, 1.04 mmol, 0.25 equiv), and MeOH (5.5 mL). After workup, the residue was purified by use of silica gel column chromatography (5% EtOAc in hexanes as the eluent) to give the desired *N*-alkylated-1,2-diamine **14a** (644 mg, 3.25 mmol) in 78% overall yield as a brown liquid: ^1H NMR ($CDCl_3$, 400 MHz) δ 7.42–7.35 (m, 5 H, 5 × ArH), 6.93–6.89 (m, 1 H, ArH), 6.82–6.75 (m, 3 H, 3 × ArH), 4.37 (s, 2 H, CH_2Ph). The spectroscopic data was in accordance with those reported in the literature [77].

S4.2. *N*-(4-Methylbenzyl)benzene-1,2-diamine (**14b**)

The standard procedure 2 was followed by use of *o*-phenylenediamine (**12**, 455 mg, 4.18 mmol, 1.0 equiv), *p*-methylbenzaldehyde (**13b**, 125 mg, 1.05 mmol, 0.25 equiv), $NaBH_4$ (39.7 mg, 1.04 mmol, 0.25 equiv), and MeOH (6.0 mL). After workup, the residue was purified by use of silica gel column chromatography (5% EtOAc in hexanes as the eluent) to give the desired *N*-alkylated-1,2-diamine **14b** (669 mg, 3.15 mmol) in 75% overall yield as a brown liquid: ^1H NMR ($CDCl_3$, 400 MHz) δ 7.28 (d, J = 7.8 Hz, 2 H, 2 × ArH), 7.15 (d, J = 7.8 Hz, 2 H, 2 × ArH), 6.80–6.78 (m, 1 H, ArH), 6.74–6.67 (m, 3 H, 3 × ArH), 4.26 (s, 2 H, CH_2Ph), 2.35 (s, 3 H, CH_3). The spectroscopic data was in accordance with those reported in the literature [78].

S4.3. *N*-(3-Methoxybenzyl)benzene-1,2-diamine (**14c**)

The standard procedure 2 was followed by use of *o*-phenylenediamine (**12**, 502 mg, 4.64 mmol, 1.0 equiv), *m*-methoxybenzaldehyde (**13c**, 158 mg, 1.16 mmol, 0.25 equiv), $NaBH_4$ (43.8 mg, 1.16 mmol, 0.25 equiv), and MeOH (7.5 mL). After workup, the residue was purified by use of silica gel column chromatography (10% EtOAc in hexanes as the eluent) to give the desired *N*-alkylated-1,2-diamine **14c** (815 mg, 3.57 mmol) in 77% overall yield as a brown liquid: ^1H NMR ($CDCl_3$, 400 MHz) δ 7.31 (t, J = 8.2 Hz, 1 H, 1 × ArH), 7.02 (d, J = 8.2 Hz, 2 H, 2 × ArH), 6.88–6.83 (m, 2 H, 2 × ArH), 6.75–6.69 (m, 3 H, 3 × ArH), 4.31 (s, 2 H, CH_2Ph), 3.83 (s, 3 H, OCH_3); ^{13}C NMR ($CDCl_3$, 100 MHz) δ 159.7, 141.0, 137.5, 134.0, 129.4, 120.5, 119.9, 118.6, 116.3, 113.2, 112.4, 111.8, 55.0 (OCH_3), 48.4 (CH_2Ph); IR (neat) 3353 (m, NH), 2937 (m), 2835 (m), 1599 (s), 1505 (s), 1455 (s), 1266 (s), 743 (w) cm^{-1}; ESIMS calcd for ($C_{14}H_{16}N_2O$ + H): 229.1341; found 229.1340.

S4.4. *N*-(4-Methoxybenzyl)benzene-1,2-diamine (**14d**)

The standard procedure 2 was followed by use of *o*-phenylenediamine (**12**, 505 mg, 4.65 mmol, 1.0 equiv), *p*-methoxybenzaldehyde (**13d**, 127 mg, 1.18 mmol, 0.25 equiv), $NaBH_4$ (39.7 mg, 1.18 mmol, 0.25 equiv), and MeOH (7.5 mL). After workup, the residue was purified by use of silica gel column chromatography (10% EtOAc in hexanes as the eluent) to give the desired *N*-alkylated-1,2-diamine **14d** (852 mg, 3.73 mmol) in 80% overall yield as a black liquid: ^1H NMR ($CDCl_3$, 400 MHz) δ 7.34 (d, J = 8.8 Hz, 2 H, 2 × ArH), 6.92 (d, J = 8.8 Hz, 2 H, 2 × ArH), 6.86–6.82 (m, 1 H, 1 × ArH), 6.74–6.70 (m, 3 H, 3 × ArH), 4.25 (s, 2 H, CH_2Ph), 3.82 (s, 3 H, OCH_3). The spectroscopic data was in accordance with those reported in the literature [79].

S4.5. *N*-(2-Fluorobenzyl)benzene-1,2-diamine (**14e**)

The standard procedure 2 was followed by use of *o*-phenylenediamine (**12**, 251 mg, 2.33 mmol, 1.0 equiv), *o*-fluorobenzaldehyde (**13e**, 63.5 mg, 0.591 mmol, 0.25 equiv), $NaBH_4$ (19.8 mg, 0.591 mmol, 0.25 equiv), and MeOH (5.0 mL). After workup, the residue was purified by use of silica gel column chromatography (10% EtOAc in hexanes as the eluent) to give the desired *N*-alkylated-1,2-diamine **14e** (351 mg, 1.62 mmol) in 70% overall yield as a brown liquid: ^{1}H NMR ($CDCl_3$, 400 MHz) δ 7.37–7.29 (m, 1 H, ArH), 7.26–7.23 (m, 1 H, ArH), 7.14–7.05 (m, 2 H, 2 × ArH), 6.82–6.80 (m, 1 H, ArH), 6.79–6.72 (m, 1 H, ArH), 6.68 (d, J = 7.6 Hz, 2 H, 2 × ArH), 4.38 (s, 2 H, CH_2Ph). The spectroscopic data was in accordance with those reported in the literature [80].

S4.6. *N*-(4-Fluorobenzyl)benzene-1,2-diamine (**14f**)

The standard procedure 2 was followed by use of *o*-phenylenediamine (**12**, 501 mg, 4.63 mmol, 1.0 equiv), *p*-fluorobenzaldehyde (**13f**, 127 mg, 1.15 mmol, 0.25 equiv), $NaBH_4$ (39.6 mg, 1.15 mmol, 0.25 equiv), and MeOH (7.5 mL). After workup, the residue was purified by use of silica gel column chromatography (10% EtOAc in hexanes as the eluent) to give the desired *N*-alkylated-1,2-diamine **14f** (721 mg, 3.33 mmol) in 72% overall yield as a black liquid: ^{1}H NMR ($CDCl_3$, 400 MHz) δ 7.38–7.34 (m, 2 H, ArH), 7.04 (t, J = 8.4 Hz, 2 H, 2 × ArH), 6.85–6.66 (m, 4 H, ArH), 4.28 (s, 2 H, CH_2Ph). The spectroscopic data was in accordance with those reported in the literature [81].

S4.7. *N*-(2-Chlorobenzyl)benzene-1,2-diamine (**14g**)

The standard procedure 2 was followed by use of *o*-phenylenediamine (**12**, 504 mg, 4.65 mmol, 1.0 equiv), *o*-chlorobenzaldehyde (**13g**, 161 mg, 1.16 mmol, 0.25 equiv), $NaBH_4$ (43.8 mg, 1.16 mmol, 0.25 equiv), and MeOH (7.5 mL). After workup, the residue was purified by use of silica gel column chromatography (10% EtOAc in hexanes as the eluent) to give the desired *N*-alkylated-1,2-diamine **14g** (811 mg, 3.49 mmol) in 75% overall yield as a brown liquid: ^{1}H NMR ($CDCl_3$, 400 MHz) δ 7.43–7.38 (m, 2 H, 2 × ArH), 7.26–7.20 (m, 2 H, 2 × ArH), 6.82–6.69 (m, 2 H, 2 × ArH), 6.68 (dd, J = 7.6, 1.2 Hz, 2 H, 2 × ArH), 4.44 (s, 2 H, CH_2Ph); ^{13}C NMR ($CDCl_3$, 100 MHz) δ 137.2, 136.6, 134.3, 133.4, 129.5, 129.2, 128.4, 126.9, 120.7, 119.0, 116.7, 112.3, 46.1 (CH_2Ph); IR (neat) 3399 (m, NH), 3335 (m, NH), 2923 (m), 2850 (m), 1598 (m), 1506 (s), 1455 (m), 1271 (s), 1091 (w) cm^{-1}.

S4.8. *N*-(4-Chlorobenzyl)benzene-1,2-diamine (**14h**)

The standard procedure 2 was followed by use of *o*-phenylenediamine (**12**, 509 mg, 4.71 mmol, 1.0 equiv), *p*-chlorobenzaldehyde (**13h**, 165 mg, 1.17 mmol, 0.25 equiv), $NaBH_4$ (44.2 mg, 1.17 mmol, 0.25 equiv), and MeOH (7.5 mL). After workup, the residue was purified by use of silica gel column chromatography (15% EtOAc in hexanes as the eluent) to give the desired *N*-alkylated-1,2-diamine **14h** (784 mg, 3.37 mmol) in 72% overall yield as a black liquid: ^{1}H NMR ($CDCl_3$, 400 MHz) δ 7.32–7.30 (m, 4 H, 4 × ArH), 6.78–6.68 (m, 3 H, 3 × ArH), 6.59 (d, J = 7.6 Hz, 1 H, ArH), 4.27 (s, 2 H, CH_2Ph). The spectroscopic data was in accordance with those reported in the literature [82].

S4.9. *N*-(3-Bromobenzyl)benzene-1,2-diamine (**14i**)

The standard procedure 2 was followed by use of *o*-phenylenediamine (**12**, 501 mg, 4.63 mmol, 1.0 equiv), *m*-bromobenzaldehyde (**13i**, 214 mg, 1.15 mmol, 0.25 equiv), $NaBH_4$ (43.5 mg, 1.15 mmol, 0.25 equiv), and MeOH (7.0 mL). After workup, the residue was purified by use of silica gel column chromatography (15% EtOAc in hexanes as the eluent) to give the desired *N*-alkylated-1,2-diamine **14i** (784 mg, 3.37 mmol) in 72% overall yield as a black liquid: ^{1}H NMR ($CDCl_3$, 400 MHz) δ 7.57 (s, 1 H, ArH), 7.42 (d, J = 7.2 Hz, 1 H, ArH), 7.31 (d, J = 7.2 Hz, 1 H, ArH), 7.21 (t, J = 8.0 Hz, 1 H, ArH), 6.82–6.74 (m, 3 H, 3 × ArH), 6.62 (d, J = 7.2 Hz, 1 H, ArH), 4.28 (s, 2 H, CH_2Ph); ^{13}C NMR ($CDCl_3$, 100 MHz) δ 141.8, 137.2, 134.0, 130.4, 130.2, 130.0, 126.1, 122.6, 120.6, 118.9, 116.6, 111.9, 47.8 (CH_2Ph); IR (neat) 3350 (m, NH), 2925 (m), 2868 (m), 1597 (m), 1573 (m), 1455 (m), 1285 (m), 1071 (m), 742 (m) cm^{-1}.

S4.10. *N*-Isopropylbenzene-1,2-diamine (**14j**)

The standard procedure 2 was followed by use of *o*-phenylenediamine (**12**, 508 mg, 4.69 mmol, 1.0 equiv), acetone (**13j**, 68.1 mg, 1.17 mmol, 0.25 equiv), $NaBH_4$ (43.4 mg, 1.17 mmol, 0.25 equiv), and MeOH (7.0 mL). After workup, the residue was purified by use of silica gel column chromatography (10% EtOAc in hexanes as the eluent) to give the desired *N*-alkylated-1,2-diamine **14j** (564 mg, 3.75 mmol) in 80% overall yield as a purple liquid: ^{1}H NMR ($CDCl_3$, 400 MHz) δ 6.93–6.89 (m, 1 H, ArH), 6.78–6.75 (m, 3 H, 3 × ArH), 3.69–3.65 (m, 1 H, $NCHMe_2$), 1.31 (d, J = 6.4 Hz, 6 H, 2 × CH_3). The spectroscopic data was in accordance with those reported in the literature [78].

S4.11. *N*-Cyclopentylbenzene-1,2-diamine (**14k**)

The standard procedure 2 was followed by use of *o*-phenylenediamine (**12**, 511 mg, 4.71mmol, 1.0 equiv), cyclopentanone (**13k**, 99.1 mg, 1.17 mmol, 0.25 equiv), $NaBH_4$ (43.4 mg, 1.17 mmol, 0.25 equiv), and MeOH (7.5 mL). After workup, the residue was purified by use of silica gel column chromatography (10% EtOAc in hexanes as the eluent) to give the desired *N*-alkylated-1,2-diamine **14k** (649 mg, 3.68 mmol) in 78% overall yield as a purple liquid: ^{1}H NMR ($CDCl_3$, 400 MHz) δ 6.86–6.81 (m, 1 H, ArH), 6.73–6.66 (m, 3 H, 3 × ArH), 3.82–3.78 (m, 1 H, *H*C(CH_2)$_2$), 2.12–2.01 (m, 2 H, CH_2), 1.73–1.69 (m, 2 H, CH_2), 1.68–1.61 (m, 2 H, CH_2), 1.59–1.52 (m, 2 H, CH_2); ^{13}C NMR ($CDCl_3$, 100 MHz) δ 137.5, 134.1, 120.5, 118.1, 116.3, 112.5, 54.4 (CNPh), 33.6 (2 × CH_2), 24.2 (2 × CH_2); IR (neat) 3333 (m, NH), 2955 (m), 2867 (m), 1594 (m), 1505 (s), 1451 (m), 1269 (m), 739 (s) cm^{-1}.

S4.12. *N*-Cyclohexylbenzene-1,2-diamine (**14l**)

The standard procedure 2 was followed by use of *o*-phenylenediamine (**12**, 505 mg, 4.66 mmol, 1.0 equiv), cyclohexanone (**13l**, 118 mg, 1.16 mmol, 0.25 equiv), $NaBH_4$ (43.1 mg, 1.16 mmol, 0.25 equiv), and MeOH (7.5 mL). After workup, the residue was purified by use of silica gel column chromatography (5% EtOAc in hexanes as the eluent) to give the desired *N*-alkylated-1,2-diamine **14l** (701 mg, 3.68 mmol) in 79% overall yield as a purple liquid: ^{1}H NMR ($CDCl_3$, 400 MHz) δ 6.85–6.81 (m, 1 H, ArH), 6.74–6.65 (m, 3 H, 3 × ArH), 3.29–3.22 (m, 1 H, *H*C(CH_2)$_2$),

2.11–2.07 (m, 2 H, CH_2), 1.83–1.78 (m, 2 H, CH_2), 1.72–1.67 (m, 1 H, *H*CH), 1.46–1.36 (m, 2 H, CH_2), 1.33–1.21 (m, 3 H, CH_2 + HC*H*). The spectroscopic data was in accordance with those reported in the literature [78].

*S5. Preparation of Diamide (**15**) (Standard Procedure 3)*

To a solution of bisbenzofuroic acid **11** (1.0 equiv) in DMF (2.5−5.0 mL) were added amines **14** (2.1 equiv), hydroxybenzotriazole (HOBt, 2.4 equiv), *N*-methylmorpholine (NMM, 4.0 equiv), and 1-ethyl-3-(3-dimethylaminopropyl)carbodiimide (EDCI, 2.4 equiv) at room temperature under nitrogen atmosphere. The reaction mixture was stirred at room temperature for 16 h. The residue was diluted with EtOAc (50 mL), washed with H_2O (3 × 20 mL) and then brine (3 × 20 mL), dried over $MgSO_4$ (s), filtered, and concentrated under reduced pressure. The residue was purified by use of silica gel column chromatography (a mixture of EtOAc and hexanes) to give diamide **15**.

S5.1. 5,5'-Methylenebis(*N*-[2-(benzylamino)phenyl]benzofuran-2-carboxamide) (**15a**)

The standard procedure 3 was followed by use of bisfuroic acid **11** (64.2 mg, 0.191 mmol, 1.0 equiv), diamine **14a** (79.4 mg, 0.401 mmol, 2.1 equiv), HOBt (70.1 mg, 0.458 mmol, 2.4 equiv), NMM (75.5 mg, 0.764 mmol, 4.0 equiv), and EDCI (71.2 mg, 0.458 mmol, 2.4 equiv) in DMF (2.5 mL). After workup, the residue was purified by use of silica gel column chromatography (25% EtOAc in hexanes as the eluent) to give the desired diamide **15a** (97.3 mg, 0.141 mmol) in 74% overall yield as white solids: mp (recrystallized from EtOH) 172.6–174.2 °C; ^{1}H NMR ($CDCl_3$, 400 MHz) δ 8.32 (s, 2 H, 2 × NH), 7.49–7.39 (m, 12 H, 12 ×ArH), 7.33–7.22 (m, 8 H, 8 x ArH), 7.12 (t, J = 7.8 Hz, 2 H, 2 × ArH), 6.84–6.78 (m, 4 H, 4 x ArH), 4.36 (s, 4 H, 2 × CH_2Ph), 4.17 (s, 2 H, ArCH_2Ar); ^{13}C NMR ($CDCl_3$, 100 MHz) δ 157.2 (C=O), 153.6, 148.7, 141.9, 139.0, 136.9, 128.6, 128.5, 127.9, 127.5, 127.3, 127.2, 125.1, 123.6, 122.6, 118.8, 114.2, 111.8, 111.4, 48.0 (PhCH_2), 41.3 (ArCH_2Ar); IR (neat) 3382 (m, NH), 2958 (m), 2855 (m), 1667 (s, C=O), 1506 (s), 1455 (w), 1310 (m), 1191 (s), 1081 (m), 743 (m) cm^{-1}; ESIMS calcd for ($C_{45}H_{36}N_4O_4$): 696.2737; found 696.2738.

S5.2. 5,5'-Methylenebis(*N*-[2-(*p*-methylbenzylamino)phenyl]benzofuran-2-carboxamide) (**15b**)

The standard procedure 3 was followed by use of bisfuroic acid **11** (80.2 mg, 0.238 mmol, 1.0 equiv), diamine **14b** (106 mg, 0.501 mmol, 2.1 equiv), HOBt (87.4 mg, 0.571 mmol, 2.4 equiv), NMM (93.1 mg, 0.952 mmol, 4.0 equiv), and EDCI (88.6 mg, 0.571 mmol, 2.4 equiv) in DMF (3.0 mL). After workup, the residue was purified by use of silica gel column chromatography (25% EtOAc in hexanes as the eluent) to give the desired diamide **15b** (131 mg, 0.181 mmol) in 76% overall yield as pale yellow solids: mp (recrystallized from EtOH) 176.8–178.6 °C; ^{1}H NMR ($CDCl_3$, 400 MHz) δ 8.33 (s, 2 H, 2 × NH), 7.49−7.39 (m, 8 H, 8 ×ArH), 7.26–7.26 (m, 6 H, 6 × ArH), 7.14–7.11 (m, 6 H, 6 × ArH), 6.84–6.78 (m, 4 H, 4 × ArH), 4.30 (s, 4 H, 2 × CH_2Ph), 4.17 (s, 2 H, ArCH_2Ar), 2.30 (s, 6 H, 2 × CH_3); ^{13}C NMR ($CDCl_3$, 100 MHz) δ 157.2 (C=O), 153.6, 148.7, 142.0, 136.9, 136.8, 135.9, 129.2, 128.4, 127.8, 127.4, 127.3, 125.1, 123.6, 122.5, 118.7, 114.1, 111.8, 111.3, 48.3 (PhCH_2), 41.4 (ArCH_2Ar), 21.0 (CH_3); IR (neat) 3381 (m, NH), 2926 (m), 2856 (m), 1668 (s, C=O), 1553 (s), 1455 (w), 1310 (m), 1191 (s), 743 (m) cm^{-1}; ESIMS calcd for ($C_{47}H_{40}N_4O_4$): 724.3050; found 724.3048.

S5.3. 5,5'-Methylenebis(*N*-[2-(*m*-methoxybenzylamino)phenyl]benzofuran-2-carboxamide) (**15c**)

The standard procedure 3 was followed by use of bisfuroic acid **11** (81.2 mg, 0.241 mmol, 1.0 equiv), diamine **14c** (115 mg, 0.507 mmol, 2.1 equiv), HOBt (85.6 mg, 0.578 mmol, 2.4 equiv), NMM (97.5 mg, 0.964 mmol, 4.0 equiv), and EDCI (89.7 mg, 0.578 mmol, 2.4 equiv) in DMF (3.0 mL). After workup, the residue was purified by use of silica gel column chromatography (25% EtOAc in hexanes as the eluent) to give the desired diamide **15c** (136 mg, 0.179 mmol) in 75% overall yield as white solids: mp (recrystallized from EtOH) 175.0–177.2 °C; ^{1}H NMR ($CDCl_3$, 400 MHz) δ 8.29 (s, 2 H, 2 × NH), 7.49–7.44 (m, 8 H, 8 ×ArH), 7.28–7.21 (m, 6 H, 6 × ArH), 7.12 (t, J = 7.6 Hz, 2 H, 2 × ArH), 6.97 (d, J = 7.6 Hz, 4 H, 4 × ArH), 6.82 (t, J = 7.6 Hz, 2 H, 2 × ArH), 6.78 (d, J = 8.8 Hz, 2 H, 2 × ArH), 4.34 (s, 4 H, 2 × CH_2Ph), 4.18 (s, 2 H, ArCH_2Ar), 3.76 (s, 6 H, 2 × OCH_3); ^{13}C NMR ($CDCl_3$, 100 MHz) δ 159.7, 157.2 (C=O), 153.6, 148.6, 141.9, 140.7, 136.8, 129.5, 128.3, 127.8, 127.4, 125.1, 123.5, 122.4, 119.5, 118.6, 113.9, 112.9, 112.3, 111.7, 111.2, 55.1 (OCH_3), 48.3 (PhCH_2), 41.3 (ArCH_2Ar); IR (neat) 3326 (m, NH), 2957(m), 2870 (m), 1667 (s, C=O), 1605 (s), 1454 (w), 1320 (m), 1195 (s), 745 (m) cm^{-1}; ESIMS calcd for ($C_{47}H_{40}N_4O_6$ + H): 757.3026; found 757.3021.

S5.4. 5,5'-Methylenebis(*N*-[2-(*p*-methoxybenzylamino)phenyl]benzofuran-2-carboxamide) (**15d**)

The standard procedure 3 was followed by use of bisfuroic acid **11** (85.1 mg, 0.253 mmol, 1.0 equiv), diamine **14d** (121 mg, 0.531 mmol, 2.1 equiv), HOBt (92.9 mg, 0.607 mmol, 2.4 equiv), NMM (102 mg, 1.01 mmol, 4.0 equiv), and EDCI (89.7 mg, 0.607 mmol, 2.4 equiv) in DMF (4.0 mL). After workup, the residue was purified by use of silica gel column chromatography (25% EtOAc in hexanes as the eluent) to give the desired diamide **15d** (155 mg, 0.205 mmol) in 81% overall yield as white solids: mp (recrystallized from EtOH) 174.6–176.4 °C; ^{1}H NMR ($CDCl_3$, 400 MHz) δ 8.37 (s, 2 H, 2 × NH), 7.47 (d, J = 7.8 Hz, 2 H, 2 ×ArH), 7.44–7.39 (m, 6 H, 6 × ArH), 7.30 (d, J = 7.8 Hz, 4 H, 4 × ArH), 7.25 (d, J = 7.4 Hz, 2 H, 2 × ArH), 7.14–7.10 (m, 2 H, 2 × ArH), 6.84 (d, J = 7.4 Hz, 2 H, 2 × ArH), 6.81–6.78 (m, 6 H, 6 × ArH), 4.26 (s, 4 H, 2 × CH_2Ph), 4.15 (s, 2 H, ArCH_2Ar), 3.74 (s, 6 H, 2 × OCH_3); ^{13}C NMR ($CDCl_3$, 100 MHz) δ 158.7, 157.2 (C=O), 153.6, 148.6, 141.9, 136.8, 130.9, 128.6, 128.3, 127.8, 127.4, 125.0, 123.6, 122.5, 118.6, 114.1, 113.9, 111.7, 111.2, 55.1 (OCH_3), 47.9 (PhCH_2), 41.3 (ArCH_2Ar); IR (neat) 3326 (m, NH), 2957 (m), 2925 (m), 2855 (m), 1667 (s, C=O), 1517 (s), 1453 (w), 1265 (m), 1195 (s), 746 (m) cm^{-1}; ESIMS calcd for ($C_{47}H_{40}N_4O_6$ + H): 757.3026; found 757.3020.

S5.5. 5,5'-Methylenebis(*N*-[2-(*o*-fluorobenzylamino)phenyl]benzofuran-2-carboxamide) (**15e**)

The standard procedure 3 was followed by use of bisfuroic acid **11** (81.2 mg, 0.241 mmol, 1.0 equiv), diamine **14e** (109 mg, 0.505 mmol, 2.1 equiv), HOBt (88.5 mg, 0.578 mmol, 2.4 equiv), NMM (97.5 mg, 1.01 mmol, 4.0 equiv), and EDCI (89.7 mg, 0.578 mmol, 2.4 equiv) in DMF (4.0 mL). After workup, the residue was purified by use of silica gel column chromatography (25% EtOAc in hexanes as the eluent) to give the desired diamide **15e** (137 mg, 0.187 mmol) in 78% overall yield as yellow solids: mp (recrystallized from EtOH) 177.2–178.8 °C; ^{1}H NMR ($CDCl_3$, 400 MHz) δ 8.35 (s, 2 H, 2 × NH), 7.49 (d, J = 7.4 Hz, 2 H, 2 × ArH), 7.45–7.38 (m, 8 H, 8 × ArH), 7.28 (d, J = 7.4 Hz, 2 H, 2 × ArH), 7.22–7.18 (m, 2 H, 2 × ArH), 7.13–7.00 (m, 6 H, 6 × ArH), 6.83 (t, J = 8.0 Hz, 2 H, 2 × ArH), 6.78 (d, J = 8.0 Hz, 2 H, 2 × ArH), 4.42 (s, 4 H, 2 × CH_2Ph), 4.18 (s, 2 H, ArCH_2Ar); ^{13}C NMR ($CDCl_3$, 100 MHz) δ 160.8 (d, J_{CF} = 244 Hz), 157.2

(C=O), 153.7, 148.7, 141.6, 136.9, 129.2 (d, J_{CF} = 3.7 Hz), 128.6 (d, J_{CF} = 8.3 Hz), 128.5, 127.9, 127.5, 125.9 (d, J_{CF} = 14.4 Hz), 125.0, 124.2 (d, J_{CF} = 3.0 Hz), 124.0, 122.6, 119.2, 115.2 (d, J_{CF} = 21.3 Hz), 114.5, 111.8, 111.4, 42.2 (d, J_{CF} = 4.6 Hz, $PhCH_2$), 41.4 ($ArCH_2Ar$); IR (neat) 3358 (m, NH), 2977 (m), 2931 (w), 1699 (s, C=O), 1516 (s), 1456 (w), 1367 (m), 1163 (s), 1052 (m), 747 (m) cm^{-1};ESIMS calcd for ($C_{45}H_{34}F_2N_4O_4$ + H): 733.2626; found 733.2621.

S5.6. 5,5'-Methylenebis(*N*-[2-(*p*-fluorobenzylamino)phenyl]benzofuran-2-carboxamide) (**15f**)

The standard procedure 3 was followed by use of bisfuroic acid **11** (84.2 mg, 0.251 mmol, 1.0 equiv), diamine **14f** (112 mg, 0.525 mmol, 2.1 equiv), HOBt (92.2 mg, 0.602 mmol, 2.4 equiv), NMM (101 mg, 1.04 mmol, 4.0 equiv), and EDCI (93.4 mg, 0.602 mmol, 2.4 equiv) in DMF (5.0 mL). After workup, the residue was purified by use of silica gel column chromatography (20% EtOAc in hexanes as the eluent) to give the desired diamide **15f** (135 mg, 0.184 mmol) in 74% overall yield as yellow solids: mp (recrystallized from EtOH) 180.2–182.4 °C; ^{1}H NMR ($CDCl_3$, 400 MHz) δ 8.31 (s, 2 H, 2 × NH), 7.47–7.42 (m, 8 H, 8 × ArH), 7.36–7.26 (m, 6 H, 6 × ArH), 7.12 (t, J = 7.8 Hz, 2 H, 2 × ArH), 6.99 (t, J = 8.4 Hz, 4 H, 4 × ArH), 6.82 (t, J = 7.8 Hz, 2 H, 2 × ArH), 6.74 (d, J = 8.4 Hz, 2 H, 2 × ArH), 4.44 (s, 4 H, 2 × CH_2Ph), 4.17 (s, 2 H, $ArCH_2Ar$); ^{13}C NMR ($CDCl_3$, 100 MHz) δ 161.9 (d, J_{CF} = 244 Hz), 157.2 (C=O), 153.7, 148.6, 141.7, 137.5, 135.1 (d, J_{CF} = 3.1 Hz), 129.2 (d, J_{CF} = 8.4 Hz), 128.5, 127.9, 127.6, 125.2, 123.6, 122.7, 118.9, 115.5 (d, J_{CF} = 21.4 Hz), 114.1, 111.9, 111.5, 47.9 ($PhCH_2$), 41.7 ($ArCH_2Ar$); IR (neat) 3382 (m, NH), 2958 (m), 2855 (m), 1667 (s, C=O), 1506 (s), 1455 (w), 1310 (m), 1191 (s), 1081 (m), 743 (m) cm^{-1}; ESIMS calcd for ($C_{45}H_{34}F_2N_4O_4$ + H): 733.2626; found 733.2624.

S5.7. 5,5'-Methylenebis(*N*-[2-(*o*-chlorobenzylamino)phenyl]benzofuran-2-carboxamide) (**15g**)

The standard procedure 3 was followed by use of bisfuroic acid **11** (84.3 mg, 0.251 mmol, 1.0 equiv), diamine **14g** (122mg, 0.526 mmol, 2.1 equiv), HOBt (93.5 mg, 0.602 mmol, 2.4 equiv), NMM (101 mg, 1.01 mmol, 4.0 equiv), and EDCI (93.5 mg, 0.602 mmol, 2.4 equiv) in DMF (4.5mL). After workup, the residue was purified by use of silica gel column chromatography (25% EtOAc in hexanes as the eluent) to give the desired diamide **15g** (147 mg, 0.192 mmol) in 77% overall yield as white solids: mp (recrystallized from EtOH) 173.8–175.6 °C; ^{1}H NMR ($CDCl_3$, 400 MHz) δ 8.35 (s, 2 H, 2 × NH), 7.50–7.42 (m, 10 H, 10 × ArH), 7.36–7.34 (m, 2 H, 2 × ArH), 7.28 (d, J = 8.4 Hz, 2 H, 2 × ArH), 7.19–7.17 (m, 4 H, 4 × ArH), 7.10 (t, J = 8.0 Hz, 2 H, 2 × ArH), 6.82 (t, J = 7.6 Hz, 2 H, 2 × ArH), 6.68 (d, J = 8.0 Hz, 2 H, 2 × ArH), 4.45 (s, 4 H, 2 × CH_2Ph), 4.18 (s, 2 H, $ArCH_2Ar$); ^{13}C NMR ($CDCl_3$, 100 MHz) δ 157.2 (C=O), 153.7, 148.7, 141.6, 136.9, 136.2, 133.2, 129.4, 128.8, 128.5, 127.9, 127.6, 127.0, 125.2, 123.8, 122.6, 119.2, 119.0, 114.4, 111.8, 111.4, 46.1 (CH_2Ph), 41.5 ($ArCH_2Ar$); IR (neat) 3316 (m, NH), 2916 (m), 2850 (m), 1667 (s, C=O), 1506 (s), 1450 (m), 1317 (w), 1195 (m), 741 (m) cm^{-1}; ESIMS calcd for ($C_{45}H_{34}Cl_2N_4O_4$ + H): 765.2035; found 765.2033.

S5.8. 5,5'-Methylenebis(*N*-[2-(*p*-chlorobenzylamino)phenyl]benzofuran-2-carboxamide) (**15h**)

The standard procedure 3 was followed by use of bisfuroic acid **11** (81.3 mg, 0.241 mmol, 1.0 equiv), diamine **14h** (118 mg, 0.507 mmol, 2.1 equiv), HOBt (88.5 mg, 0.602 mmol, 2.4 equiv), NMM (101 mg, 0.964 mmol, 4.0 equiv), and EDCI (89.7 mg, 0.578 mmol, 2.4 equiv) in DMF (4.5 mL). After workup, the residue was purified by use of silica gel column chromatography (20%

EtOAc in hexanes as the eluent) to give the desired diamide **15h** (145 mg, 0.190 mmol) in 79% overall yield as white solids: mp (recrystallized from EtOH) 182.2–184.4 °C; ^{1}H NMR (DMSO-d_6, 400 MHz) δ 7.67 (s, 2 H, 2 × ArH), 7.62 (d, J = 8.4 Hz, 4 H, 4 × ArH), 7.42–7.33 (m, 10 H, 10 × ArH), 7.14 (d, J = 7.6 Hz, 2 H, 2 × ArH), 6.98 (t, J = 7.6 Hz, 2 H, 2 × ArH), 6.58 (t, J = 8.0 Hz, 2 H, 2 × ArH), 6.46 (d, J = 8.0 Hz, 2 H, 2 × ArH), 5.89 (s, 2 H, 2 × NH), 4.34 (s, 4 H, 2 × CH_2Ph), 4.20 (s, 2 H, ArCH_2Ar); ^{13}C NMR (DMSO-d_6, 100 MHz) δ 157.4 (C=O), 153.1, 149.5, 143.2, 139.2, 137.1, 130.9, 128.7, 128.1, 128.0, 127.5, 127.4, 127.2, 122.3, 122.2, 115.6, 111.7, 111.2, 110.1, 45.3 (CH_2Ph), 40.5 (ArCH_2Ar); IR (neat) 3317 (m, NH), 2916 (m), 2850 (m), 1667 (s, C=O), 1589 (s), 1451 (m), 1265 (m), 1195 (m), 743 (m) cm^{-1}; ESIMS calcd for ($C_{45}H_{34}Cl_2N_4O_4$ + H): 765.2035; found 765.2029.

S5.9. 5,5'-Methylenebis(*N*-[2-(*m*-bromobenzylamino)phenyl]benzofuran-2-carboxamide) (**15i**)

The standard procedure 3 was followed by use of bisfuroic acid **11** (61.2 mg, 0.182 mmol, 1.0 equiv), diamine **14i** (118 mg, 0.382 mmol, 2.1 equiv), HOBt (66.8 mg, 0.436 mmol, 2.4 equiv), NMM (73.6 mg, 0.728 mmol, 4.0 equiv), and EDCI (67.8 mg, 0.436 mmol, 2.4 equiv) in DMF (4.5 mL). After workup, the residue was purified by use of silica gel column chromatography (30% EtOAc in hexanes as the eluent) to give the desired diamide **15i** (124 mg, 0.145 mmol) in 80% overall yield as yellow solids: mp (recrystallized from EtOH) 185.6–187.4 °C; ^{1}H NMR ($CDCl_3$, 400 MHz) δ 8.30 (s, 2 H, 2 × NH), 7.55 (s, 2 H, 2 × ArH), 7.50–7.43 (m, 8 H, 8 × ArH), 7.37–7.27 (m, 6 H, 6 × ArH), 7.17 (t, J = 7.6 Hz, 2 H, 2 × ArH), 7.11 (t, J = 8.2 Hz, 2 H, 2 × ArH), 6.83 (t, J = 7.6 Hz, 2 H, 2 × ArH), 6.71 (d, J = 8.2 Hz, 2 H, 2 × ArH), 4.34 (s, 4 H, 2 × CH_2Ph), 4.18 (s, 2 H, ArCH_2Ar); ^{13}C NMR ($CDCl_3$, 100 MHz) δ 157.2 (C=O), 153.7, 148.6, 141.7, 141.6, 136.9, 130.3, 130.2, 130.1, 128.5, 127.9, 127.6, 125.8, 125.2, 123.6, 122.7, 122.6, 119.0, 114.1, 111.8, 111.5, 47.9 (CH_2Ph), 41.5 (ArCH_2Ar); IR (neat) 3373 (m, NH), 2926 (m), 2854 (m), 1667 (s, C=O), 1582 (s), 1455 (m), 1315 (w), 1195 (m), 742 (m) cm^{-1}; ESIMS calcd for ($C_{45}H_{34}Br_2N_4O_4$ + H): 853.1025; found 853.1019.

S5.10. 5,5'-Methylenebis(*N*-[2-(isopropylamino)phenyl]benzofuran-2-carboxamide) (**15j**)

The standard procedure 3 was followed by use of bisfuroic acid **11** (71.1 mg, 0.211 mmol, 1.0 equiv), diamine **14j** (66.7 mg, 0.444 mmol, 2.1 equiv), HOBt (77.5 mg, 0.506 mmol, 2.4 equiv), NMM (85.3 mg, 0.844 mmol, 4.0 equiv), and EDCI (78.6 mg, 0.506 mmol, 2.4 equiv) in DMF (3.0 mL). After workup, the residue was purified by use of silica gel column chromatography (20% EtOAc in hexanes as the eluent) to give the desired diamide **15j** (104 mg, 0.172 mmol) in 82% overall yield as off-white solids: mp (recrystallized from EtOH) 156.6–158.4 °C; ^{1}H NMR ($CDCl_3$, 400 MHz) δ 8.46 (s, 2 H, 2 × NH), 7.62 (d, J = 7.6 Hz, 2 H, 2 × ArH), 7.48–7.45 (m, 6 H, 6 × ArH), 7.28 (d, J = 8.2 Hz, 2 H, 2 × ArH), 7.13 (t, J = 8.2 Hz, 2 H, 2 × ArH), 6.84 (d, J = 7.6 Hz, 4 H, 4 × ArH), 4.18 (s, 2 H, ArCH_2Ar), 3.57–3.51 (m, 2 H, 2 × HCMe_2), 1.22 (d, J = 6.0 Hz, 12 H, 4 × CH_3); ^{13}C NMR ($CDCl_3$, 100 MHz) δ 157.0 (C=O), 153.6, 148.9, 140.6, 136.9, 128.3, 127.9, 126.8, 125.1, 124.5, 122.5, 119.2, 115.9, 111.8, 111.1, 45.4 ($CHMe_2$), 41.4 (ArCH_2Ar), 22.9 (CH_3); IR (neat) 3299 (m, NH), 2965 (m), 2926 (m), 1660 (s, C=O), 1582 (s), 1455 (m), 1306 (w), 1266 (m), 743 (m) cm^{-1}; ESIMS calcd for ($C_{37}H_{36}N_4O_4$ + H): 601.2815; found 601.2816.

S5.11. 5,5'-Methylenebis(*N*-[2-(cyclopentylamino)phenyl]benzofuran-2-carboxamide) (**15k**)

The standard procedure 3 was followed by use of bisfuroic acid **11** (75.2 mg, 0.223 mmol, 1.0 equiv), diamine **14k** (82.7 mg, 0.469 mmol, 2.1 equiv), HOBt (81.9 mg, 0.535 mmol, 2.4 equiv), NMM (90.2 mg, 0.892 mmol, 4.0 equiv), and EDCI (83.1 mg, 0.535 mmol, 2.4 equiv) in DMF (4.0 mL). After workup, the residue was purified by use of silica gel column chromatography (15% EtOAc in hexanes as the eluent) to give the desired diamide **15k** (118 mg, 0.181 mmol) in 81% overall yield as off-white solids: mp (recrystallized from EtOH) 165.4–167.2 °C; ^{1}H NMR ($CDCl_3$, 400 MHz) δ 8.32 (s, 2 H, 2 × NH), 7.53–7.45 (m, 8 H, 8 × ArH), 7.28 (dd, *J* = 8.4, 1.6 Hz, 2 H, 2 × ArH), 7.16–7.12 (m, 2 H, 2 × ArH), 6.87–6.79 (m, 4 H, 4 × ArH), 4.18 (s, 2 H, $ArCH_2Ar$) 3.81–3.75 (m, 2 H, 2 × *H*C(CH_2)$_2$), 2.04–1.97 (m, 4 H, 2 × CH_2), 1.73–1.64 (m, 4 H, 2 × CH_2), 1.62–1.58 (m, 4 H, 2 × CH_2), 1.56–1.46 (m, 4 H, 2 × CH_2); ^{13}C NMR ($CDCl_3$, 100 MHz) δ 157.1 (C=O), 153.7, 148.9, 141.6, 136.9, 128.4, 127.9, 127.1, 124.6, 124.5, 122.5, 118.6, 115.2, 111.8, 111.2, 55.2 (*C*H(CH_2)$_2$), 41.5 ($ArCH_2Ar$), 33.5 (CH_2), 24.1 (CH_2); IR (neat) 3285 (m, NH), 2954 (m), 2867 (m), 1659 (s, C=O), 1582 (s), 1453 (m), 1311 (w), 1266 (m), 743 (m) cm^{-1}; ESIMS calcd for ($C_{41}H_{40}N_4O_4$ + H): 653.3128; found 653.3125.

S5.12. 5,5'-Methylenebis(*N*-[2-(cyclohexylamino)phenyl]benzofuran-2-carboxamide) (**15l**)

The standard procedure 3 was followed by use of bisfuroic acid **11** (78.3 mg, 0.232 mmol, 1.0 equiv), diamine **14l** (92.6 mg, 0.487 mmol, 2.1 equiv), HOBt (85.3 mg, 0.556 mmol, 2.4 equiv), NMM (93.8 mg, 0.928 mmol, 4.0 equiv), and EDCI (86.3 mg, 0.556 mmol, 2.4 equiv) in DMF (4.0 mL). After workup, the residue was purified by use of silica gel column chromatography (15% EtOAc in hexanes as the eluent) to give the desired diamide **15l** (118 mg, 0.181 mmol) in 83% overall yield as off-white solids: mp (recrystallized from EtOH) 168.6–170.8 °C; ^{1}H NMR ($CDCl_3$, 400 MHz) δ 8.48 (s, 2 H, 2 × NH), 7.62 (d, *J* = 7.6 Hz, 2 H, 2 × ArH), 7.57–7.47 (m, 6 H, 6 × ArH), 7.28 (d, *J* = 8.8 Hz, 2 H, 2 × ArH), 7.23–7.09 (m, 2 H, 2 × ArH), 6.86–6.80 (m, 4 H, 4 × ArH), 4.18 (s, 2 H, $ArCH_2Ar$) 3.20–3.16 (m, 2 H, 2 × *H*C(CH_2)$_2$), 2.06–2.03 (m, 4 H, 2 × CH_2), 1.76–1.73 (m, 4 H, 2 × CH_2), 1.63–1.60 (m, 2 H, 2 × *H*CH), 1.37–1.18 (m, 10 H, 4 × CH_2 + 2 × HC*H*); ^{13}C NMR ($CDCl_3$, 100 MHz) δ 156.9 (C=O), 153.6, 148.9, 140.4, 136.9, 128.3, 127.9, 126.7, 125.0, 124.4, 122.5, 119.1, 115.9, 111.7, 111.1, 52.8 (*C*H(CH_2)$_2$), 41.4 ($ArCH_2Ar$), 33.4 (CH_2), 25.8 (CH_2), 24.8 (CH_2); IR (neat) 3290 (m, NH), 2954 (m), 2868 (m), 1660 (s, C=O), 1605 (s), 1453 (m), 1311 (w), 1194 (m), 743 (m) cm^{-1}; ESIMS calcd for ($C_{43}H_{44}N_4O_4$ + H): 681.3440; found 681.3442.

References

72. Sheng, C.; Che, X.; Wang, W.; Wang, S.; Cao, Y.; Yao, J.; Miao, Z.; Zhang, W. Structure-based Design, Synthesis, and Antifungal Activity of New Triazole Derivatives. *Chem. Biol. Drug Des.* **2013**, *78*, 309–313.

73. Mulliez, M. Etude D'Un Schema de Synthese Peptidique Intramoleculaire a L'Aide de Derives du Phosphore Tetraedrique. *Tetrahedron* **1981**, *37*, 2027–2041.
74. Pilkington, L.I.; Haverkate, N.A.; van Rensburg, M.; Reynisson, J.; Leung, E.; Barker, D. Synthesis of 3-Amino-2-carboxamide Tetrahydropyrrolo[2,3-*b*]quinolines. *Synlett* **2016**, *27*, 2811–2814.
75. Sykes, B.M.; Atwell, G.J.; Hogg, A.; Wilson, W.R.; O'Connor, C.J.; Denny, W.A. *N*-Substituted 2-(2,6-Dinitrophenylamino)propanamides: Novel Prodrugs that Release a Primary Amine via Nitroreduction and Intramolecular Cyclization. *J. Med. Chem.* **1999**, *42*, 346–355.
76. Wei, C.; Wang, C.E.; Hwan, K.M.; Ronald, K.R.; Le, D.T.; Lew, A.; Nuss, J.M.; Wei, X. P70s6 Kinase Modulators and Method of Use. PCT Int Appl: WO2005/39506, A2, May 6, 2005.
77. Banik, A.; Ahmed, J.; Sil, S.; Mandal, S.K. Mimicking Transition Metals in Borrowing Hydrogen from Alcohols. *Chem. Sci.* **2021**, *12*, 8353–8361.
78. Zhang, S.; Yi, D.; Li, G.; Li, L.; Zhao, G.; Tang, Z. Biomimetic Alloxan-catalyzed Intramolecular Redox Reaction with O_2: One-Pot Atom-Economic Synthesis of Sulfinyl-Functionalized Benzimidazoles. *Tetrahedron Lett.* **2021**, *62*, 152688.
79. Kallmeier, F.; Fertig, R.; Irrgang, T.; Kempe, R. Chromium-catalyzed Alkylation of Amines by Alcohols. *Angew. Chem. Int. Ed.* **2020**, *59*, 11789–11793.
80. Ryu, H.J.; Yang, S.J.; Lee, G.H.; Gong, Y.D. Construction of Druglike 2-Amido Benzo[*d*]imidazole Analogues via Desulfurative Cyclization of Thiourea Intermediate Resin on Solid-phase. *ACS Comb. Sci.* **2018**, *20*, 282−291.
81. Tian, J.; Vandermosten, L.; Peigneur, S.; Moreels, L.; Rozenski, J.; Tytgat, J.; Herdewijn, P.; den Steen, P.E.V.; De Jonghe, S. Astemizole Analogues with Reduced hERG Inhibition as Potent Antimalarial Compounds. *Bioorg. Med. Chem.* **2017**, *25*, 6332–6344.
82. Heydari, A.; Khaksar, S.; Esfandyari, M.; Tajbakhsh, M. A Novel One-Pot Reductive Amination of Aldehydes and Ketones with Lithium Perchlorate and Zirconium Borohydride–Piperazine Complexes. *Tetrahedron* **2007**, *63*, 3363–3366.

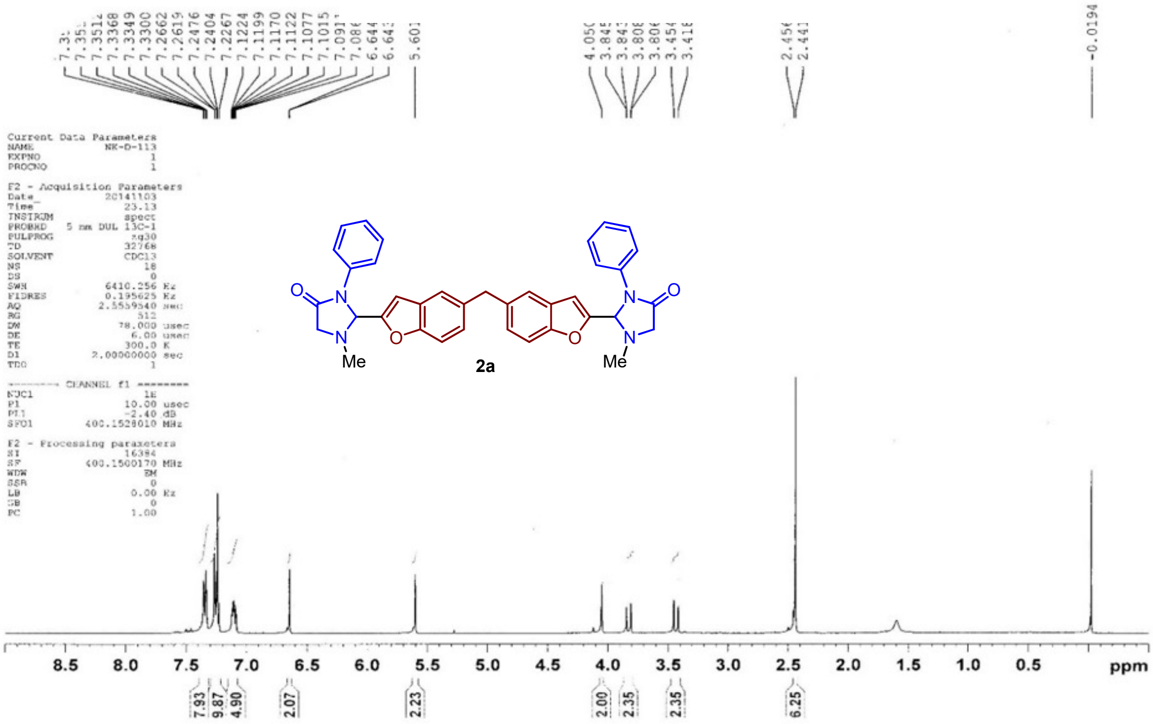

^{1}H NMR spectrum of compound **2a**

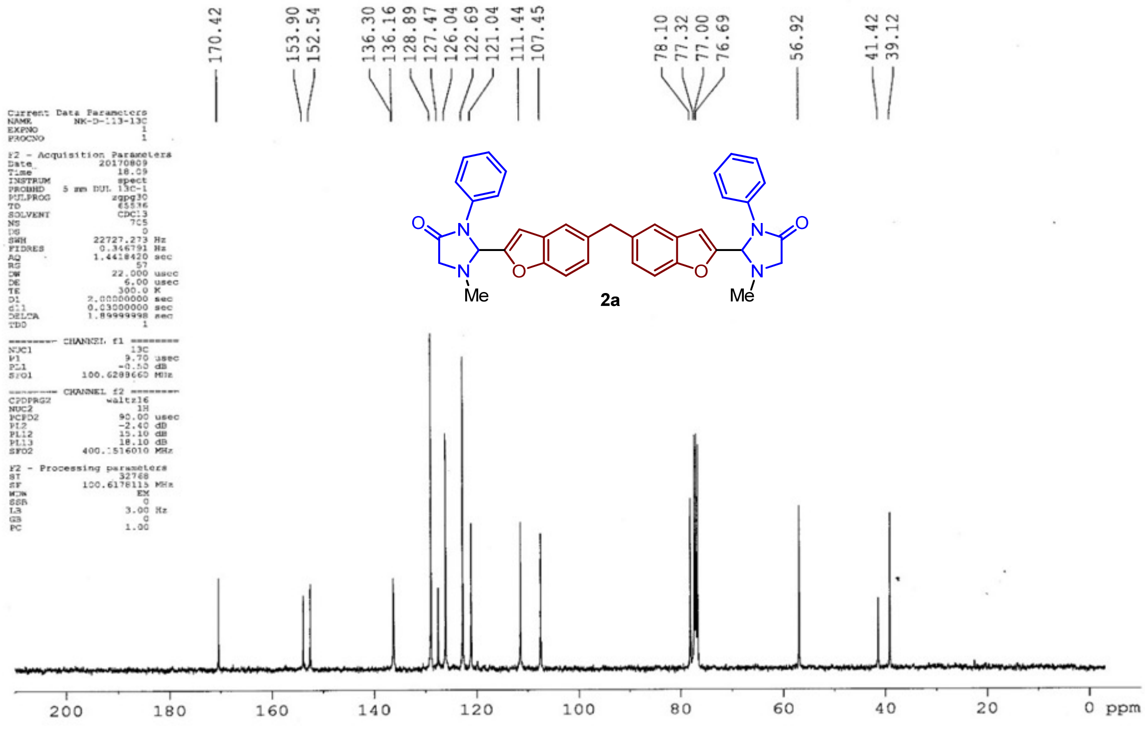

^{13}C NMR spectrum of compound **2a**

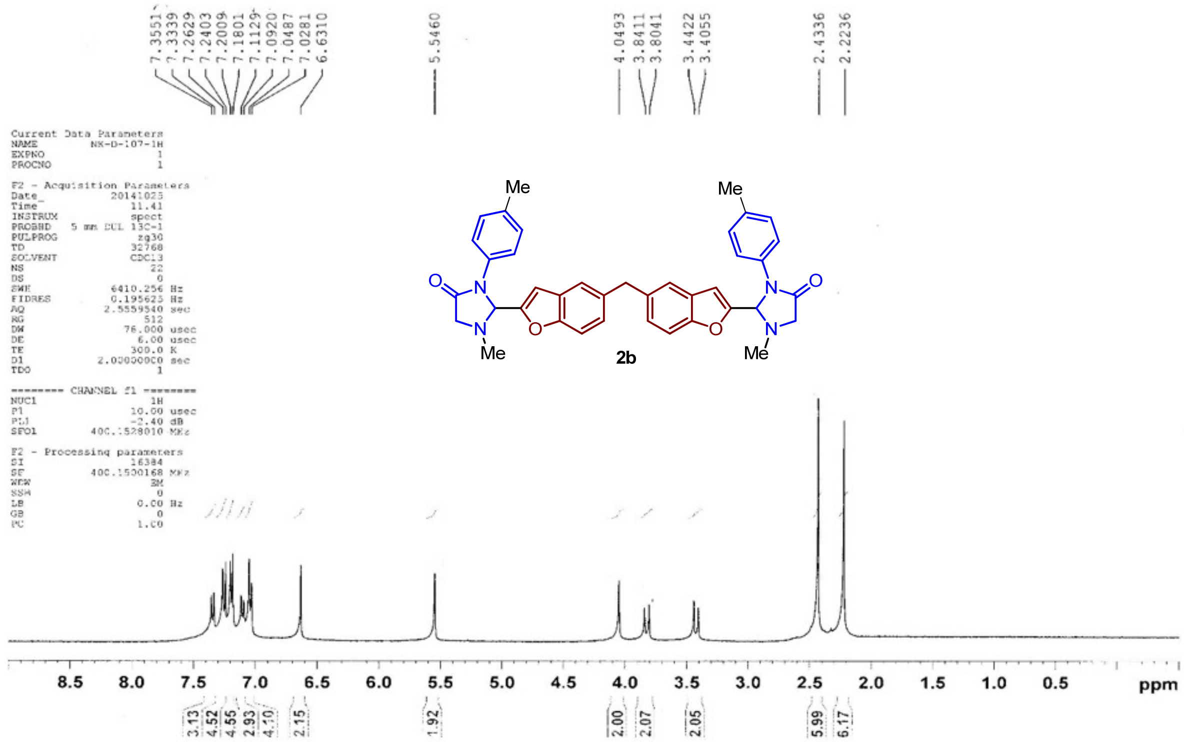

^{1}H NMR spectrum of compound **2b**

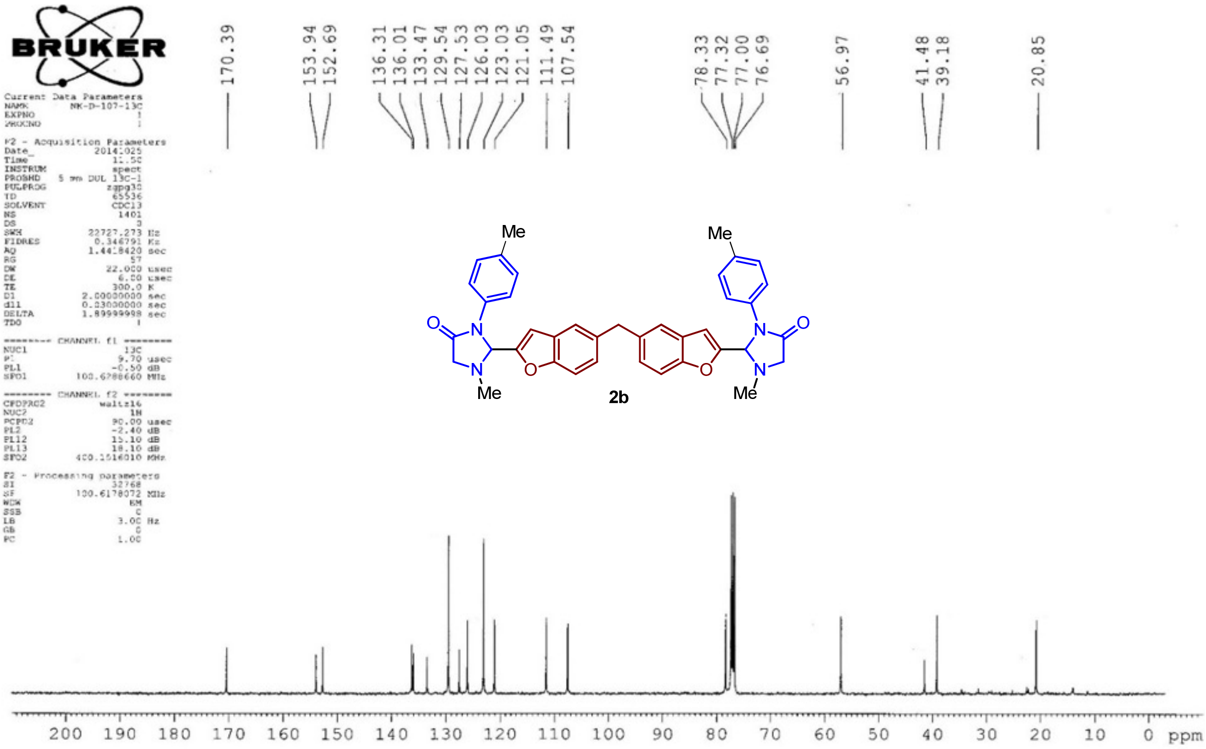

^{13}C NMR spectrum of compound **2b**

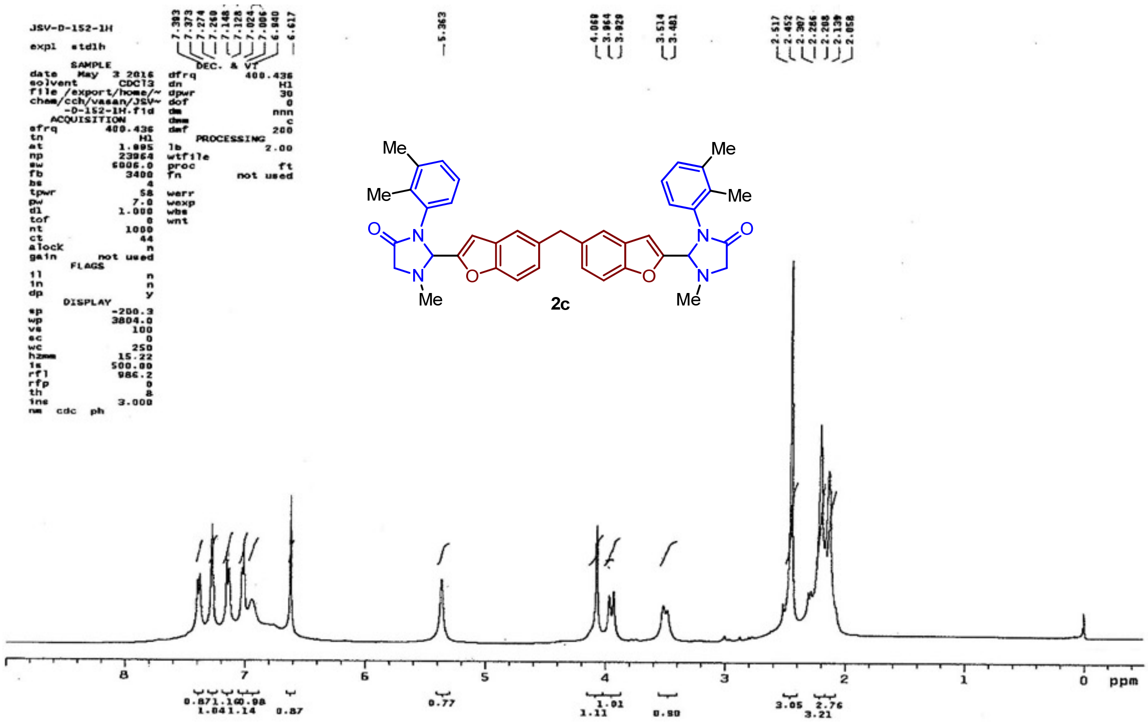

^{1}H NMR spectrum of compound **2c**

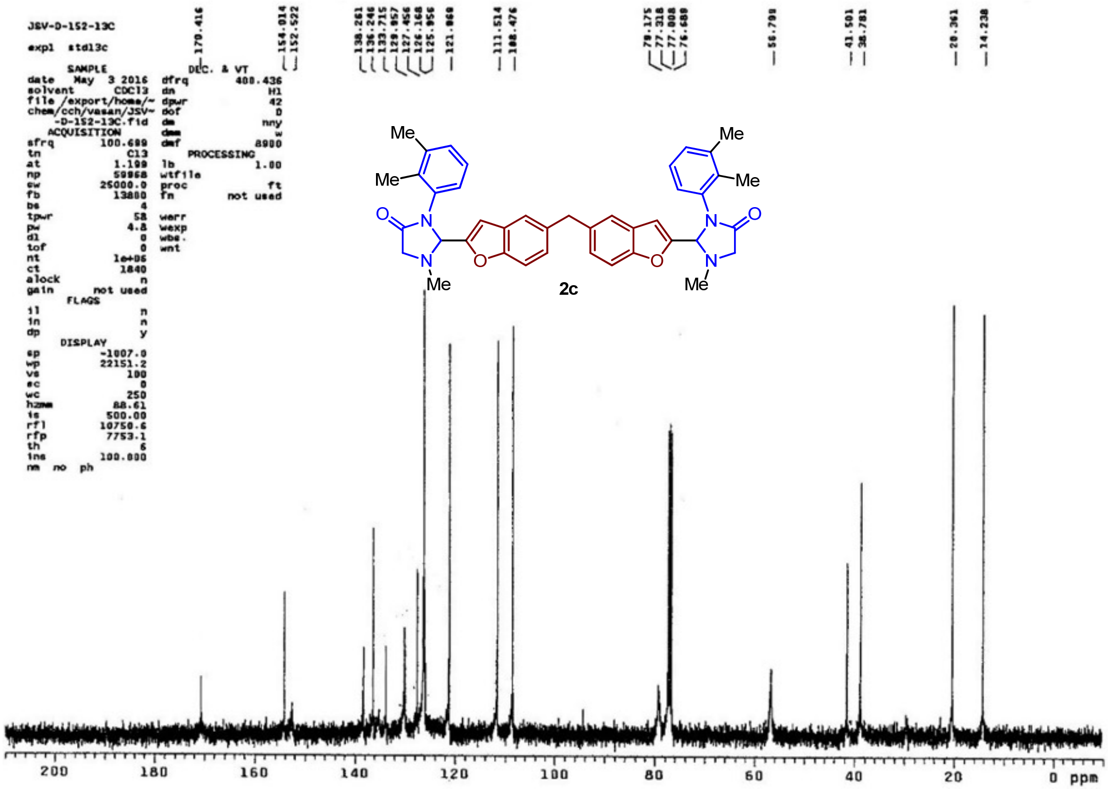

^{13}C NMR spectrum of compound **2c**

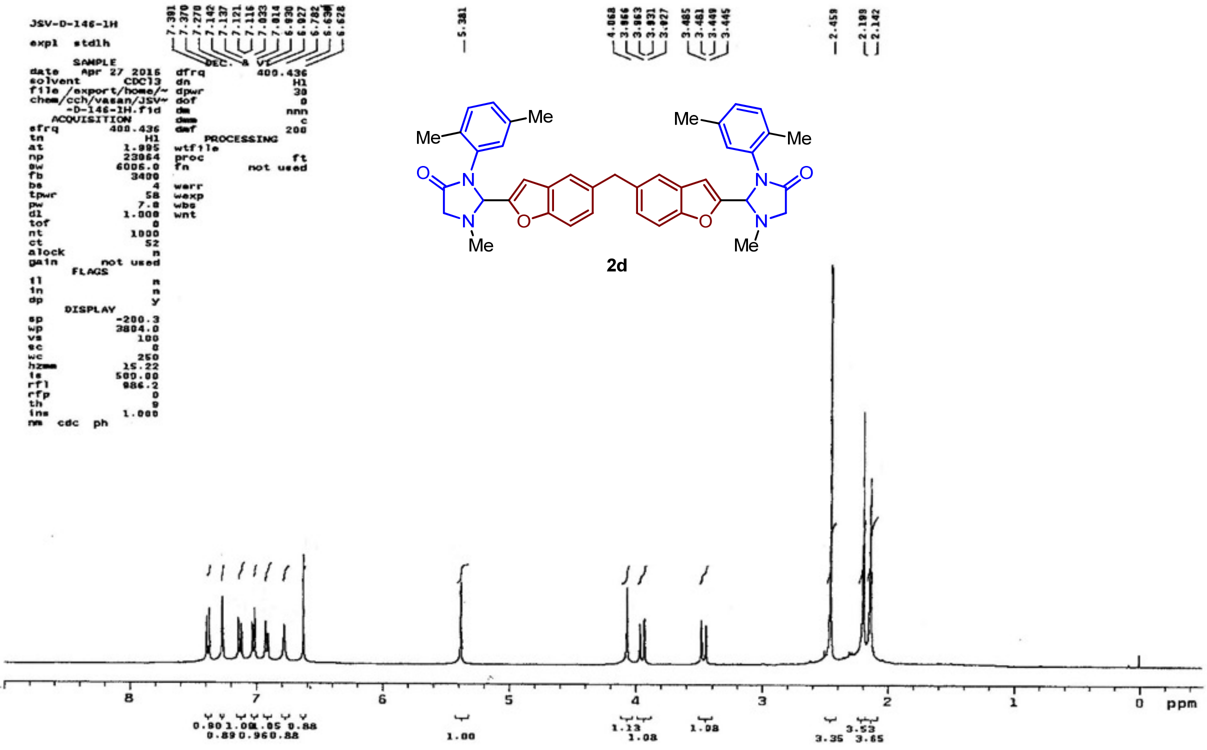

^{1}H NMR spectrum of compound **2d**

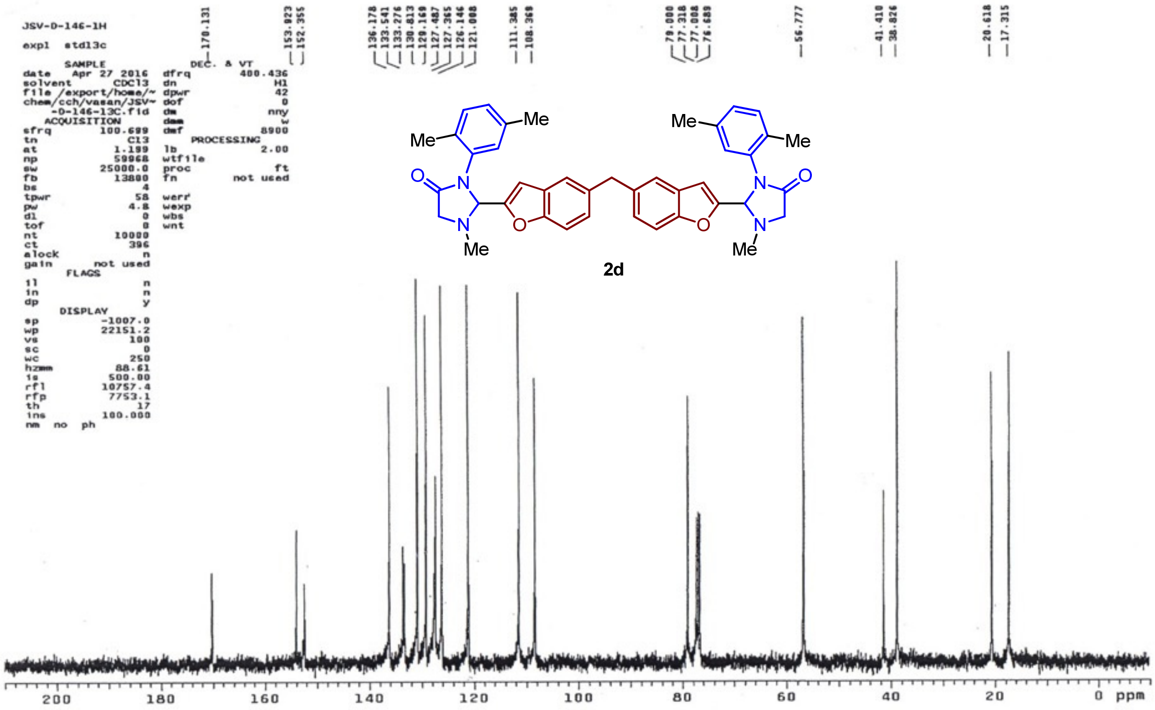

^{13}C NMR spectrum of compound **2d**

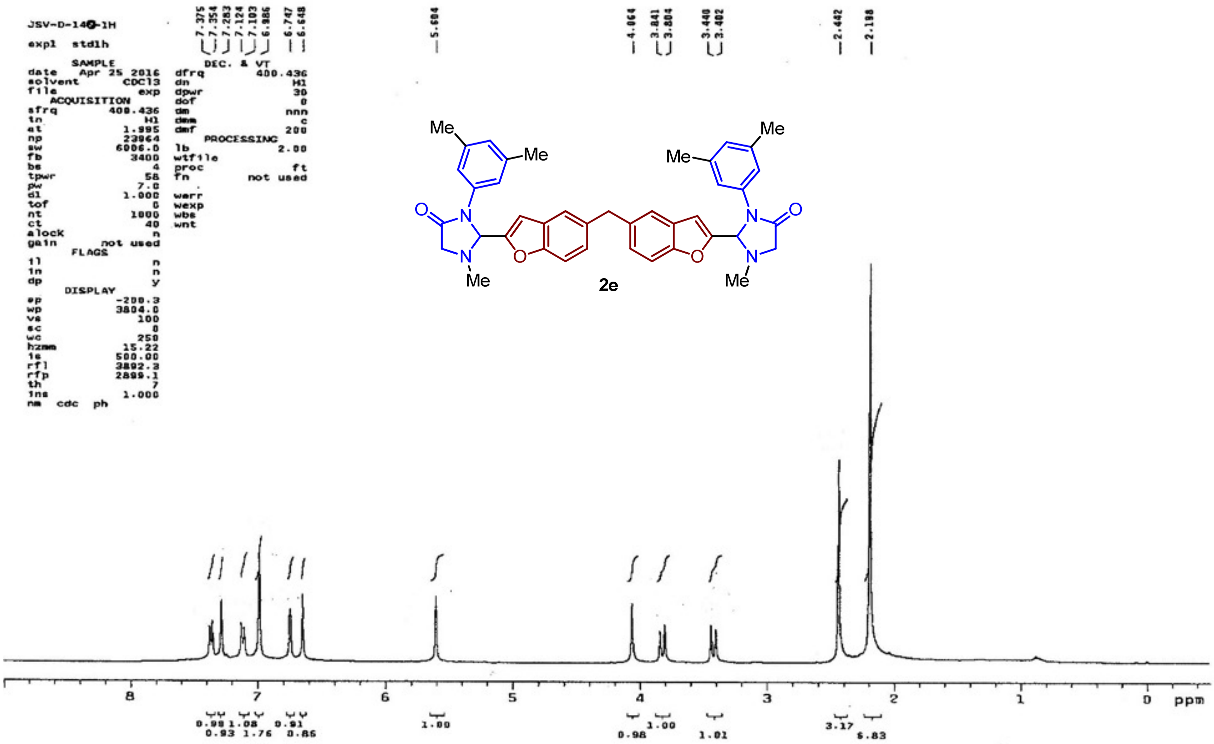

^{1}H NMR spectrum of compound **2e**

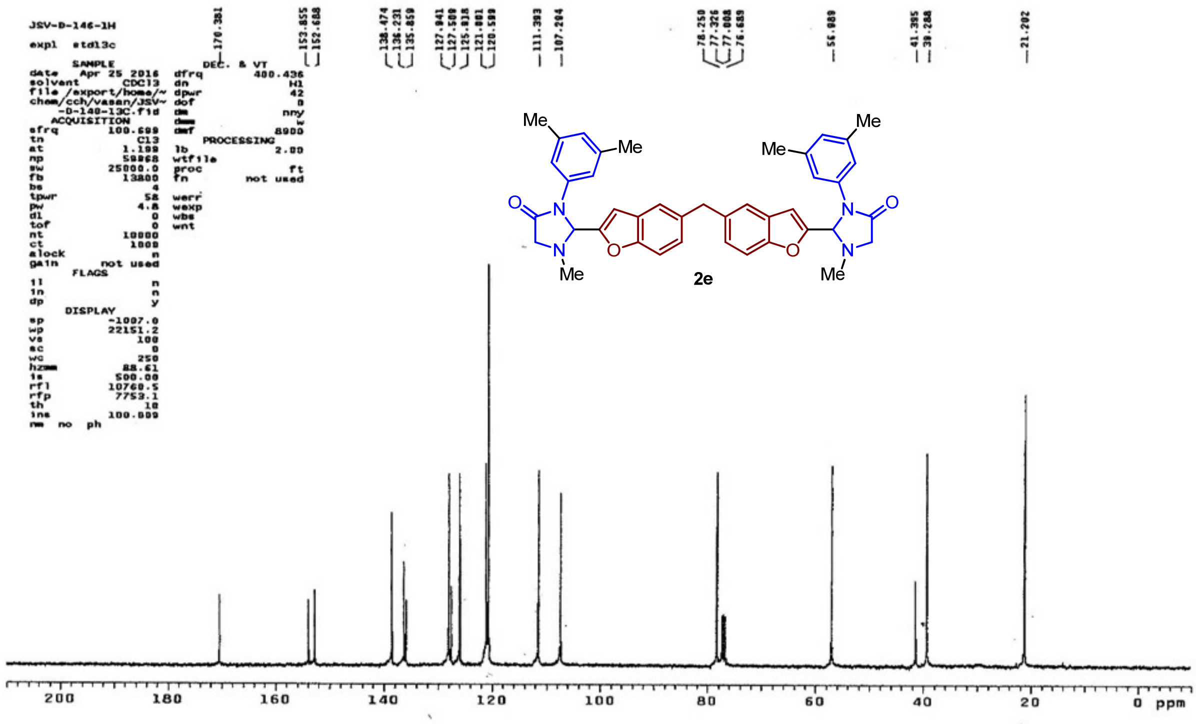

^{13}C NMR spectrum of compound **2e**

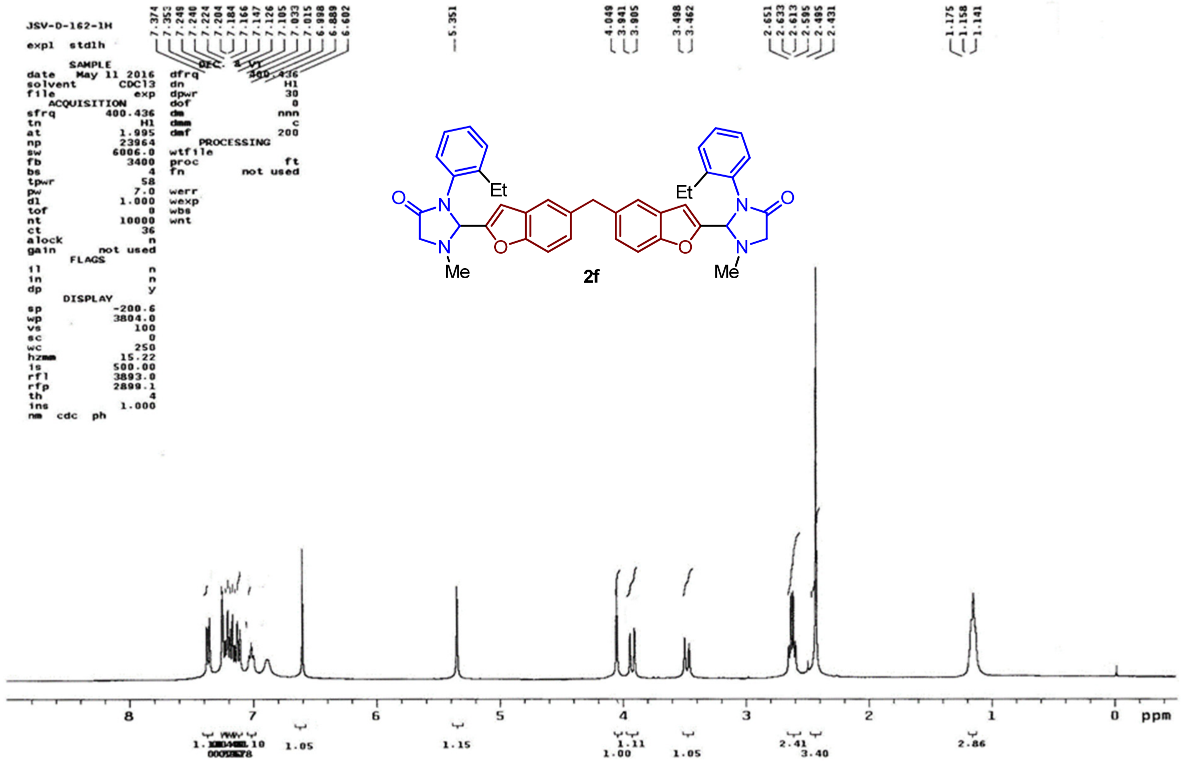

^{1}H NMR spectrum of compound **2f**

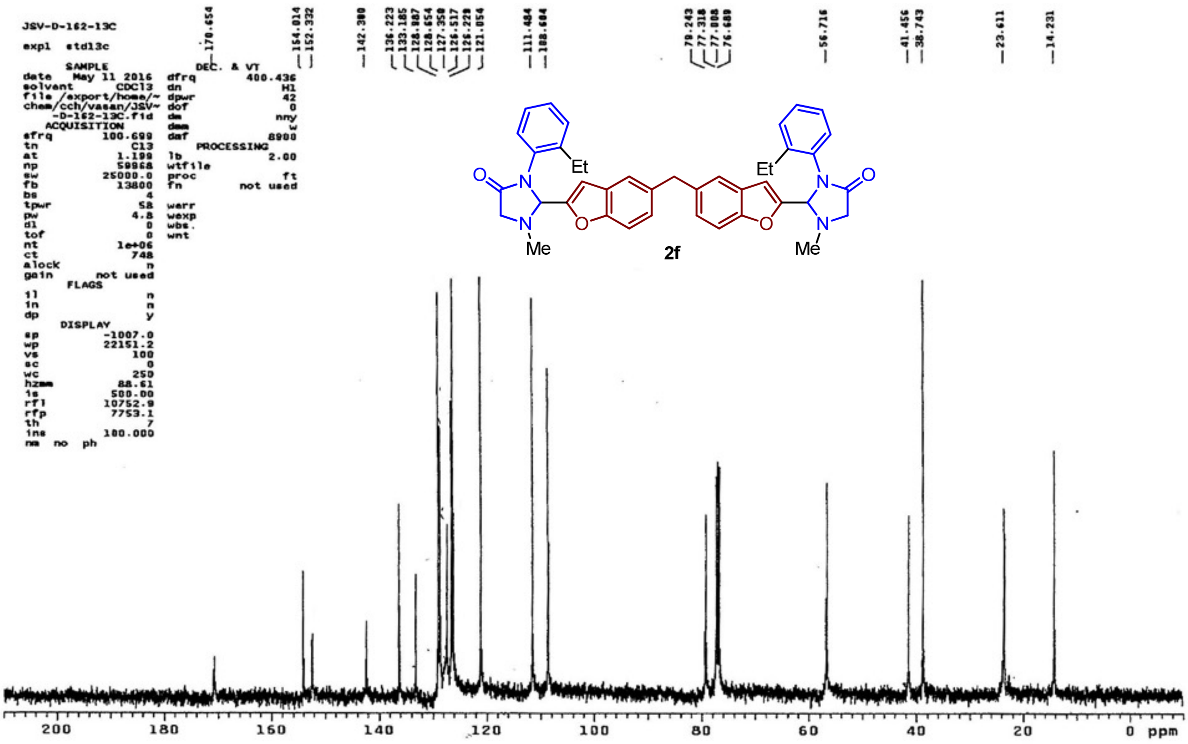

^{13}C NMR spectrum of compound **2f**

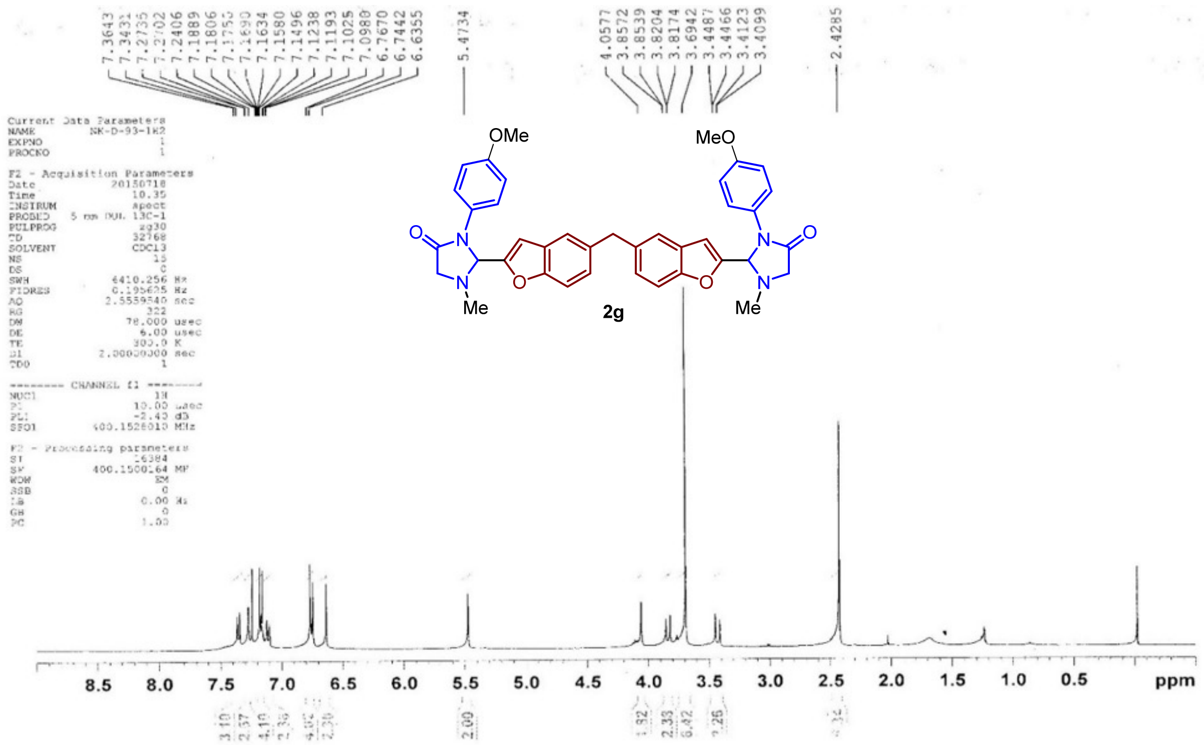

^{1}H NMR spectrum of compound **2g**

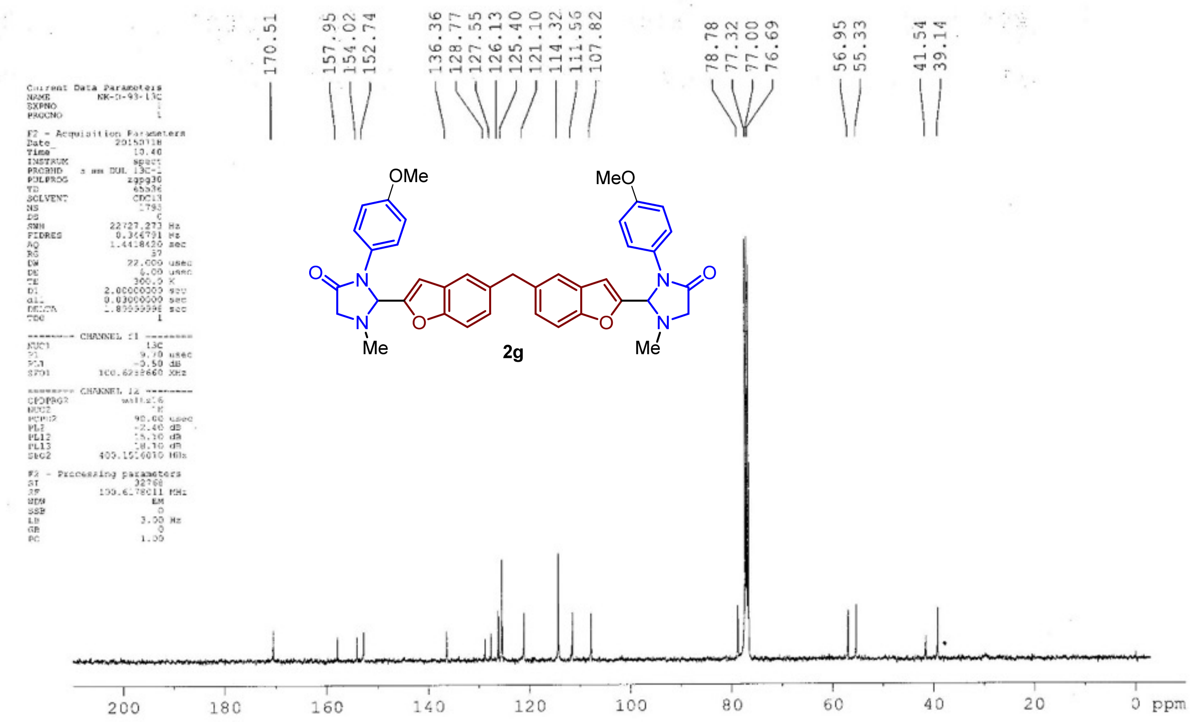

^{13}C NMR spectrum of compound **2g**

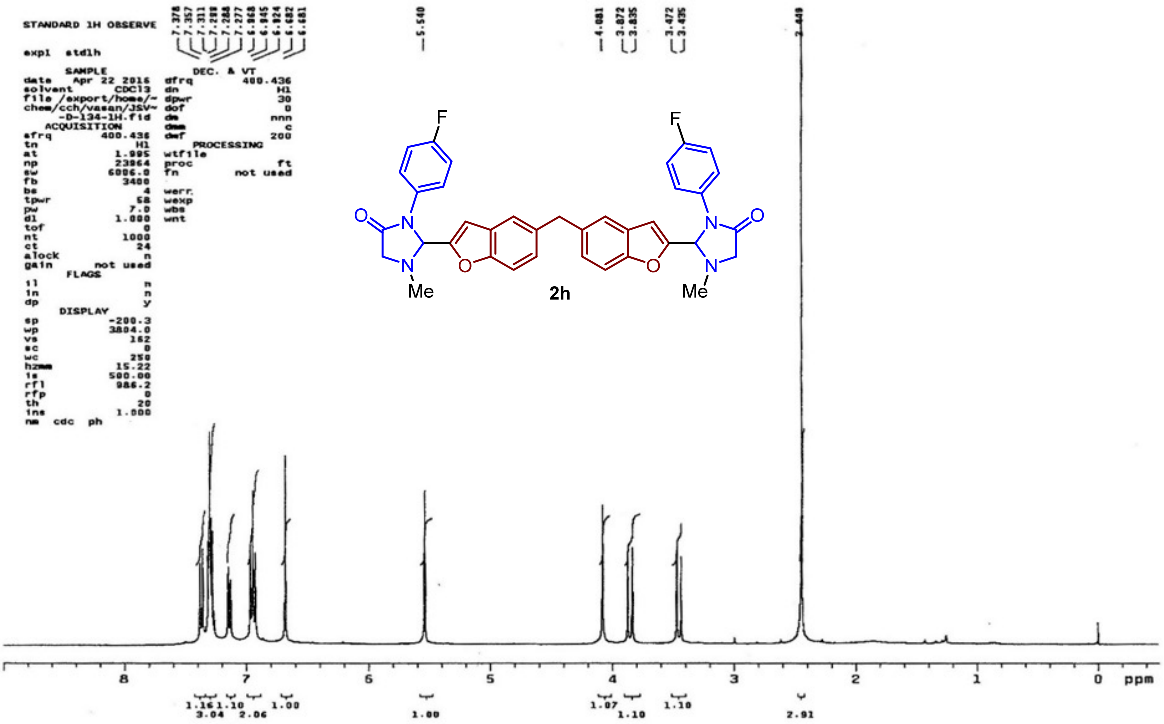

^{1}H NMR spectrum of compound **2h**

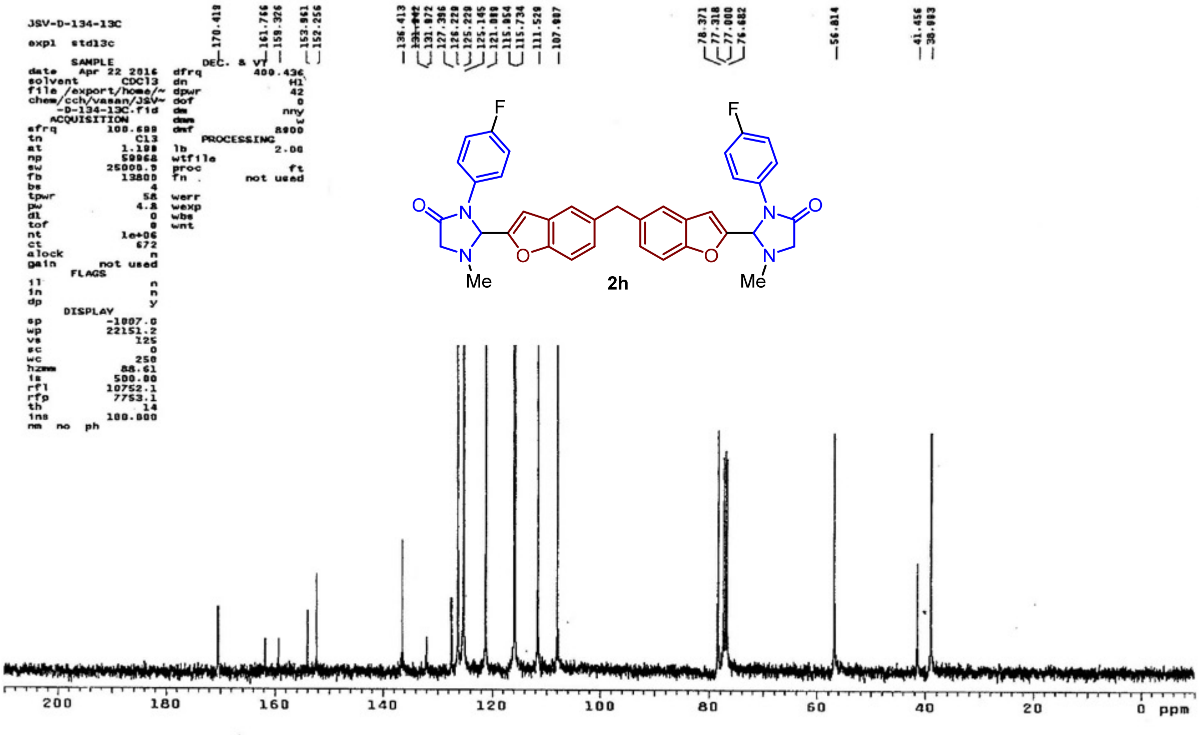

^{13}C NMR spectrum of compound **2h**

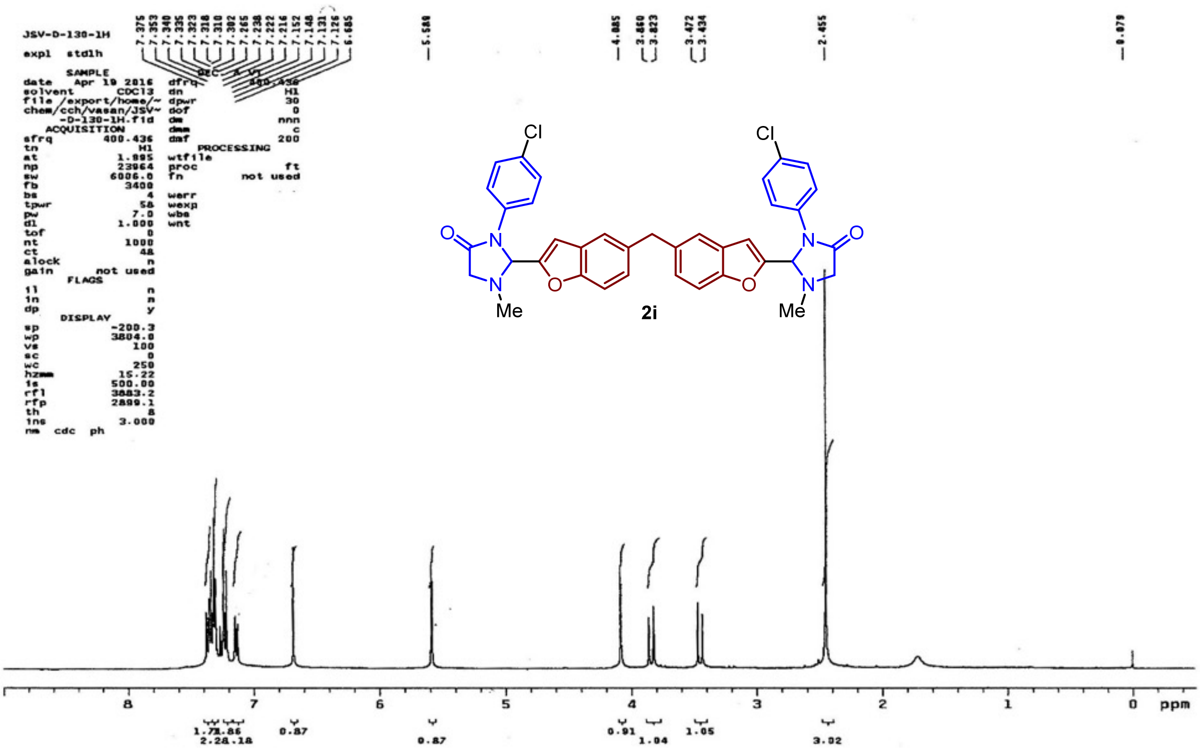

^{1}H NMR spectrum of compound **2i**

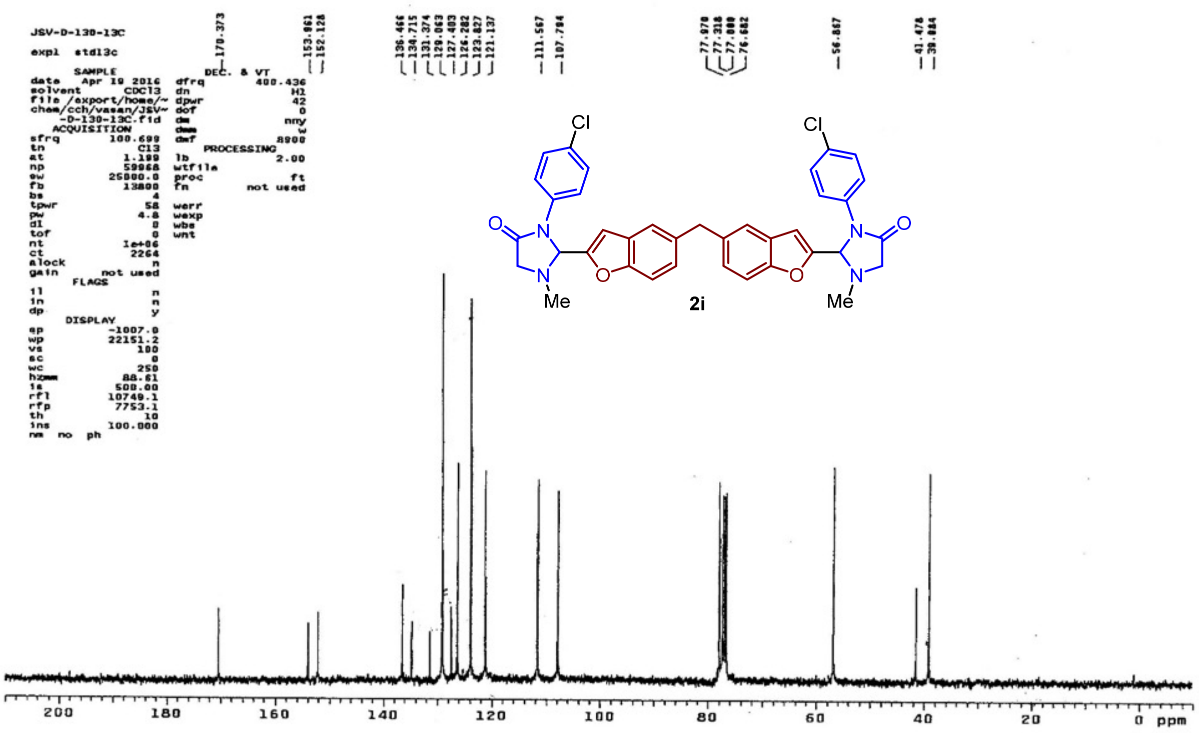

^{13}C NMR spectrum of compound **2i**

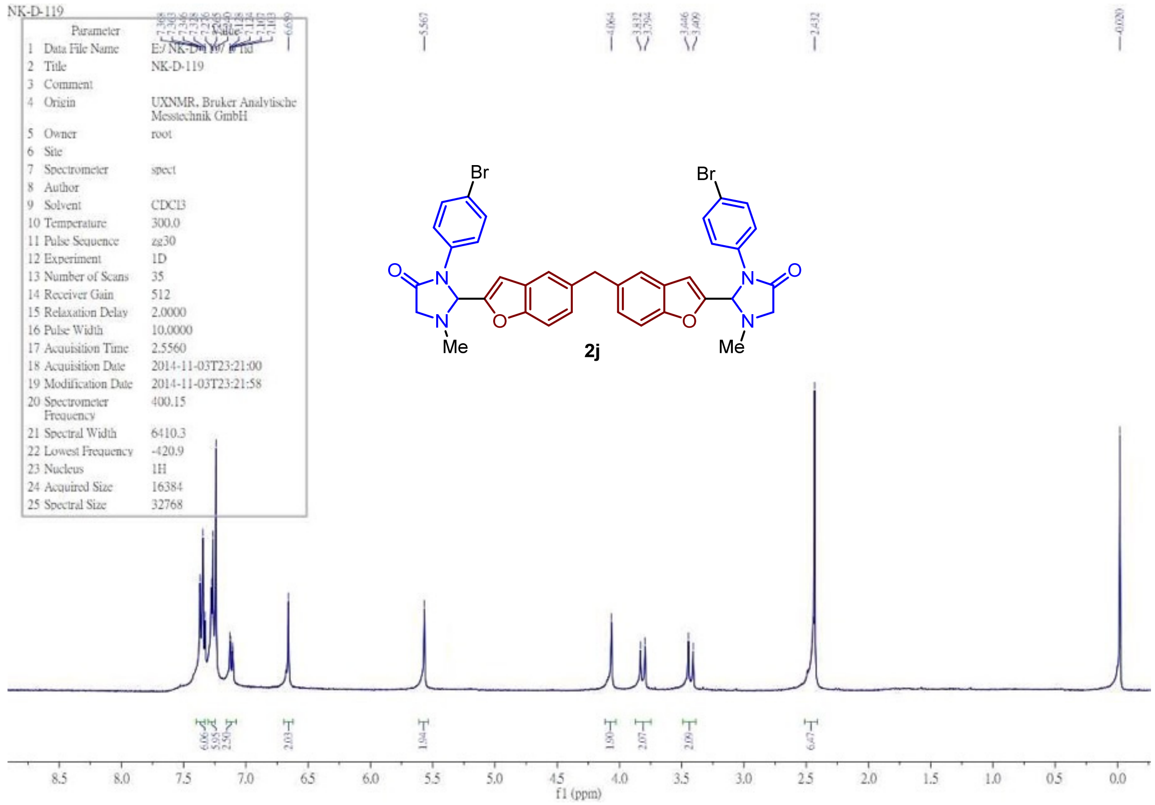

^{1}H NMR spectrum of compound **2j**

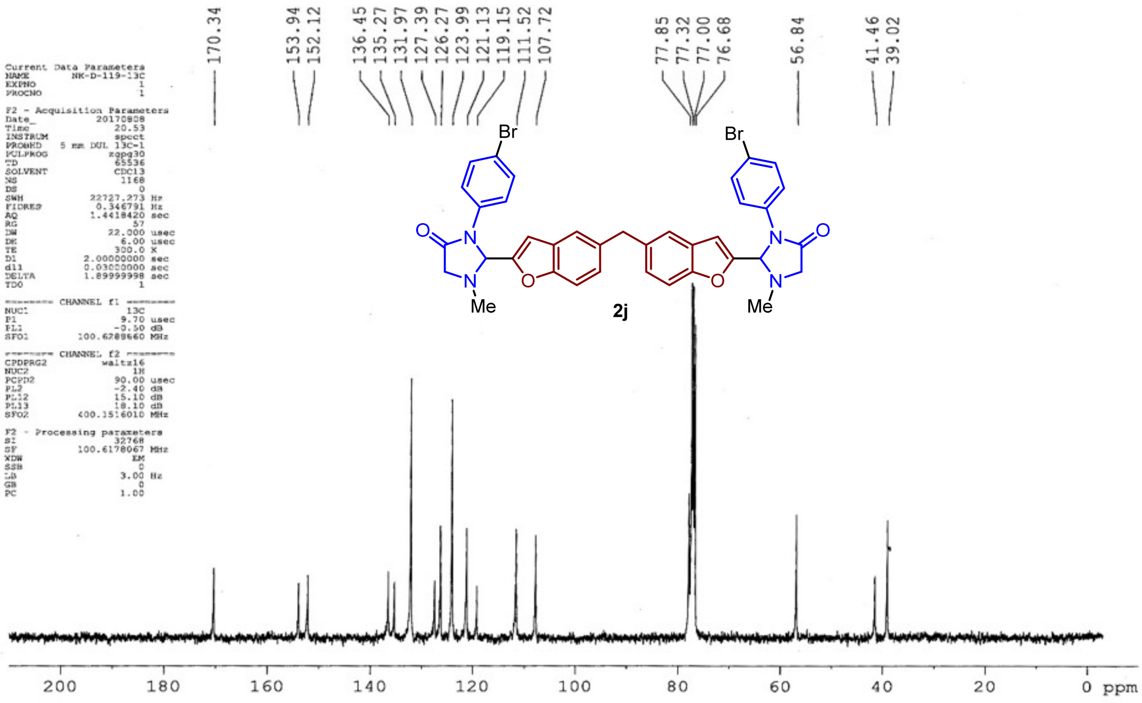

^{13}C NMR spectrum of compound **2j**

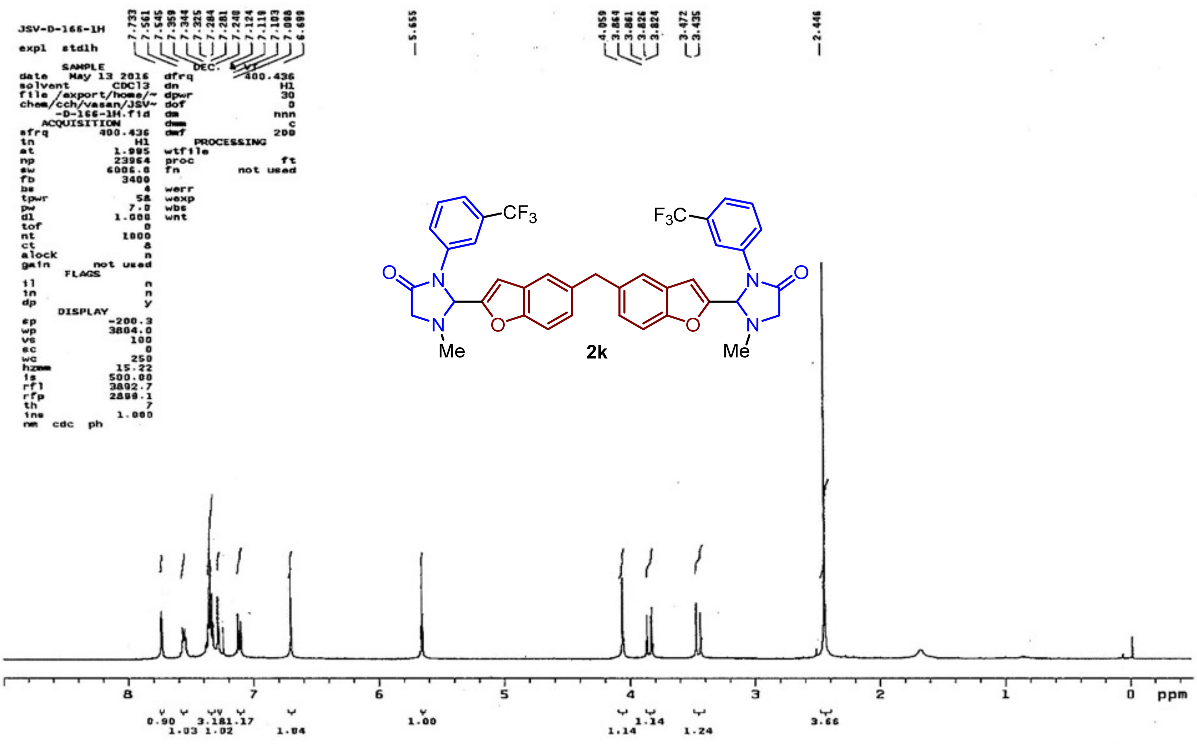

^{1}H NMR spectrum of compound **2k**

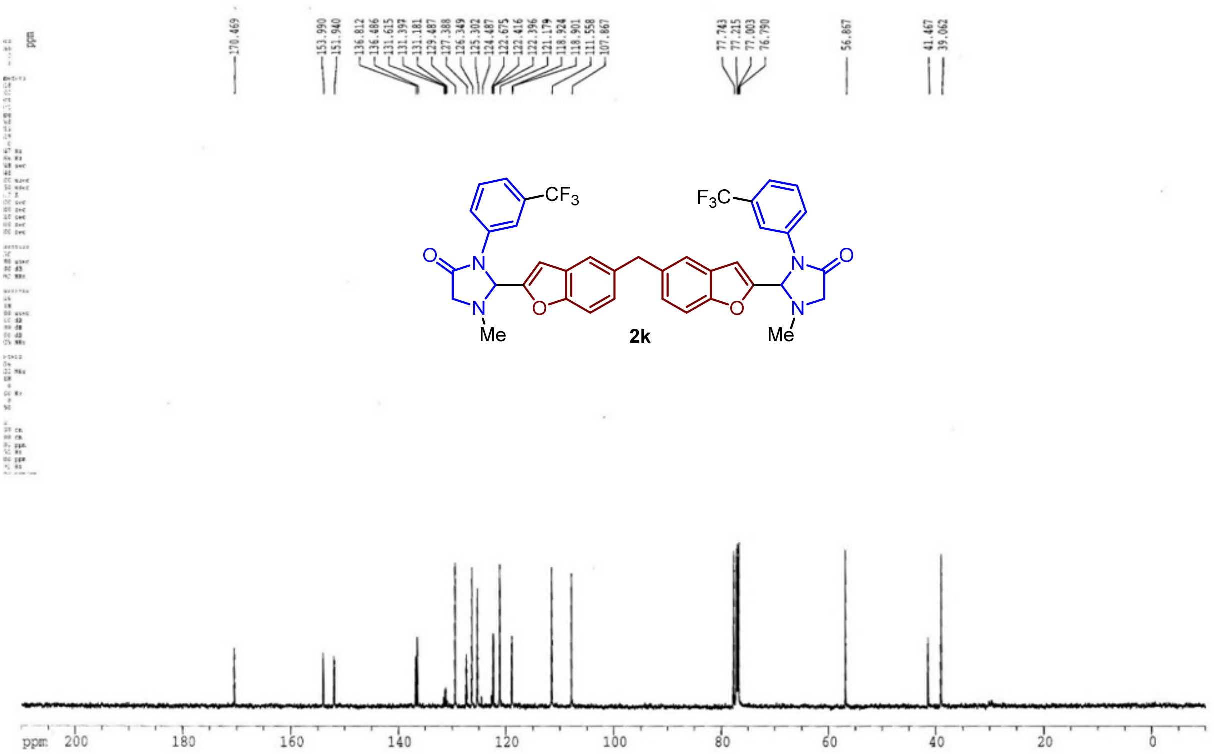

^{13}C NMR spectrum of compound **2k**

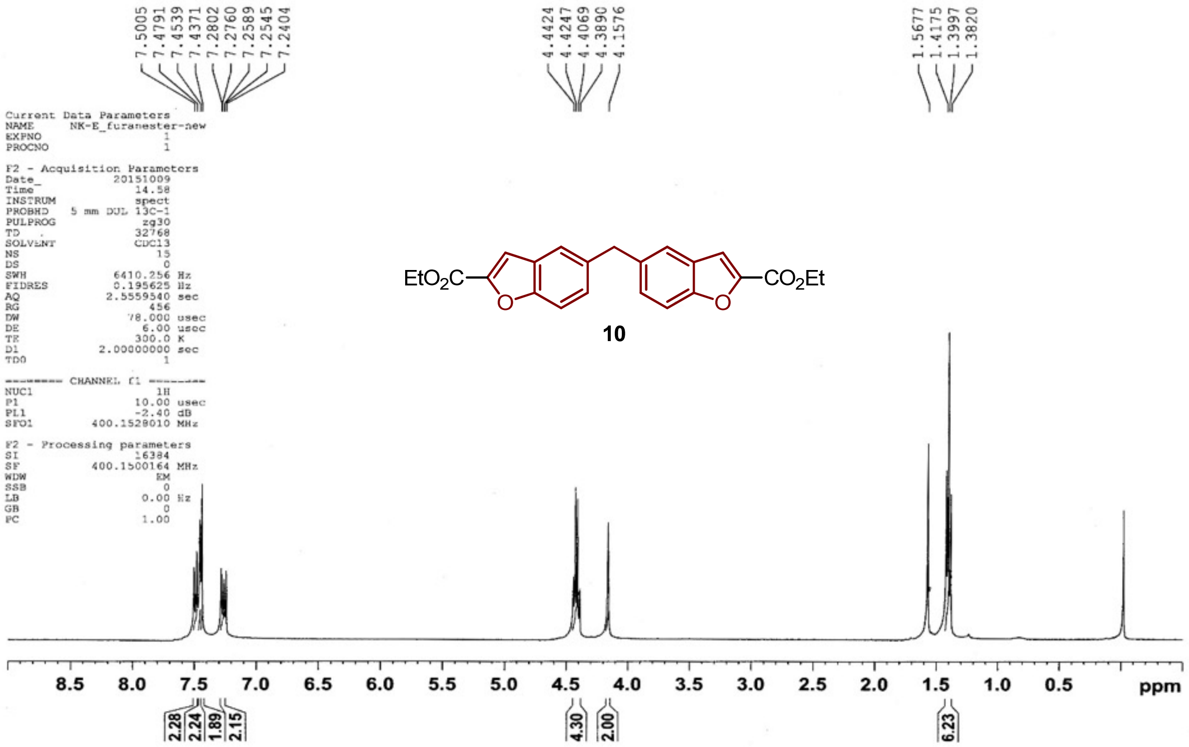

^{1}H NMR spectrum of compound **10**

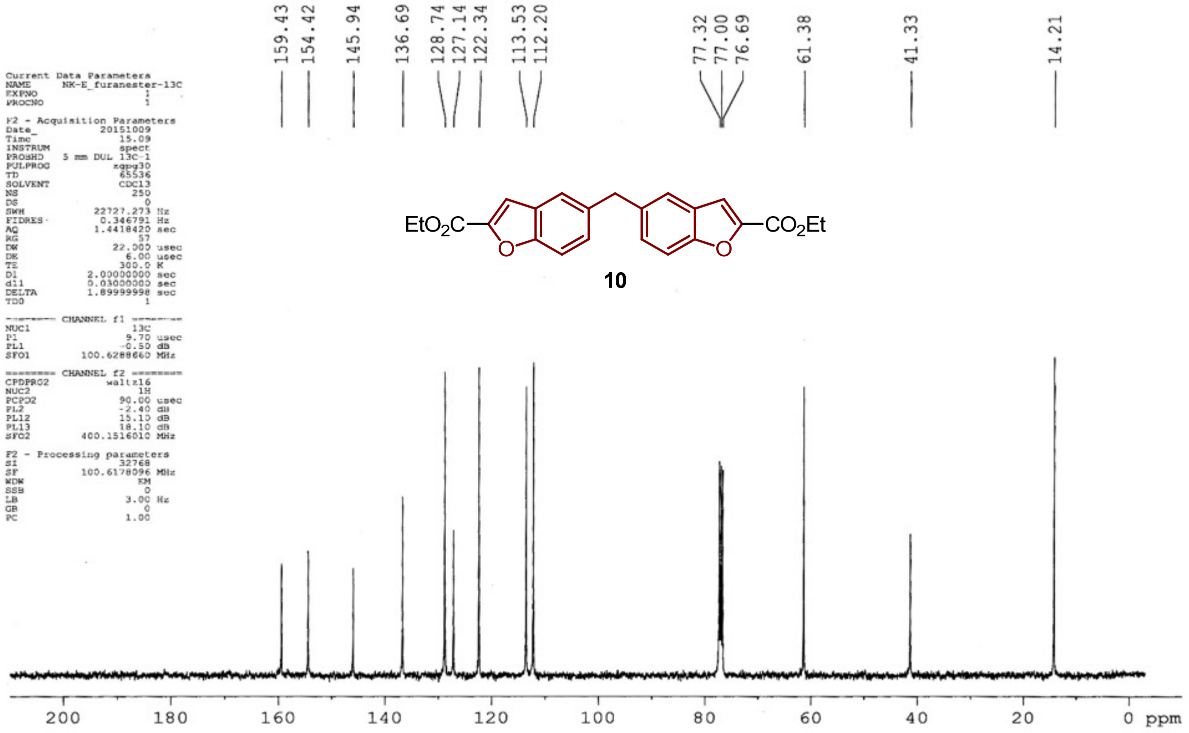

^{13}C NMR spectrum of compound **10**

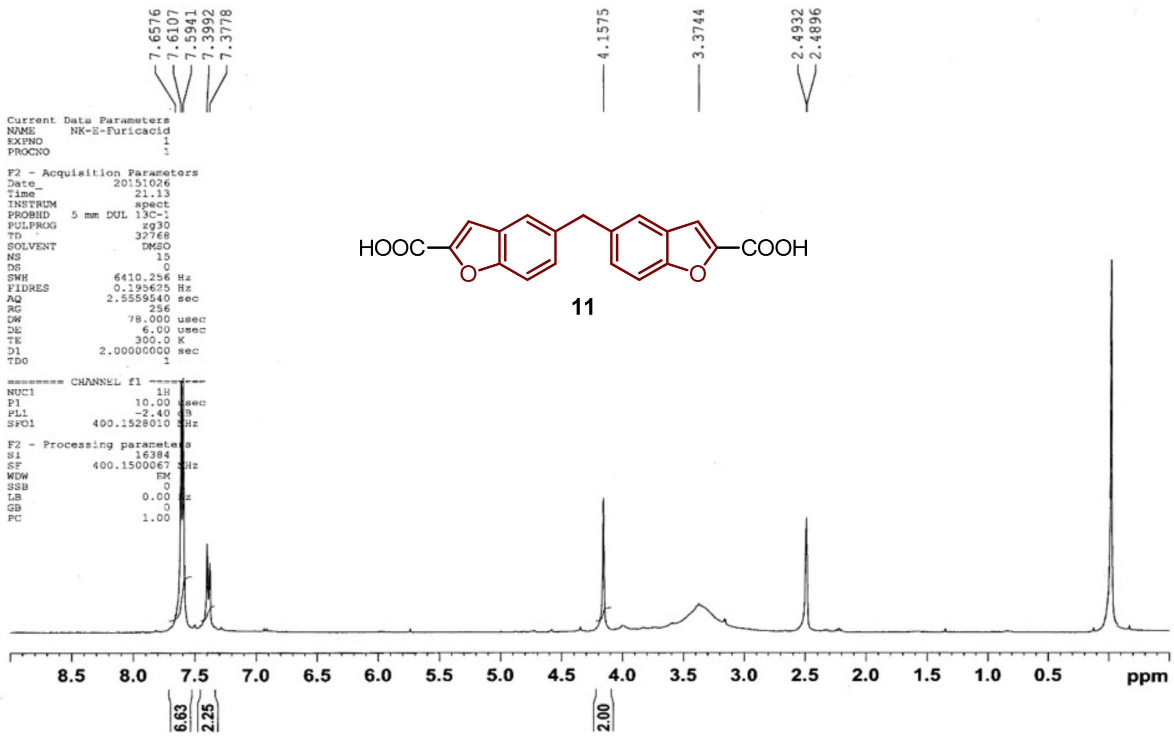

^{1}H NMR spectrum of compound **11**

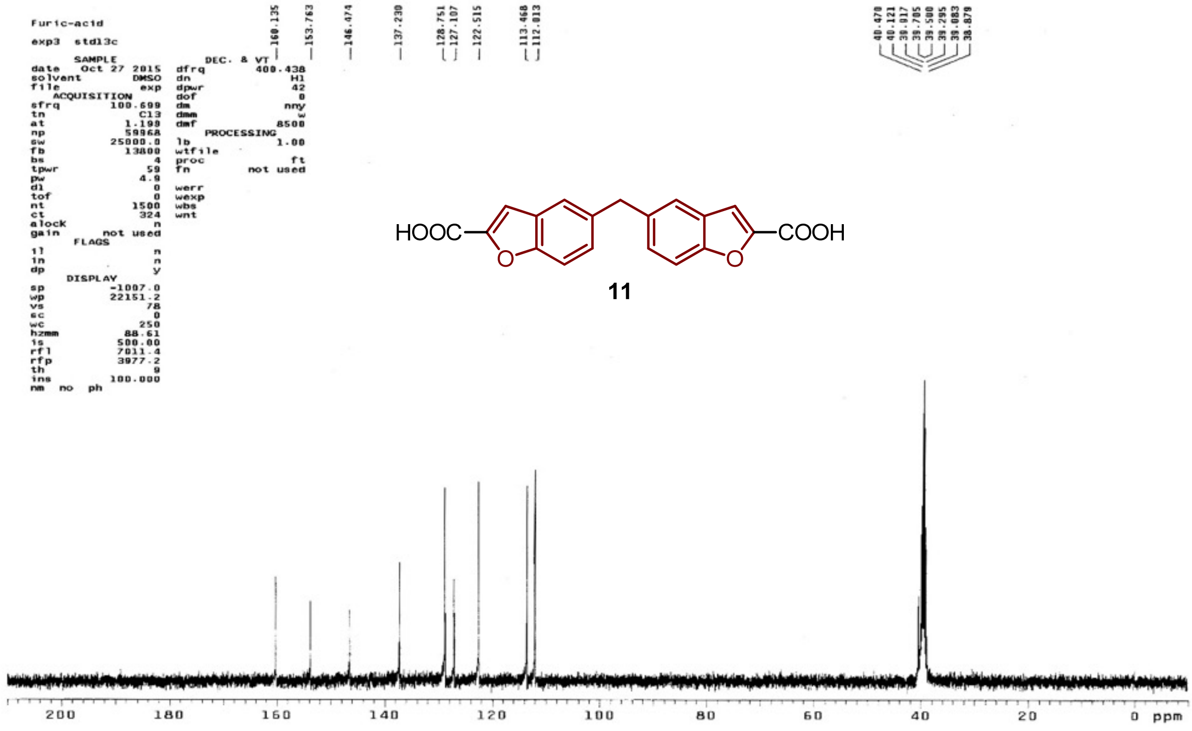

^{13}C NMR spectrum of compound **11**

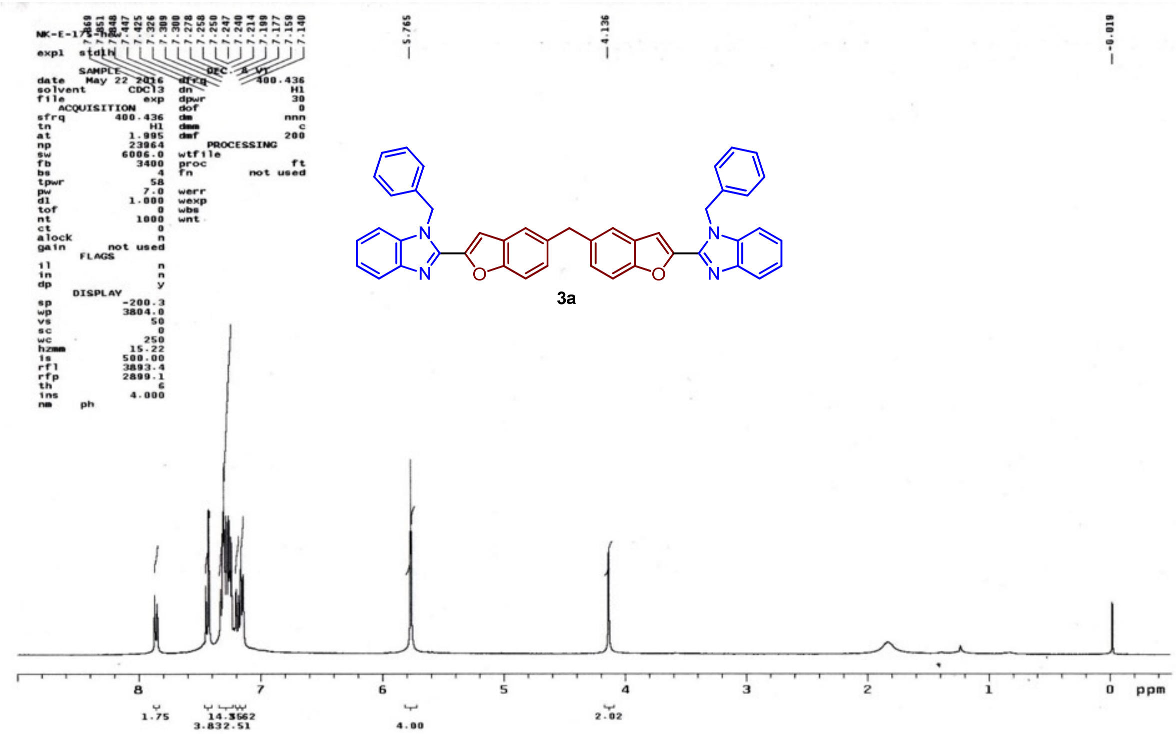

^{1}H NMR spectrum of compound **3a**

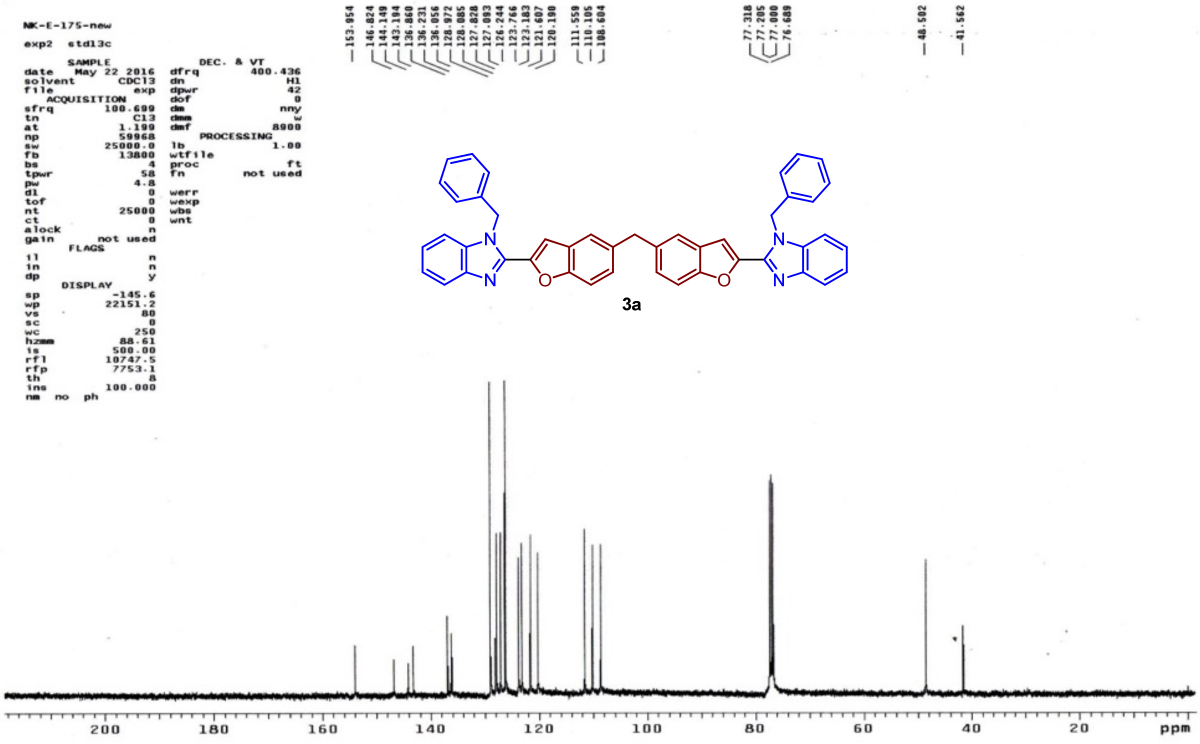

^{13}C NMR spectrum of compound **3a**

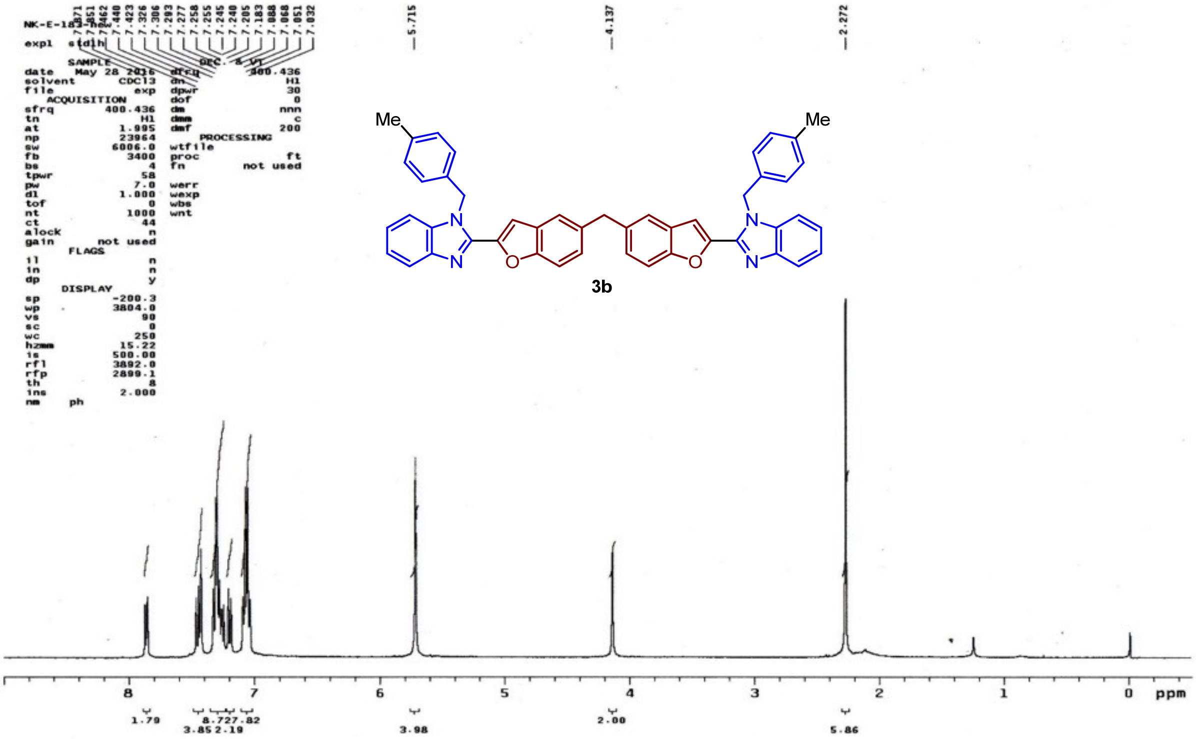

^{1}H NMR spectrum of compound **3b**

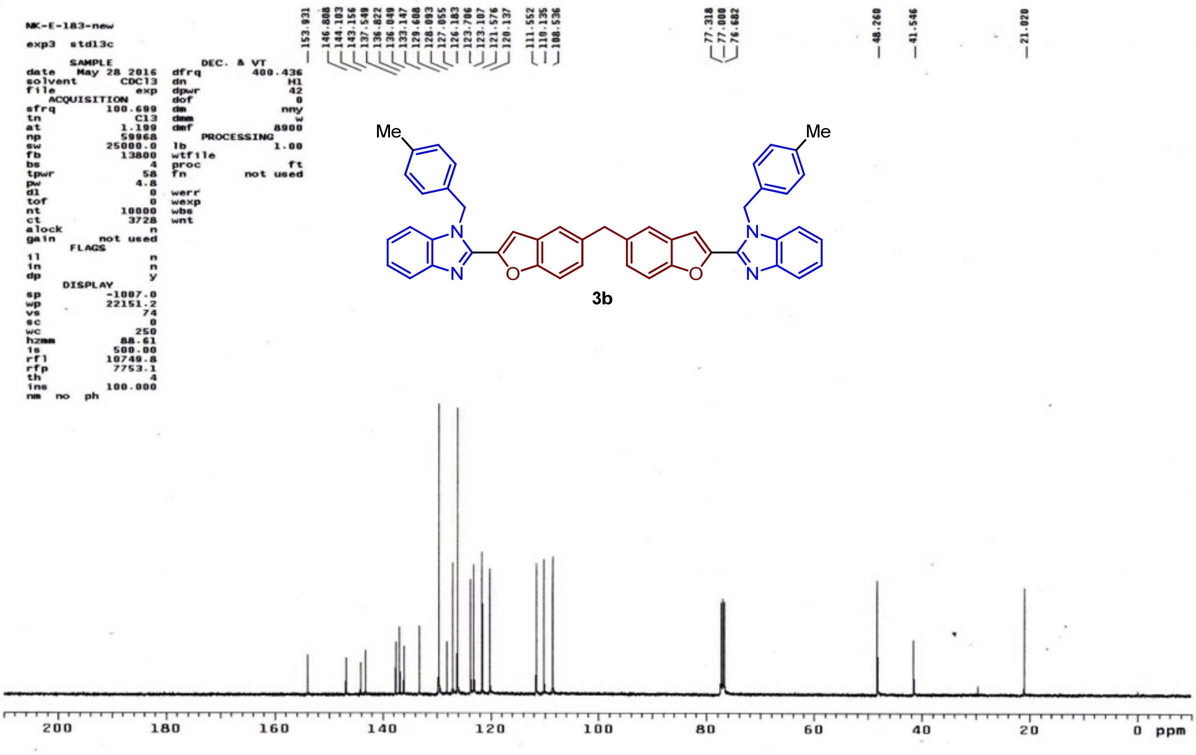

^{13}C NMR spectrum of compound **3b**

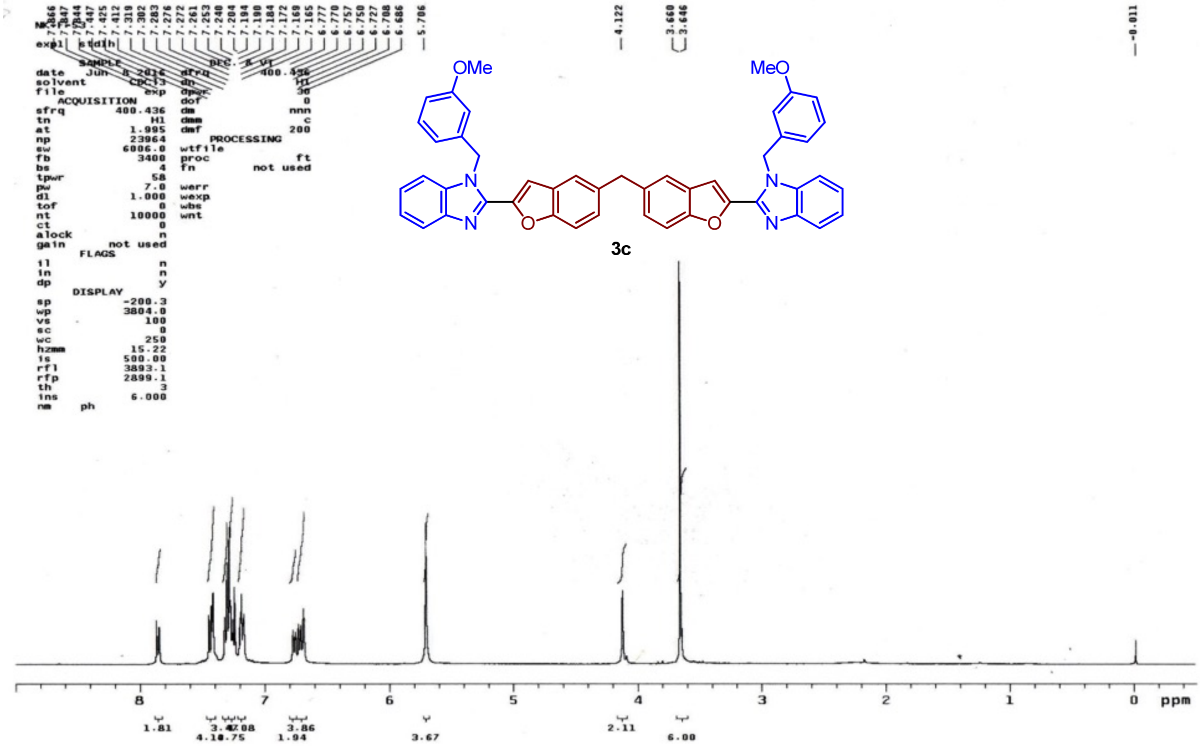

^{1}H NMR spectrum of compound **3c**

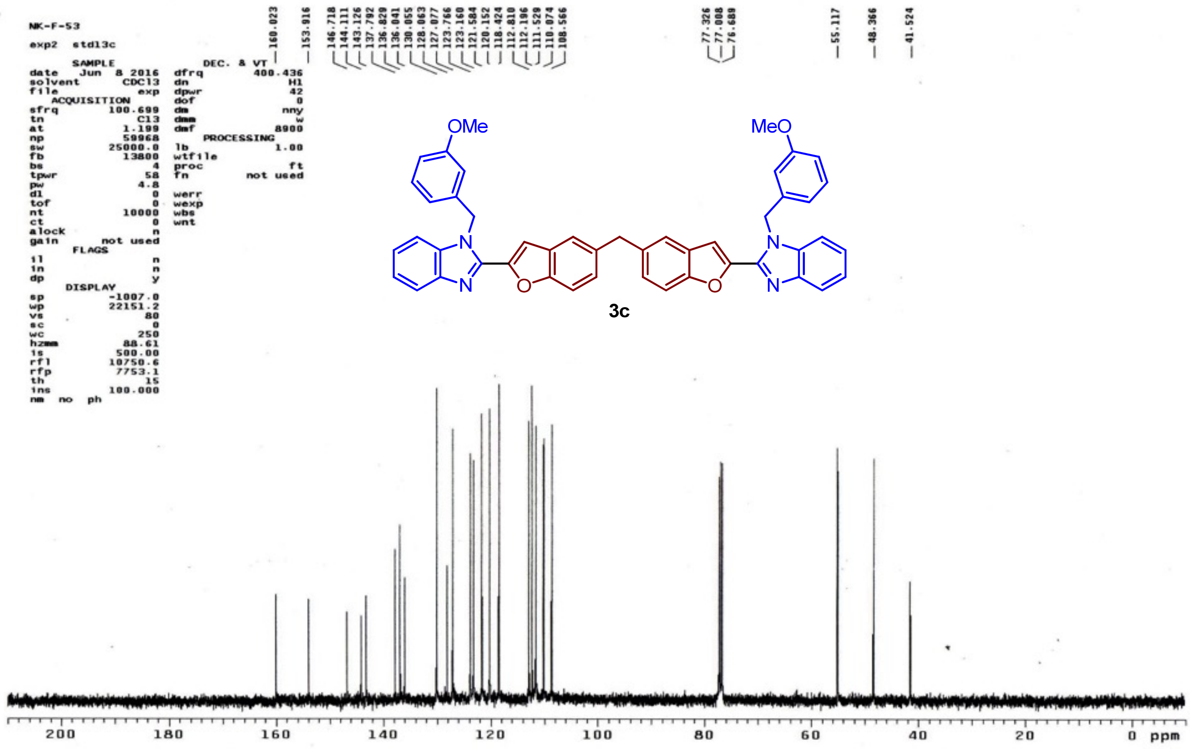

^{13}C NMR spectrum of compound **3c**

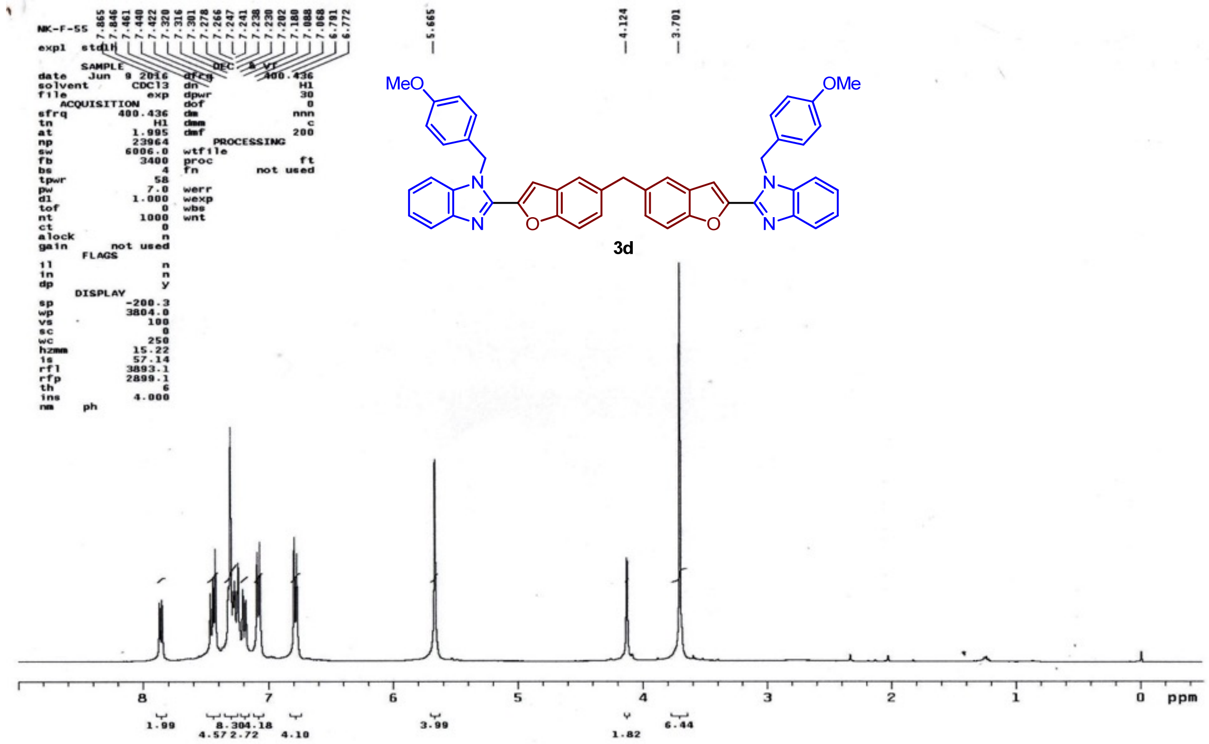

^{1}H NMR spectrum of compound **3d**

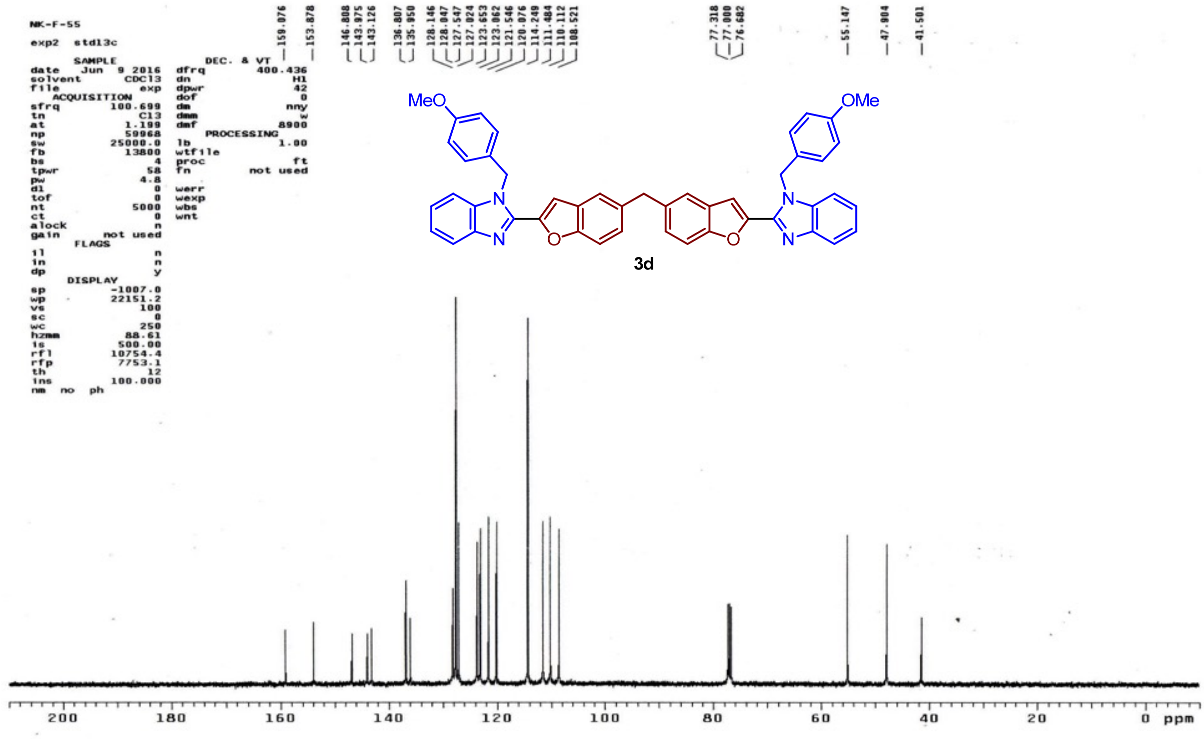

^{13}C NMR spectrum of compound **3d**

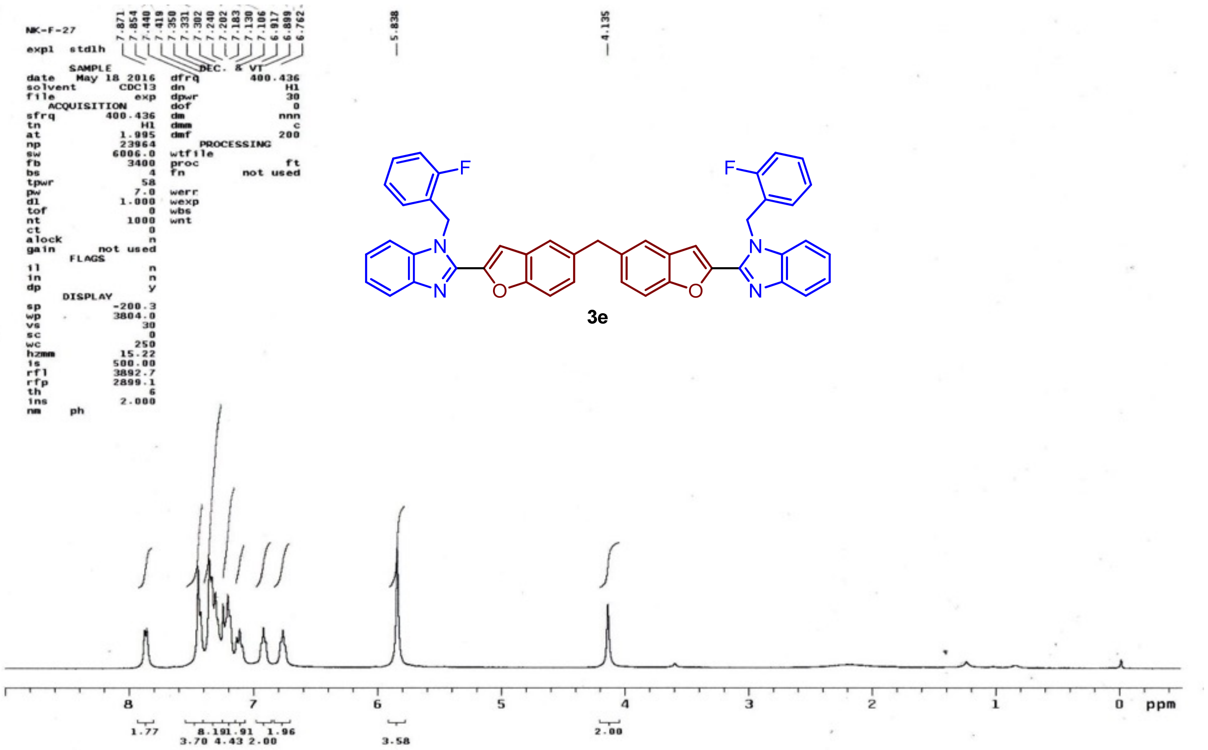

^{1}H NMR spectrum of compound **3e**

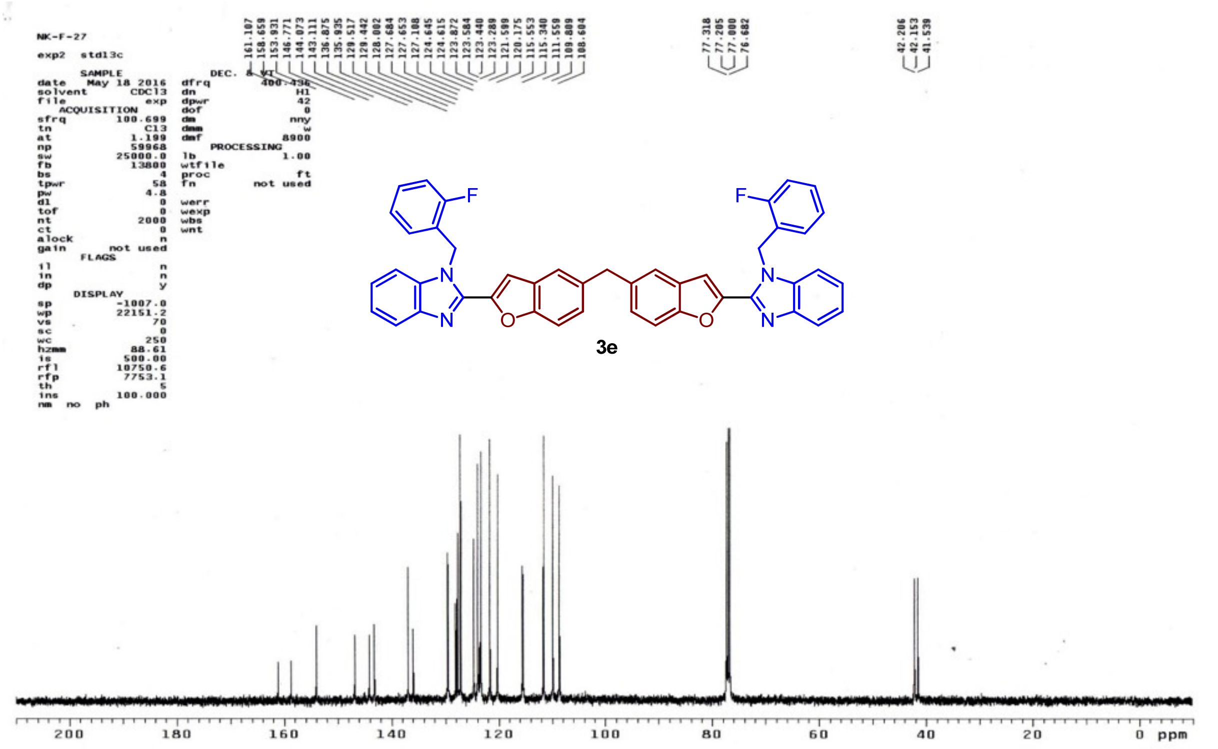

^{13}C NMR spectrum of compound **3e**

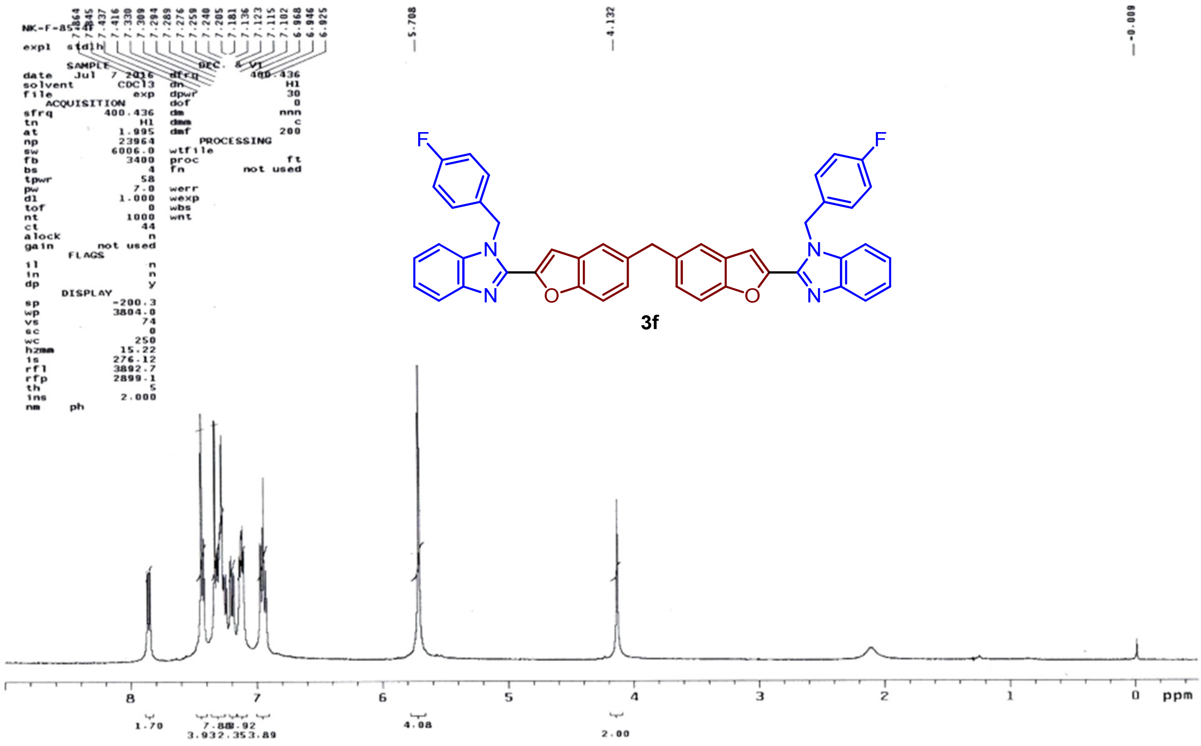

^{1}H NMR spectrum of compound **3f**

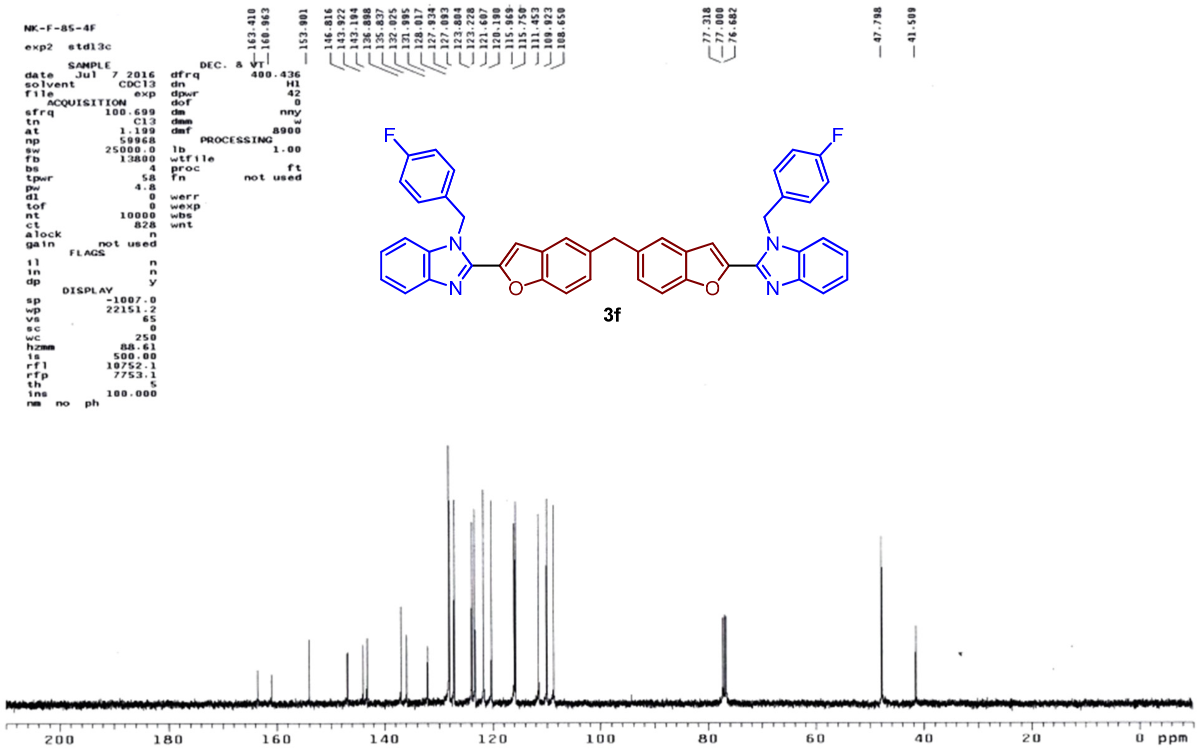

^{13}C NMR spectrum of compound **3f**

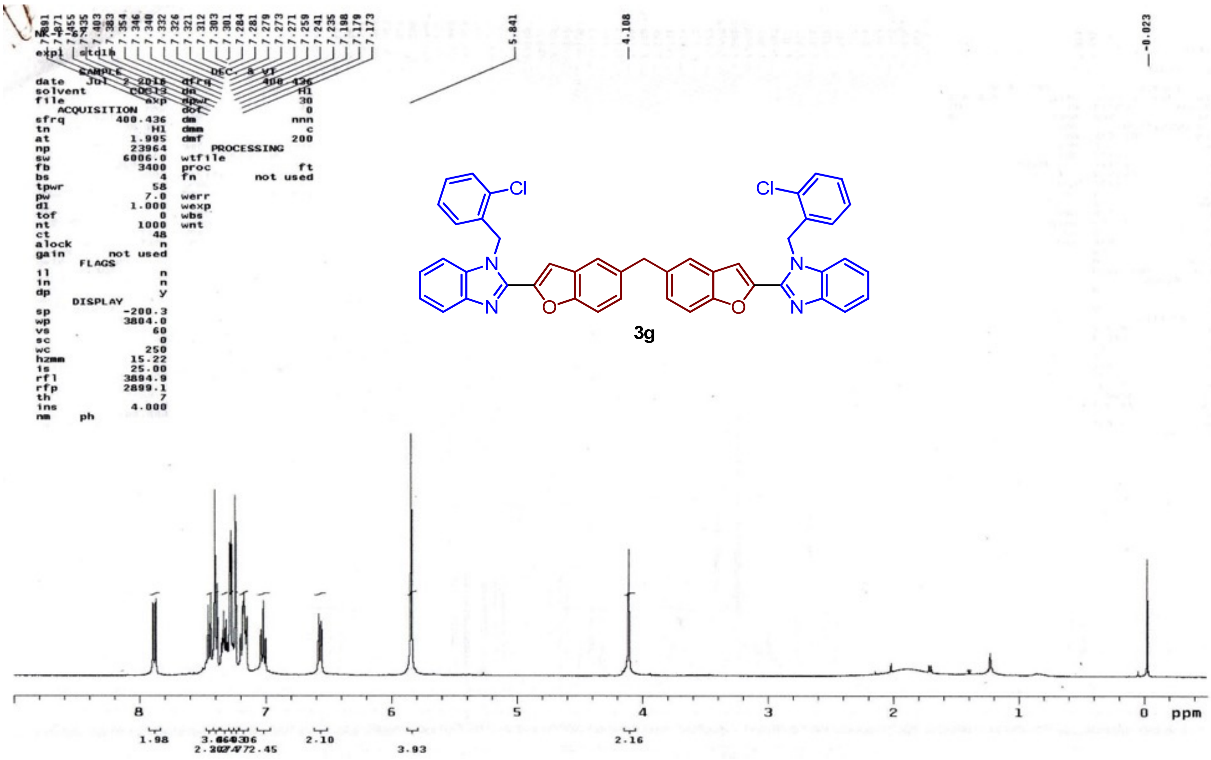

^{1}H NMR spectrum of compound **3g**

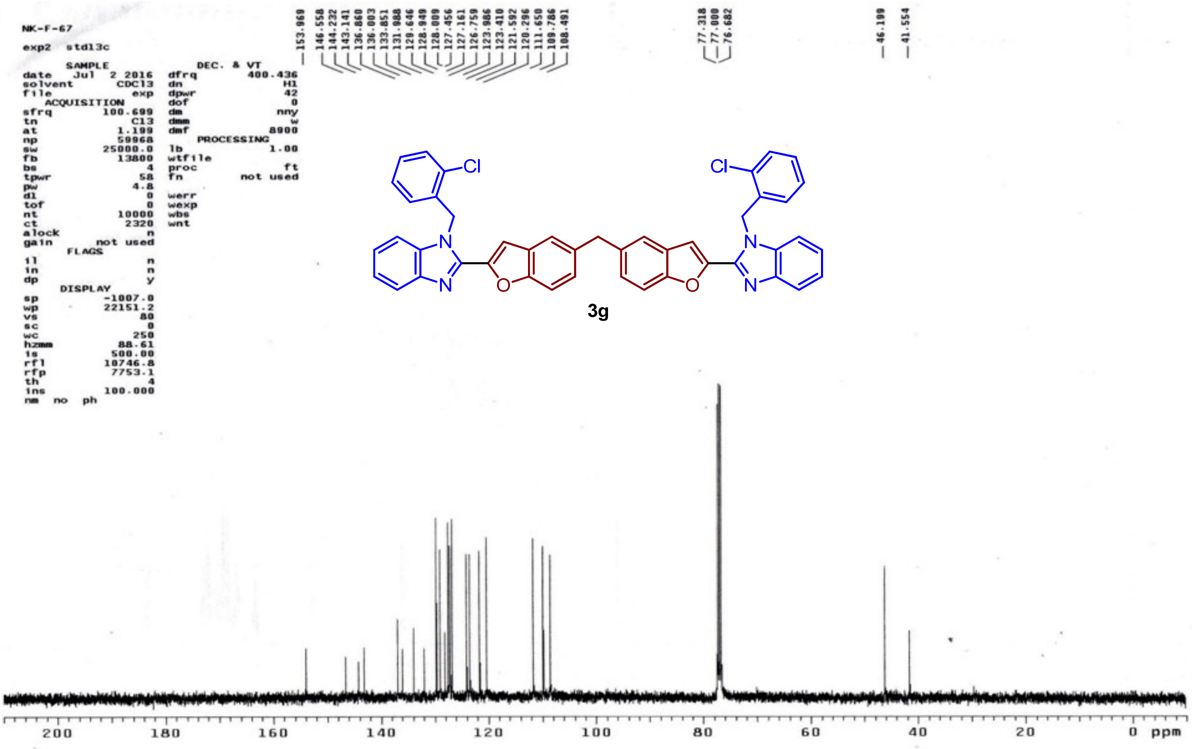

^{13}C NMR spectrum of compound **3g**

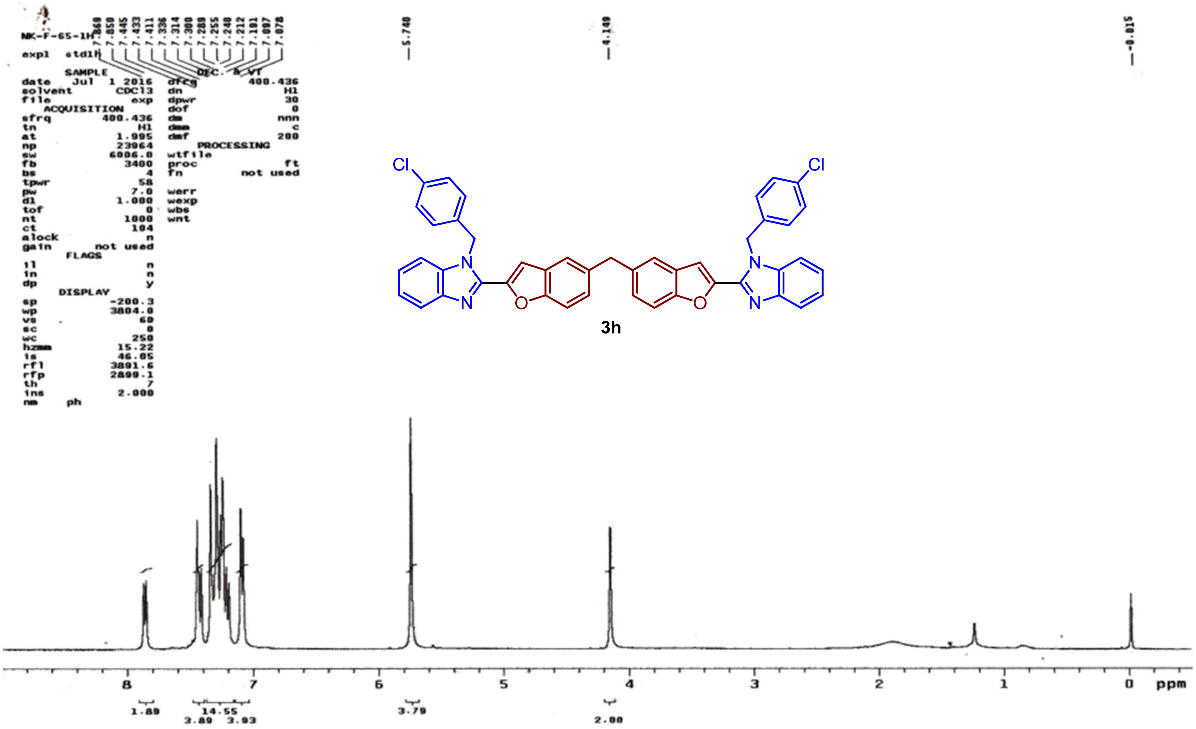

^{1}H NMR spectrum of compound **3h**

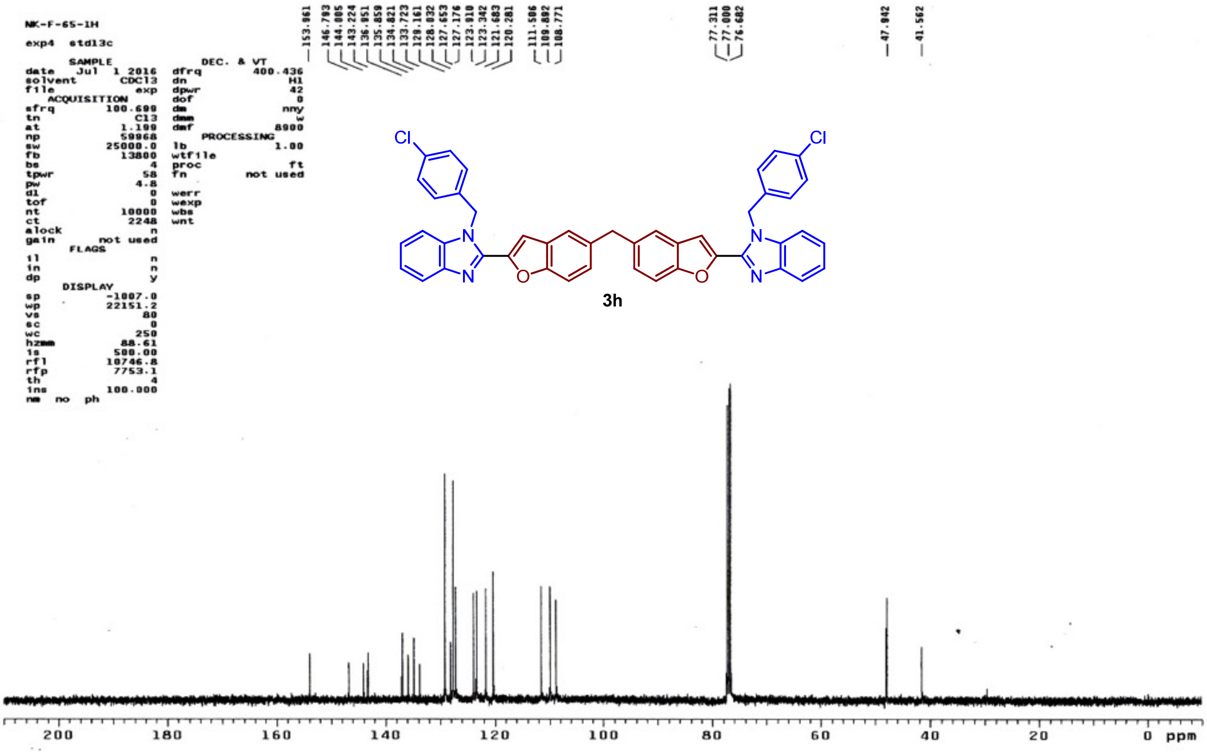

^{13}C NMR spectrum of compound **3h**

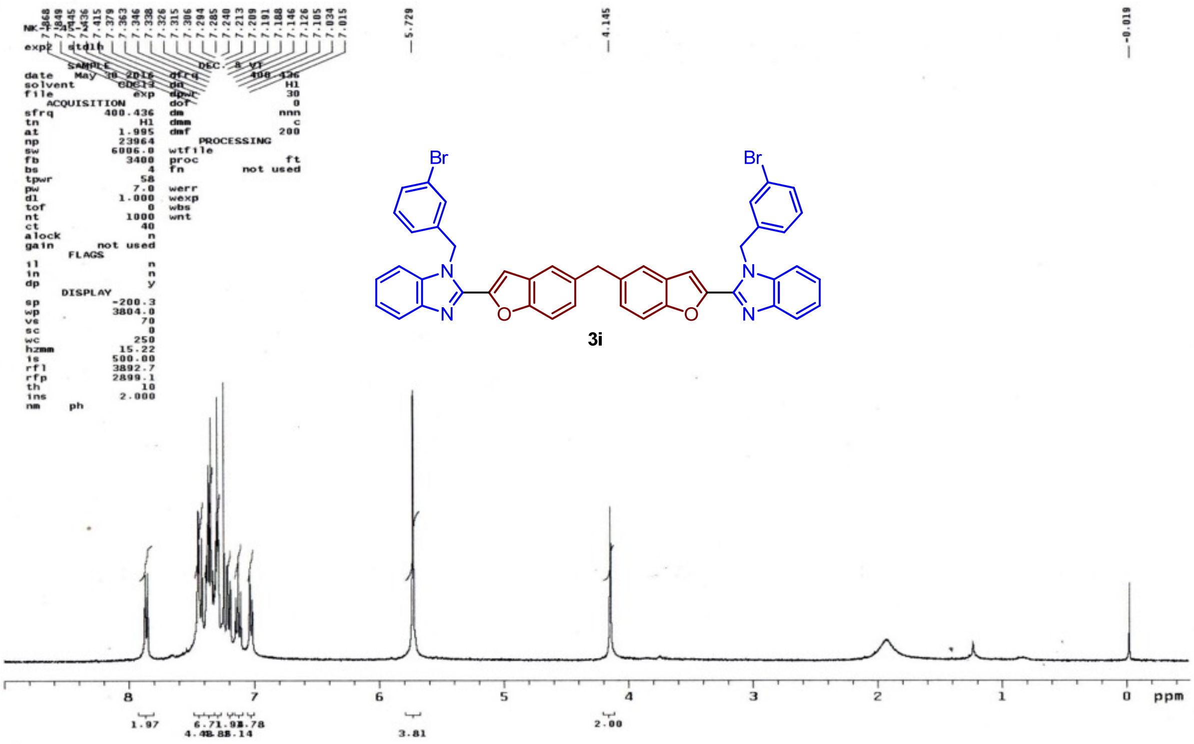

3

^{1}H NMR spectrum of compound **3i**

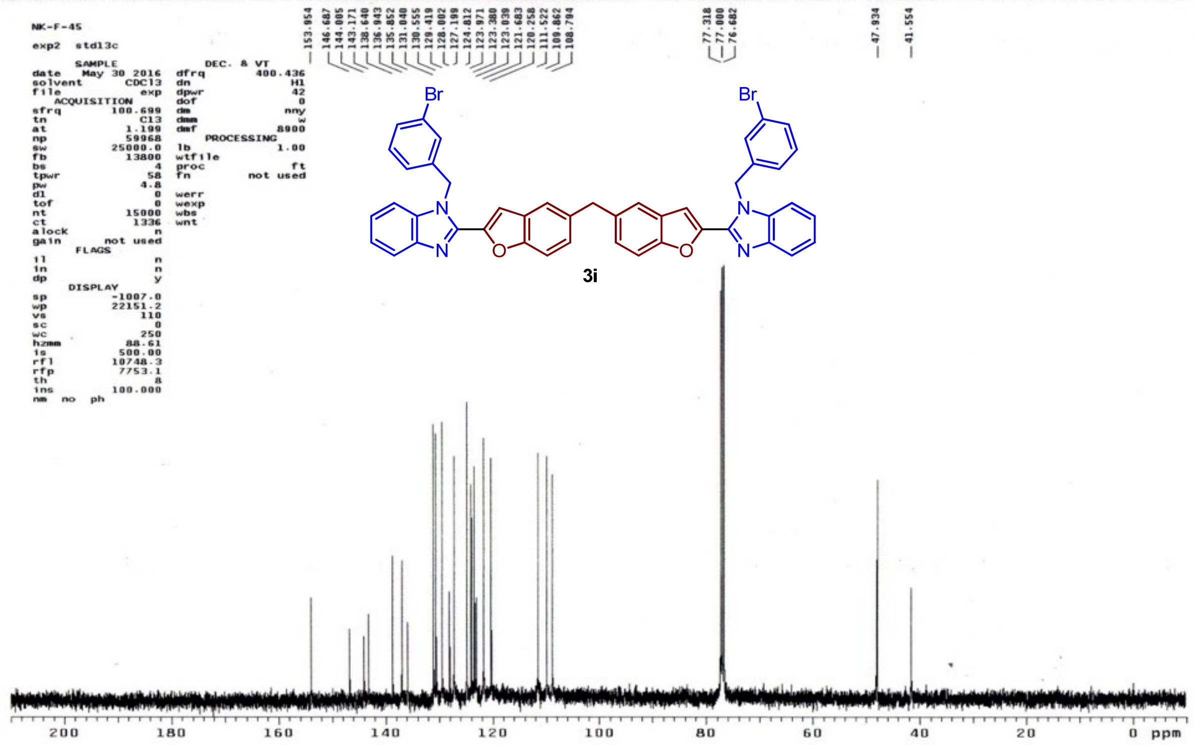

^{13}C NMR spectrum of compound **3i**

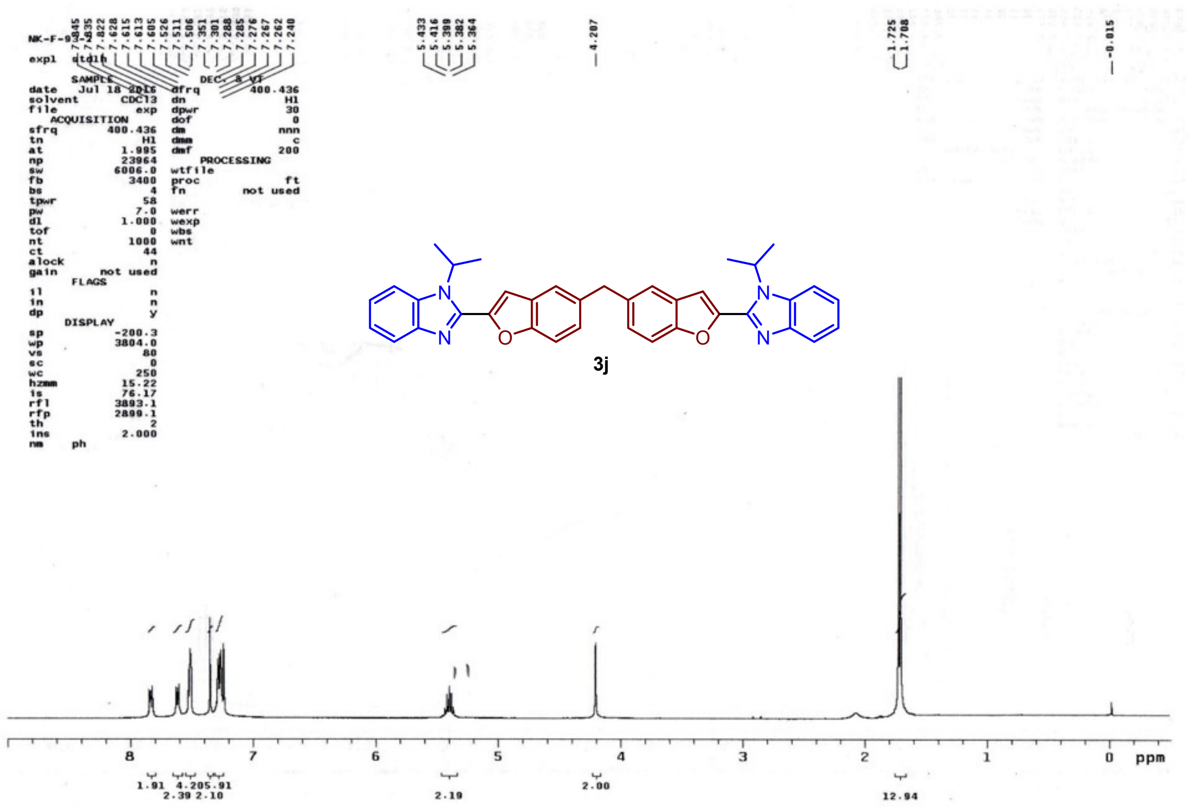

^{1}H NMR spectrum of compound **3j**

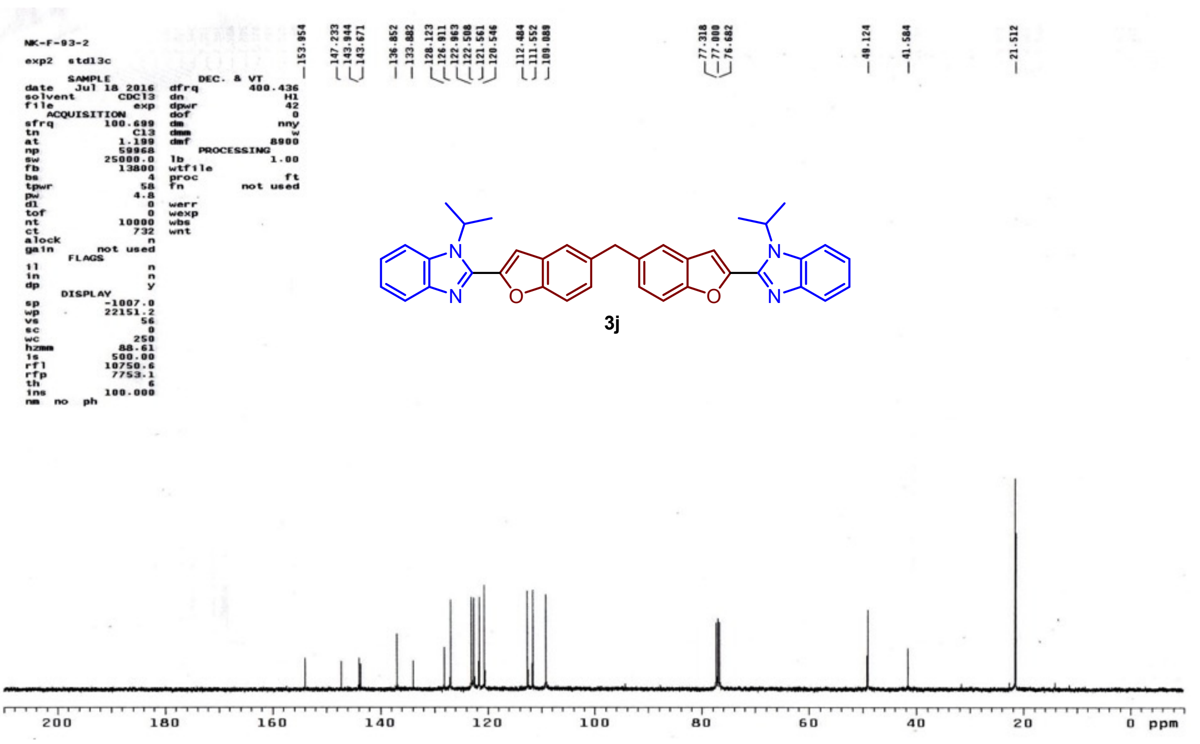

^{13}C NMR spectrum of compound **3j**

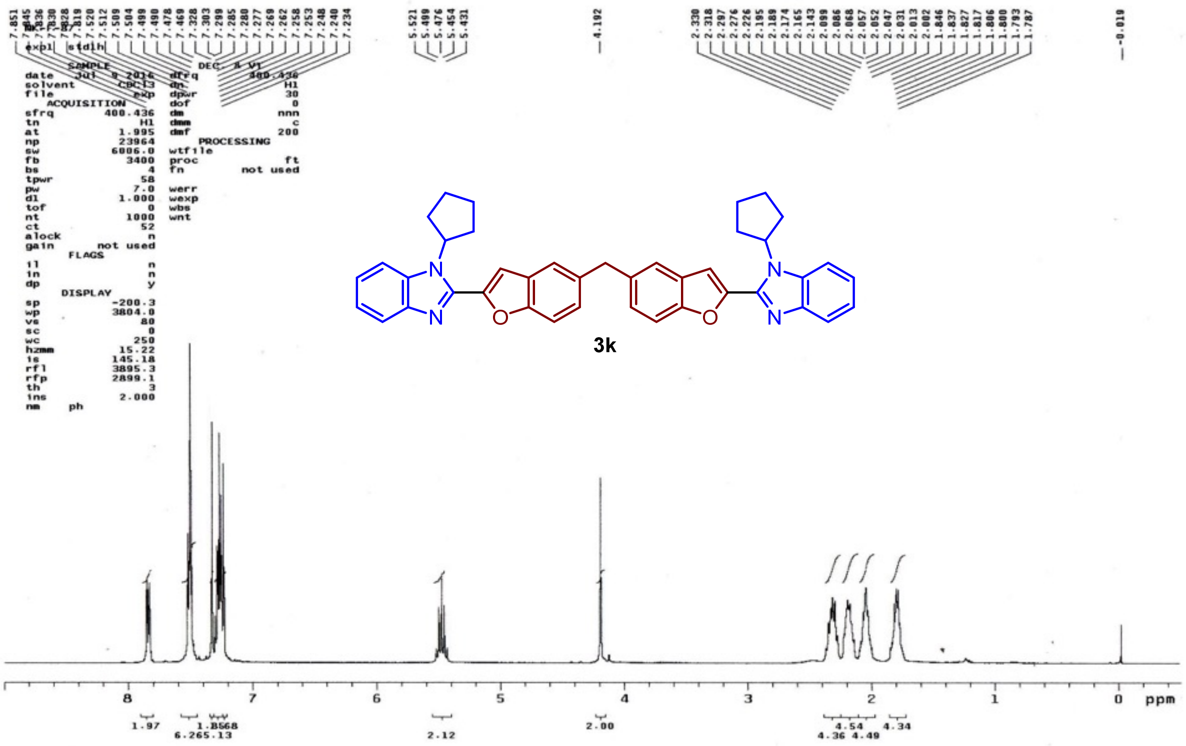

^{1}H NMR spectrum of compound **3k**

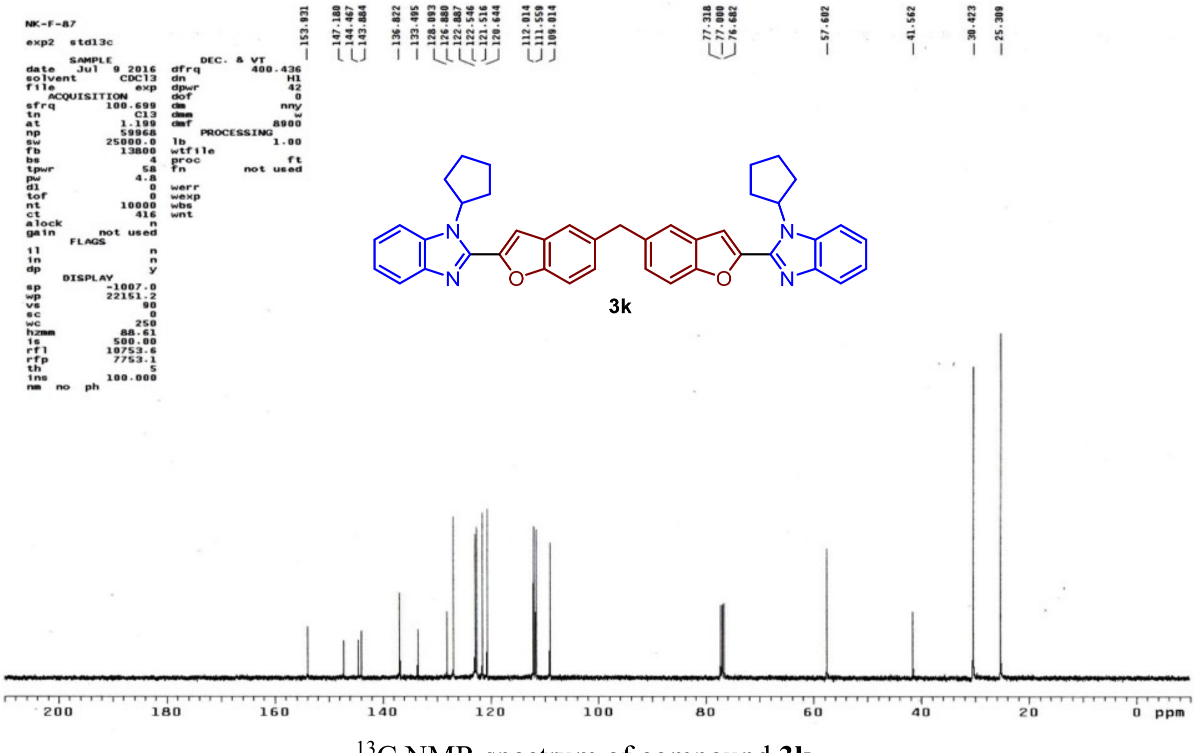

^{13}C NMR spectrum of compound **3k**

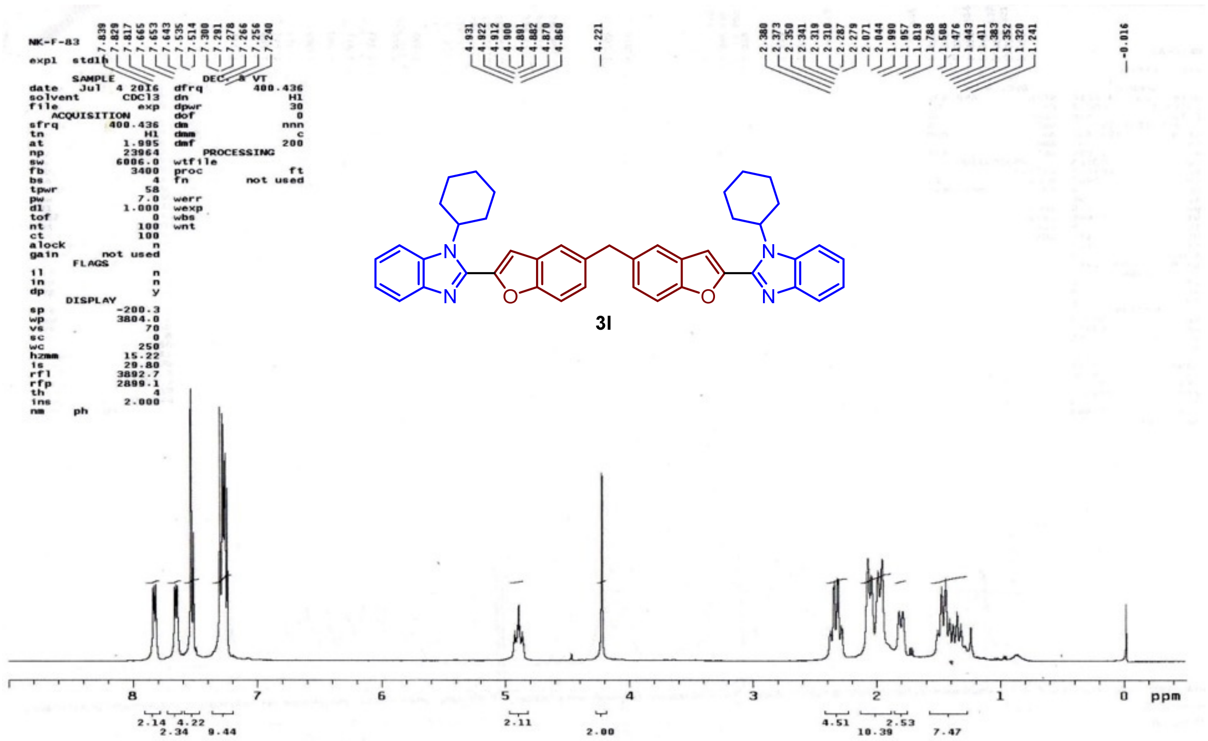

^{1}H NMR spectrum of compound **3l**

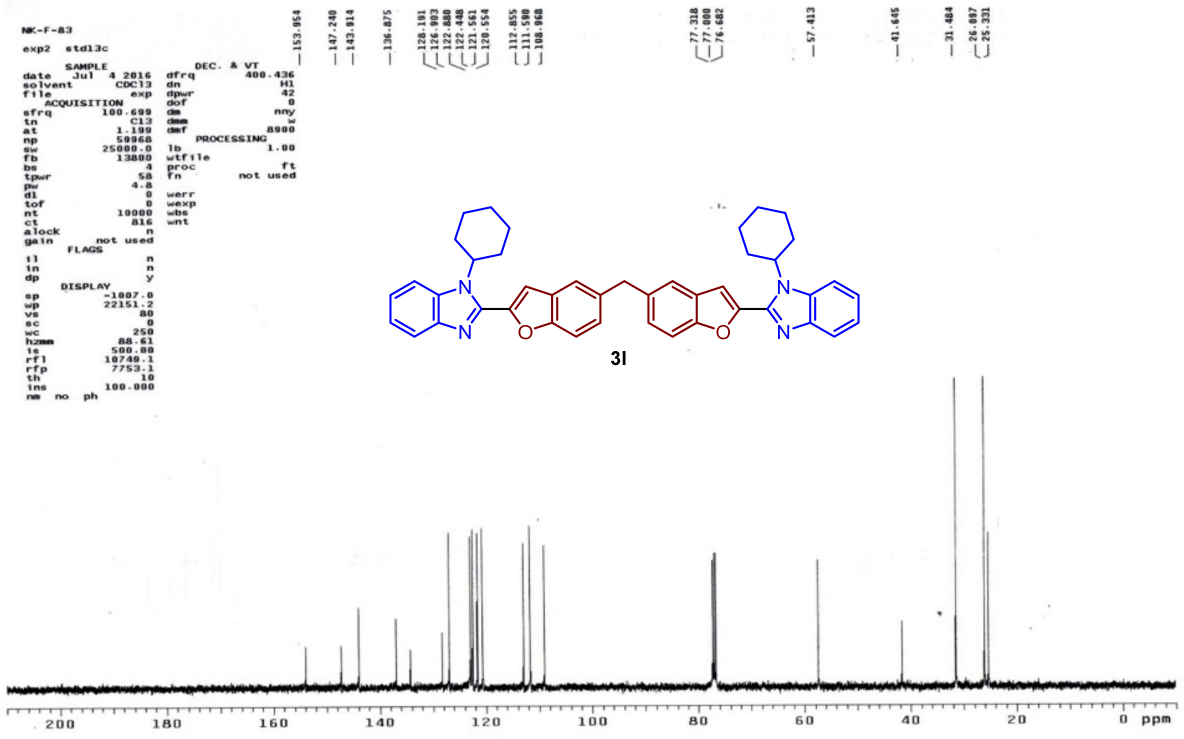

^{13}C NMR spectrum of compound **3l**
